# A Glucose-Responsive Glucagon-Micelle for the Prevention of Hypoglycemia

**DOI:** 10.1021/acscentsci.4c00937

**Published:** 2024-10-02

**Authors:** Daniele Vinciguerra, Rajalakshmi P S, Jane Yang, Panagiotis G. Georgiou, Katherine Snell, Théo Pesenti, Jeffrey Collins, Mikayla Tamboline, Shili Xu, R. Michael van Dam, Kathryn M. M. Messina, Andrea L. Hevener, Heather D. Maynard

**Affiliations:** † Department of Chemistry and Biochemistry, University of California, Los Angeles, 607 Charles E. Young Drive East, Los Angeles, California 90095-1569, United States; ‡ California NanoSystems Institute, University of California, Los Angeles, 570 Westwood Plaza, Los Angeles, California 90095-1569, United States; § Department of Molecular and Medical Pharmacology and Crump Institute for Molecular Imaging, David Geffen School of Medicine, University of California, Los Angeles, Los Angeles, California 90095-1735, United States; ∥ Jonsson Comprehensive Cancer Center, David Geffen School of Medicine, University of California, Los Angeles, Los Angeles, California 90095-1735, United States; ⊥ Department of Medicine, Division of Endocrinology, David Geffen School of Medicine at UCLA, 650 Charles E. Young Dr, Los Angeles, California 90095, United States; # VA Greater Los Angeles Healthcare System GRECC, Los Angeles, California 90073, United States

## Abstract

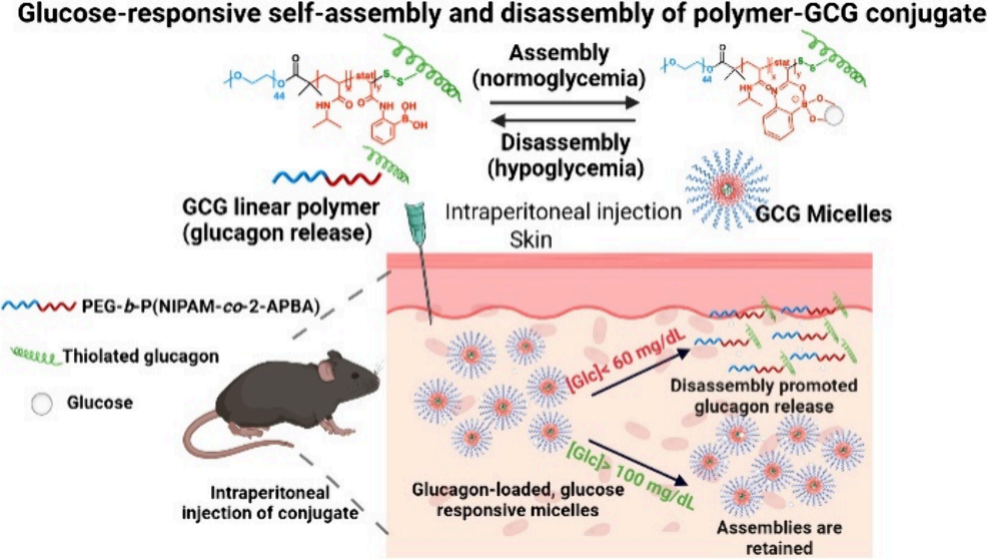

While glucose-responsive insulin delivery systems are in widespread clinical use to treat insulin insufficiency, the on-demand supplementation of glucagon for acute hypoglycemia treatment remains understudied. A self-regulated glucagon release material is highly desired to mitigate the potential risks of severe insulin-induced hypoglycemia. Here, we describe a glucose-responsive polymeric nanosystem with glucagon covalently grafted to the end-group. Under normoglycemic conditions, phenylboronic acid units in the polymer chain reversibly bind glucose, triggering self-assembly of the conjugate into micelles. During hypoglycemia, however, the micelle disassembles into its original, unimeric state, revealing the active glucagon conjugate. The formulation showed a 5-fold increase in activity compared to native glucagon when tested *in vitro*. Glucagon-loaded micelles injected into mice prevented or reversed deep hypoglycemia when administered prior to or during an insulin challenge. Glucagon release was only observed at or below the counterregulatory threshold and not during normoglycemia or moderate hypoglycemia. The *in vivo* acute and chronic toxicity analysis, along with μPET/μCT imaging, established the biosafety profile of this formulation and demonstrated no organ accumulation. This proof-of-concept work is the first step toward development of a translational, stimuli-responsive glucagon delivery platform to control glycemia.

## Abstract 2

Glucagon was conjugated to a glucose-responsive polymer, forming micelles at normoglycemia and exposing the peptide at a low blood glucose level, which prevented or reverted deep hypoglycemia.

## Introduction

Diabetes is a metabolic disorder caused by a decline in the function of insulin-producing *beta-*cells in the pancreas (type 1, T1D) or by peripheral insulin resistance and reduced *beta-*cell mass (type 2, T2D).^1,2^ Together T1D and T2D affect more than 500 million people worldwide and are projected to increase to 1.3 billion by 2050.^3−5^ In healthy individuals, pancreatic cells dynamically respond to blood glucose fluctuations by stimulating insulin secretion from the pancreatic *beta-*cells.^6,7^ During hypoglycemia, insulin secretion is inhibited, restoring glycemic homeostasis *via* the release of glucagon from pancreatic *alpha-*cells (epinephrine and norepinephrine are also released from the adrenals) to stimulate the production of glucose predominantly by the liver, and kidney to a smaller extent.^8^ Glucagon (GCG) is an endogenous peptide that raises blood glucose levels by activating hepatic gluconeogenesis and glycogenolysis.^9^ Because glucose homeostasis is disrupted in diabetic patients, treatment generally involves regular insulin replacement to combat rising blood glucose, hyperglycemia. Although insulin treatment is extremely effective in lowering blood glucose from dangerous highs, episodes of moderate to severe hypoglycemia are common clinical complications. In such cases, where endogenous glucose counterregulation is inadequate to combat hyperinsulinemia and restore normoglycemia, glucagon is administered as an emergency treatment to prevent clinical symptoms including malaise, cognitive impairment, seizure, and coma.^10^

Eli Lilly commercialized the first glucagon kit using a lyophilized powder that required reconstitution in an acidic solution before injection,^11^ but it was discontinued in 2022.^12^ Recent advances in glucagon delivery have included solubilizing the peptide at alkaline pH with excipients, creating new formulations with known stabilizers, and developing new additives and delivery vehicles.^13−16^ Current FDA-approved glucagon delivery systems are a nasal spray^17^ and a prefilled syringe of glucagon in dimethyl sulfoxide with sulfuric acid.^11,18,19^ Other efforts included developing glucagon analogues, one of which is FDA approved.^20−22^ Despite these advancements, a majority of research has been focused on emergency treatments for patients already experiencing low levels of glucose. A paradigm shift in the glucagon treatment strategy would be to prevent hypoglycemia altogether. Haidar et al. explored this concept in a randomized crossover-controlled trial, revealing that diabetic adults achieved better glucose control and reduced hypoglycemia risk when using a closed-loop, dual-hormone pump.^23^ Since then it has been shown that repeated doses of glucagon do not cause hepatic glycogen depletion, and glucose responsiveness to glucagon administration was similar regardless of prior glucagon administration.^24−26^ These data suggest that smart delivery systems that sense low glucose levels to release glucagon might be possible as preventative treatments for hypoglycemia.

There are many glucose-responsive materials that detect and respond to high levels of glucose for insulin delivery.^27^ Most commonly these contain components such as phenylboronic acids (PBAs) or glucose oxidase (GOx) that sense glucose.^28,29^ PBA and its derivatives selectively bind to glucose by dynamic, covalent interactions with the 1,2 diols and have been widely used to synthesize functional polymers and nanomaterials for insulin delivery.^30−33^ Typically, meta- and para-substituted PBAs are utilized, where the addition of glucose stabilizes the charged tetrahedral glucose boronate esters, increasing the ionization degree of the polymer and its hydrophilicity, resulting in the disassembly of the materials,^34,35^ while GOx reacts with glucose, changing, for example, the local pH, which in turn solubilizes or swells materials to release insulin.^36^ However, efforts toward detection of low glucose levels for glucagon delivery are limited. Notable work has been conducted using glucose-responsive microneedle patches to deliver both glucagon and insulin, primarily pioneered by Gu,^37−39^ Wu,^40,41^ and co-workers. Webber and co-workers successfully designed a nanofibrillar assembly that uses GOx to form a nonequilibrium peptide hydrogel by lowering the pH through glucose consumption. When glucose levels are low, the neutral environment triggers gel dissolution, leading to the release of an analog of glucagon, dasiglucagon.^42^ Recently, the same research group reported the formation of droplets through liquid–liquid phase separation by a net-cationic supramolecular peptide amphiphile with a glucose-binding motif and dasiglucagon in the presence of glucose, with dasiglucagon being released in the absence of glucose.^43^ These are exciting approaches; however, the authors also reported background release of dasiglucagon. Therefore, there is still interest in developing new responsive glucagon systems.

**Scheme 1:**
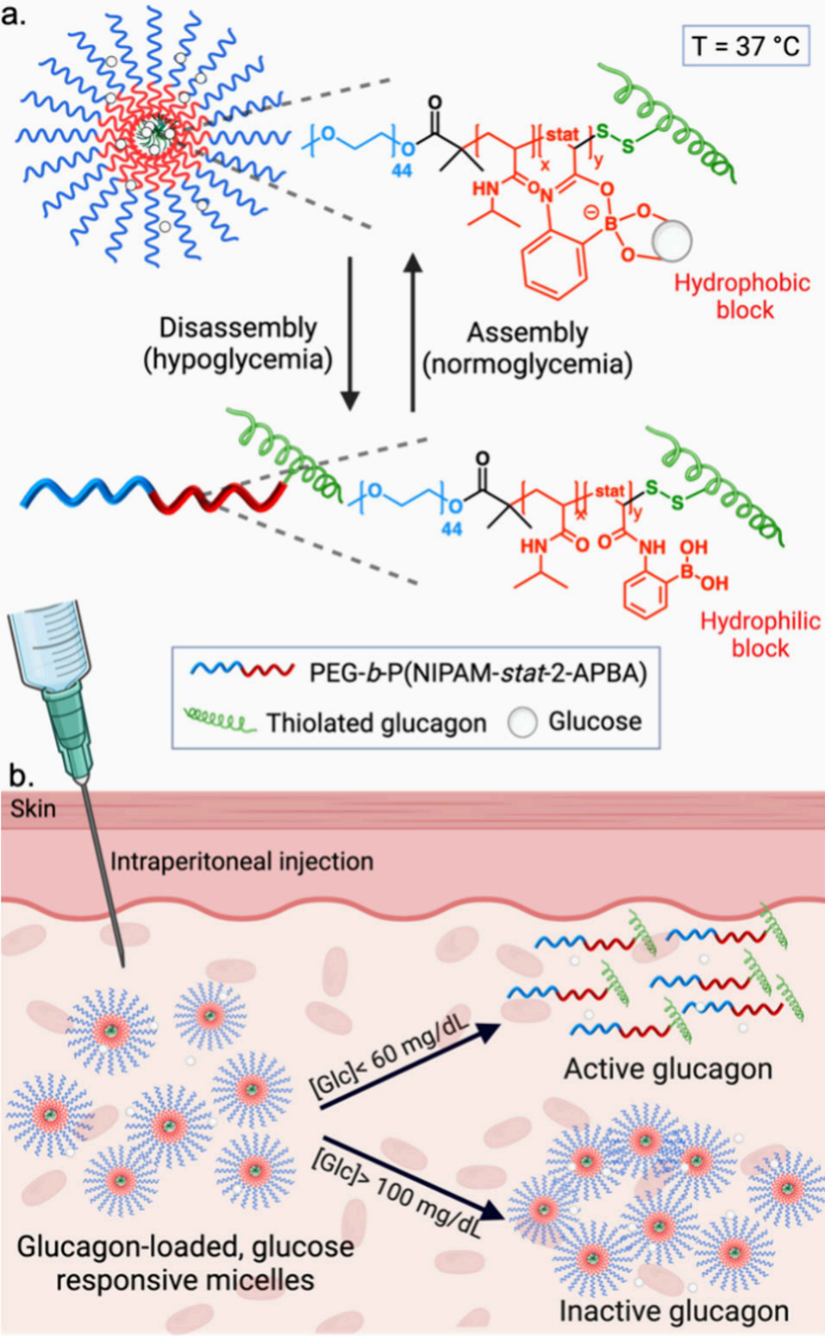
Schematic of Glucagon-Polymer Conjugate Mode of action overview of PEG-b-P(NIPAM-*stat*-2-APBA)-GCG between micellar form in normoglycemic conditions and linear (unimer) format in hypoglycemic conditions. (a) Glucose-responsive self-assembly and disassembly of PEG-b-P(NIPAM-*stat*-2-APBA)-GCG conjugate. Binding of glucose with the PBA group increases the hydrophobicity of the P(NIPAM-*stat*-2-APBA) block so that at 37 °C the polymer forms micelles. When glucose levels are lowered, the P(NIPAM-*stat*-2-APBA) core becomes more hydrophilic, and the micelle disassembles. (b) Exposure to normoglycemic conditions ([Glc] > 100 mg/dL), GCG micelles retain their nanoparticle format. Under exposure to deep hypoglycemia ([Glc] < 60 mg/dL), GCG-micelles disassemble promoting the release of GCG and subsequent increase of blood glucose concentration.

Herein, we report an approach utilizing micelles that release glucagon at low glucose levels (Scheme 1). The micelles exploited the use of 2-acrylamidophenylboronic acid (2-APBA). By placing the boronic acid in the ortho position to the amide, an intramolecular B–O dative bond is formed favoring the charged state.^44^ Opposite to the typical insulin delivery systems, upon the addition of glucose, the resultant material exhibits an increase in hydrophobicity. This feature has been exploited to alter the lower critical solution temperature (LCST) of a thermoresponsive *N*-isopropylacrylamide copolymer (P(NIPAM-*stat*-2-APBA)).^45^ LCST is the temperature at which a polymer transitions from its soluble, hydrated state to its shrunken, dehydrated phase. This transition heavily depends on the overall hydrophobicity of the chain (i.e., more hydrophobic polymers have lower LCSTs). Therefore, upon glucose addition to P(NIPAM-*stat*-2-APBA), the LCST decreases due to increased hydrophobicity^45^ Wang et al. exploited this to prepare poly(ethylene glycol)-*block*-poly(*N*-isopropylacrylamide-*stat*-2-acrylamidophenylboronic acid), PEG-*b*-P(NIPAM-*stat*-2-APBA), that self-assembled into micelles upon glucose addition at 30 °C.^46^ However, the glucose levels needed to induce this transition were orders of magnitude higher ([Glc] = 180 mg/dL) than what would be experienced in a physiological setting (<60 mg/dL, hypoglycemia to 100–150 mg/dL normoglycemia in rodents). Consequently, although this PEG-*b*-P(NIPAM-*stat*-2-APBA) micelle system was promising for hypoglycemia sensing, the polymer structure and molecular weight needed to be extensively refined to respond to physiologically relevant glucose levels. Therefore, in this work, a panel of polymers was synthesized *via* a chain extension of a PEGylated chain transfer agent by reversible addition–fragmentation chain-transfer (RAFT) polymerization. A thiolated glucagon was covalently grafted at the ω-polymer-end group *via* pyridyl disulfide exchange. At 37 °C in the presence of 150 mg/dL glucose (normoglycemia), the polymer-GCG conjugate candidate was micellar, and thus the glucagon was not active (Scheme 1). However, at 60 mg/dL (hypoglycemia) the micelles disassembled into linear/unimeric polymers revealing the active glucagon conjugate. The *in vivo* acute and chronic toxicity analysis and biodistribution determined through micropositron emission tomography (μPET)/microcomputed tomography (μCT) imaging showed the safety of this formulation. Blood glucose dynamics upon administration of glucagon-micelle in healthy mice demonstrated successful regulation of glucose levels by reverting and preventing insulin-induced hypoglycemia.

## Results

### Synthesis and Characterization of Polymer-GCG Conjugate Library

**Figure 1:**
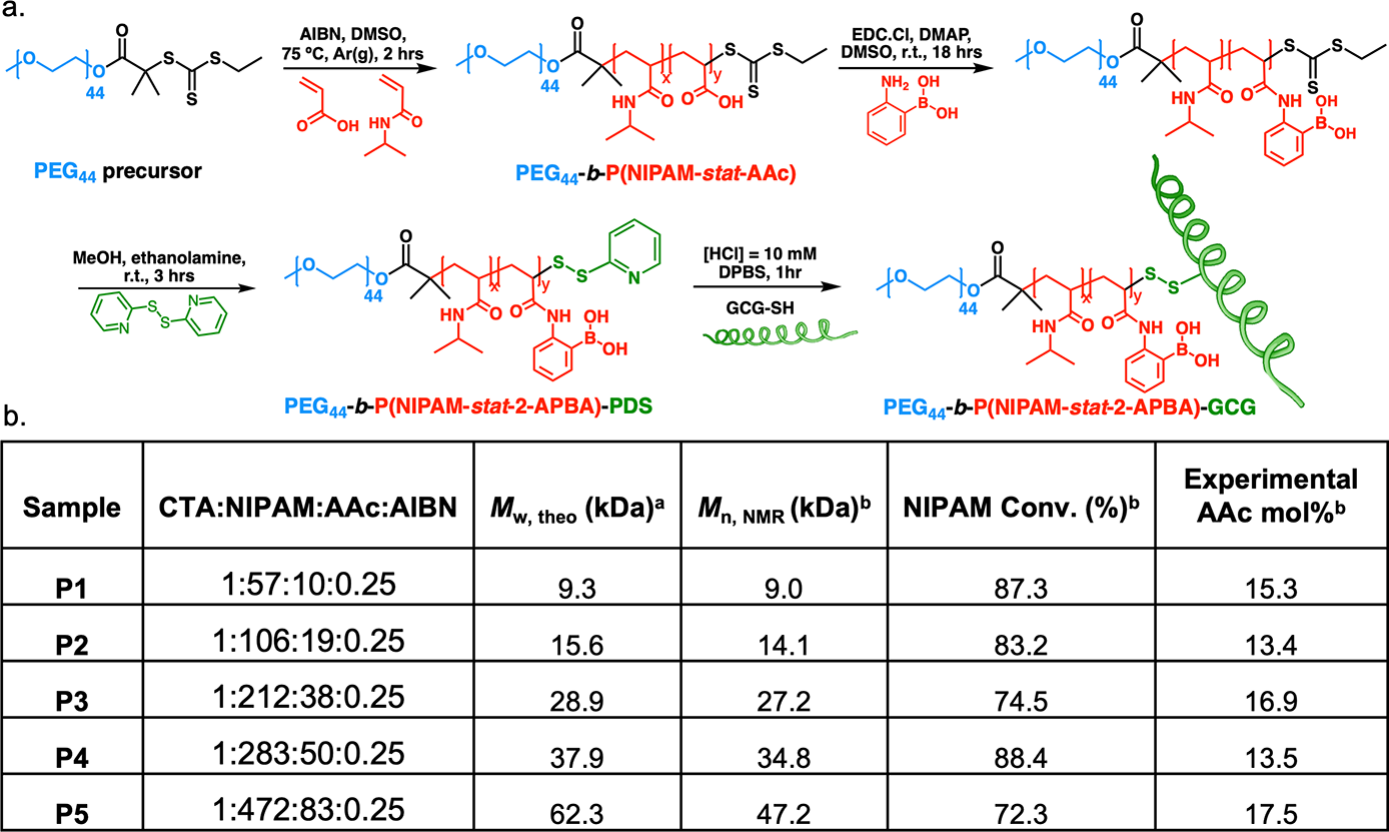
(a) Synthetic route of PEG-*b*-P(NIPAM-*stat*-2-APBA)-GCG (P2-GCG) conjugate (AIBN = 2,2′-azobis(2-methylpropionitrile), DMSO = dimethyl sulfoxide, EDC = *N*-3-dimethylaminopropyl)-*N*′-ethylcarbodiimide hydrochloride, DMAP = 4-(dimethylamino)pyridine, MeOH = methanol, HCl = hydrochloric acid). (b) Block copolymer characteristics of the PEG-*b*-P(NIPAM-*stat*-AAc) library. ^a^Determined assuming 100% chain extension efficiency. ^b^Determined by ^1^H NMR analysis in DMSO-*d*_6_.

Considering the instability and degradability of glucagon in aqueous media, our initial hypothesis was that a glucagon-polymer conjugate would promote stabilization at physiological pH as evidenced by the work of Stigsnaes et al. on PEGylated glucagon.^47^ Thus, a PEGylated corona was selected for micelle formulation. The P(NIPAM-*stat*-2-APBA) block was selected for glucose and thermoresponsiveness. A block copolymer library based on PEG-*b*-P(NIPAM-*stat*-2-APBA) with varying PEG to P(NIPAM-*stat*-2-APBA) block length ratios (while keeping the NIPAM:2-APBA molar ratio to 85:15 and PEG molecular weight at 2000 Da) was synthesized to fine-tune the glucose-responsiveness at 37 °C, specifically to make the micelles disassemble at 60 mg/dL. RAFT polymerization in DMSO was employed to copolymerize *N*-isopropyl acrylamide (NIPAM) and acrylic acid (AAc) using a PEG-modified at the omega-end with 2-(((ethylthio)carbonothioyl)thio)-2-methylpropanoic acid macromolecular chain transfer agent (PEG44 macro-CTA), yielding PEG-*b*-P(NIPAM-*stat*-AAc) (polymers P1–P5, *M*_n_ = 9.0–47.2 kDa; see Supporting Information for detailed synthetic conditions, Figure 1a–b, Figures S1, S2). EDC coupling was performed between 2-aminophenyl boronic acid and the carboxylic acid of PAAc repeat units (Figures S3–S12, Table S1). Aminolysis of the polymer terminal trithiocarbonyl group allowed for the installation of a thiol-reactive pyridyl disulfide (PDS) moiety at the ω-end group (Figures S13, S14). Figure 1a shows the overall synthesis scheme.

**Figure 2:**
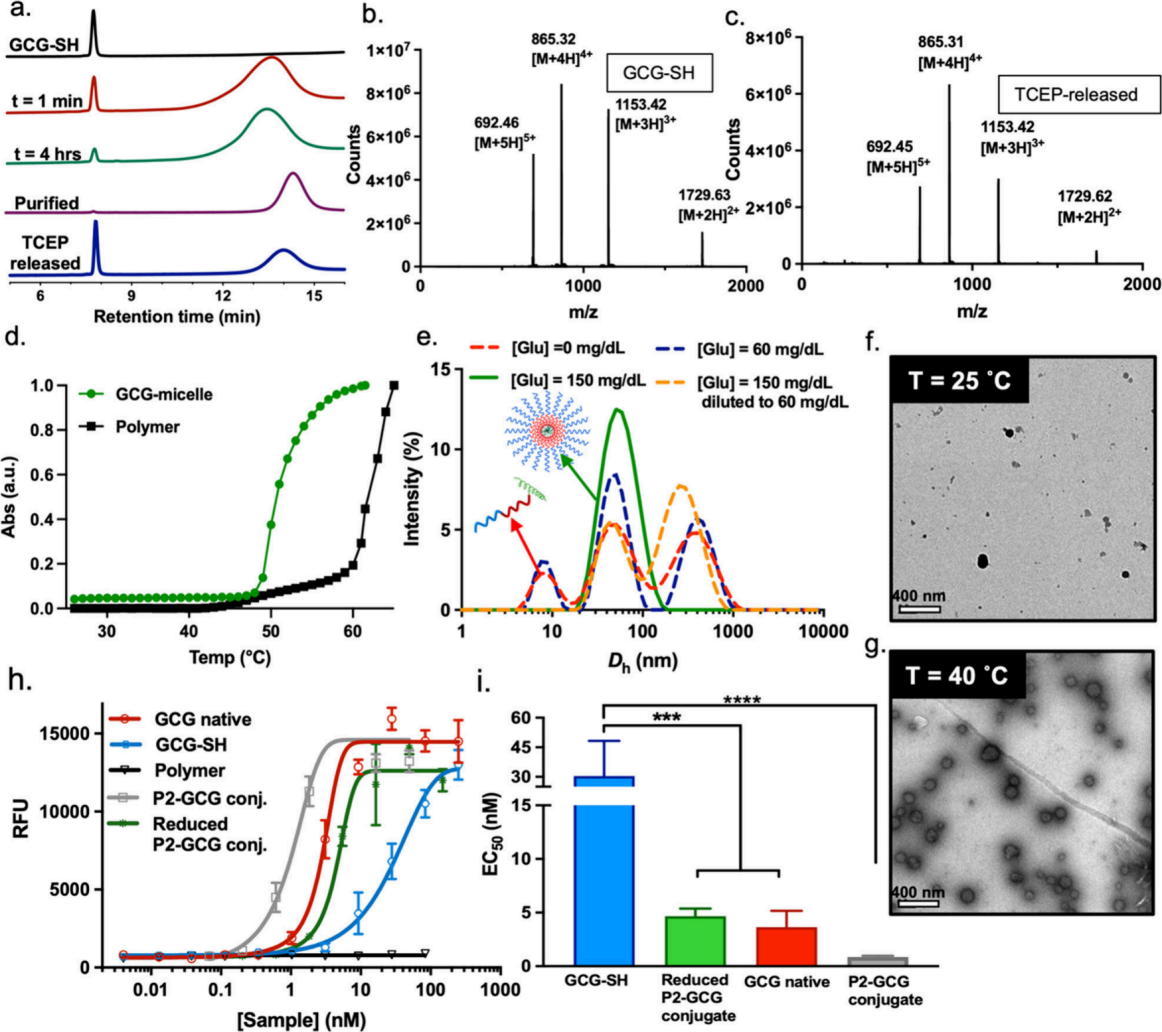
(a) GCG-SH conjugation to PEG-b-P(NIPAM-*stat*-2-APBA)-PDS monitored by HPLC at λ = 224 nm. (b) LC-MS mass spectra of fresh GCG-SH and (c) GCG-SH released from the conjugate with TCEP (10 mM). (d) Normalized cloud point (10 mg/mL) of block copolymer candidate (P2) before and after GCG-conjugation measured by UV–vis spectroscopy between 25 and 65 °C by determining absorbance at λ = 600 nm. (e) Intensity-weighted size distributions obtained by DLS for GCG-micelles at 37 °C showing micelle formation at normoglycemia and their disruption when the media is diluted to hypoglycemic level. [Glc] = 0 (red curve), 60 (purple curve), 150 (green curve), and 60 mg/dL upon dilution (orange curve). (f) TEM image of GCG-micelle at 25 °C, presenting no micelles and (g) at 40 °C, presenting defined micelles. (h) Dose response curves of native GCG (red), GCG-SH (blue), P2 Polymer (black), P2-GCG conjugate (gray), and TCEP reduced P2-GCG conjugate (green) using commercial kit cAMP Hunter eXpress GCGR CHO-K1 GPCR assay. (i) EC50 values of native GCG (red), GCG-SH (blue), P2-GCG linear conjugate (gray), and TCEP reduced P2-GCG in the linear form (green) using commercial kit cAMP Hunter eXpress GCGR CHO-K1 GPCR assay. Data are shown as the mean ± SEM of five to six independent repeats. *p* < 0.001 (***), *p* < 0.0001 (****).

A thiolated glucagon analogue bearing a cysteine residue (Q24C), GCG-SH, was used in this study and covalently conjugated onto the polymer end-group *via* pyridyl disulfide exchange and formation of a disulfide bond (Figure 1a). This chemistry was chosen due to the ease of synthesis of the pyridyl disulfide polymer from the trithiolcarbonyl group and because the polymer then reacts with the thiolated glucagon without any additional reagents. Circular dichroism studies on the native and thiolated glucagon and predicted 3D structures using AlphaFold indicated that the *alpha-*helix was fully retained (Figures S15, S16). PEG-*b*-(NIPAM-*stat*-2-APBA)-PDS block copolymers were reacted with GCG-SH in a 1.2:1 mixture of DPBS (pH = 7.4):HCl 10 mM (pH = 2). The reaction was monitored by high-performance liquid chromatography (HPLC) (Figure 2a). Within 1 min of the addition of GCG-SH to the PDS-containing polymer, 57.4% of the peptide was consumed, reaching a maximum conversion of 83.0% within 4 h.

To confirm the presence of GCG-SH in the conjugate, PEG-*b*-P(NIPAM-*stat*-2-APBA)-GCG was subjected to disulfide reduction with tris(2-carboxyethyl)phosphine (TCEP) for 30 min to release GCG-SH from the polymer. The amount released was quantified by HPLC and was consistent with the amount of GCG-SH consumed during the reaction. The integrity of released GCG-SH was also verified by LC-MS, indicating identical masses in excellent agreement with theoretical molecular weight (3457.76 Da). (Figure 2b-c). Lastly, the purity of PEG-*b*-P(NIPAM-*stat*-2-APBA)-GCG and the release of GCG-SH after reduction of the disulfide bond were visualized by SDS-PAGE stained with Coomassie (Figure S17). Altogether, these results confirm the successful synthesis of the conjugate PEG-*b*-P(NIPAM-*stat*-2-APBA)-GCG. Following this strategy, a library of glucagon-polymer conjugates (P1/P2/P3/P4-GCG) was prepared (Table S2). Conjugation conversion was consistent for all of the polymers.

### Solution Behavior of GCG-Polymer Conjugates: Tuning Glucose- and Thermo-Responsiveness

With this library of block copolymers (P1–P5), an initial screening of the thermoresponsive and glucose-responsive behavior of the unconjugated polymers was assessed by dynamic light scattering (DLS). In the absence of glucose, polymer samples P2–P4 retained their linear form at 37 °C, but upon increasing temperature to 40 °C, formed uniform micelles (Figure S18a, Table S2). In contrast, the largest molecular weight polymer, P5, formed micelles at 37 °C, confirming the trend of lower LCSTs with increased hydrophobic block molecular weight. As a next step, the glucose responsiveness of polymers P2–P4 was examined by DLS due to their linearity at physiological temperature, speculating that glucose addition could possibly induce their self-assembly into micelles. Both P2 and P3 did not form well-defined micelles in the presence of [Glc] = 150 mg/dL (Figure S18b). However, P4 exhibited self-assembly behavior at normoglycemic conditions ([Glc] = 150 mg/dL) (Figure S19a-b), forming uniform micelles with sizes similar to those obtained at 40 °C. Our observations confirmed that glucose effectively shifted the LCST to lower temperatures. Variable-temperature ultraviolet visible (UV–Vis) spectroscopy was also employed to evaluate the cloud point of P3 and P4 in different glucose concentrations ([Glc] = 0–1000 mg/dL) (Figure S20). A difference of 2.4 and 3.8 °C between cloud points of 0 and 1000 mg/dL of glucose was found for P3 and P4, respectively, confirming that glucose addition does shift LCST and micelle formation to lower temperatures.

The solution behavior of the P4-GCG conjugate was primarily investigated as P4 showed optimal glucose sensitivity at physiological temperature. However, when examined by DLS, it was demonstrated that it self-assembled into micelles at 37 °C without any glucose addition, unlike its unconjugated version (Figure S15b). In addition, these micelles had a significantly higher hydrodynamic diameter (*D*_h_ = 119.6 nm) compared to the diameter of micelles obtained from polymer without glucagon (*D*_h_ = 46.75 nm). Variable-temperature UV–Vis spectroscopy also confirmed that the cloud point of P4-GCG was shifted 2.5 °C lower than that of P4 alone, confirming the assembly behavior observed by DLS (Figure S21a-b). We speculate that because glucagon has a net charge of 0 at neutral pH, the peptide adds hydrophobicity to the conjugate, therefore shifting PEG-*b*-(NIPAM-*stat*-2-APBA)-GCG to a lower LCST. This trend was confirmed for P2-GCG as well, exhibiting a cloud point of 50.6 °C, 11.5 °C lower than that of P2 alone (Figure 2d). Dry-state transmission electron microscopy (TEM) of P2-GCG also confirmed the thermoresponsiveness of the system, showing no micelles at 25 °C (Figure 2f), while at 40 °C micelles were observed (Figure 2g). This effect is more marked with P2-GCG because of its lower molecular weight compared to that of P4-GCG, thus allowing glucagon to exert a more significant hydrophobic contribution. P3-GCG, however, behaved differently, showing an increase in cloud point by 9.5 °C higher than P3 alone (Figure S22a). Both the polymer and conjugate started eliciting a response to the rise in temperature around 45–46 °C, but the P3 response was much sharper. This may be attributed to a higher hydration of the conjugate, but further investigation is beyond the scope of this work. Nonetheless, when investigated by DLS, P3-GCG showed the same thermoresponsive behavior as P4-GCG and self-assembled into micelles already at 37 °C without glucose addition (Figure S22b).

Conjugate P2-GCG showed responsive characteristics by DLS (Figure 2e). Specifically, at 37 °C, P2-GCG had a mixture of linear conjugate, micelles, and larger structures, which could be attributed to swollen micelles in the absence of glucose or at hypoglycemic glucose concentrations ([Glc] = 60 mg/dL). It should be noted that DLS cannot be used to quantitatively assess the number of each species; especially since intensity percentage tends to overestimate the presence of larger particles. Importantly, DLS showed that well-defined micelles were present at normoglycemia ([Glc] = 150 mg/dL) with a hydrodynamic diameter of 50.39 nm (Table S2). To further investigate the sensitivity of this system toward changes in glucose concentration, P2-GCG micellar solution was diluted ([Glc] = 60 mg/dL). Within 10 min of incubation, the micelle peak decreased and a peak at 293 nm, which could be attributed to swelled micelles, was observed (Figure 2e). The conjugation was conducted for 4 h at 4 °C, which should not cause aggregation of the GCG. However, to investigate this, glucagon fibrillation was evaluated qualitatively by assessing fluorescence using the ThT assay, a dye known to bind to amyloid fibrils *in vitro*.^16^ The GCG-micelle exhibited no statistically significant increase in fluorescence after conjugation to P2. The stability of glucagon at temperatures above the LCST was also evaluated, as the polymer becomes more hydrophobic under these conditions. There was also no increase in fluorescence compared to that of fresh GCG-micelle after two cycles of heating above the LCST of the polymer (Figure S23). The data demonstrate that glucagon is stable during both conjugation and transition through the LCST. Considering these results, P2-(GCG) was selected as the candidate polymer for further *in vivo* toxicity, biodistribution, and efficacy evaluation.

### *In Vitro* Evaluation of PEG-b-P(NIPAM-stat-2-APBA)-GCG Conjugate Activity

A cAMP HunterTM eXpress GCGR CHO-K1 GPCR commercial test kit was used to evaluate the *in vitro* effectiveness of native GCG, GCG-SH, P2, P2-GCG, and TCEP reduced P2-GCG (Figure 2h,i). The kit uses cells in which human glucagon receptor (GCGR) is overexpressed, allowing for quantification of receptor activation *via* increased levels of intracellular cAMP. Native GCG showed an EC_50_ of 4.49 ± 0.52 nM, comparable to literature-reported values.^48,49^ The EC_50_ of GCG-SH was 39.30 ± 11.60 nM; the increase in EC_50_ is attributed to the chemical modification of GCG-SH, resulting in a reduced receptor interaction. However, the value for GCG-SH is still in the reported range for glucagon activity, and we believe that careful selection of the modification site in the peptide chain (Q24C) maintained significant bioactivity. Polymer P2 was used as a negative control, showing no bioactivity as expected.

The EC_50_ of the linear polymer conjugate P2-GCG (i.e., not in the micellar form) showed the lowest value (0.87 ± 0.12 nM). These results confirm that the glucagon is still active after conjugation to the polymer, and reduction of the conjugate disulfide to release GCG-SH is not a requisite for receptor activation. This is in accordance with previous findings by our laboratory, where glucagon covalently linked to trehalose nanogels, exerted activity independent of polymer release.^15^ The assay confirmed that the critical step for the onset of activity is micelle disassembly to unmask the glucagon conjugate rather than GCG-SH release. Notably, the EC_50_ of GCG-P2 is 5-fold lower than that of native glucagon, indicating that the polymer might promote receptor interaction or simply enhance the stabilization of the peptide. To confirm this hypothesis, we reduced the conjugate’s disulfide bond using a 10 mM TCEP solution (TCEP removed through centrifugal filtration, molecular weight cutoff (MWCO) = 3.5 kDa), leading to a mixture of GCG-SH and polymer, P2. The reduced solution showed an EC_50_ of 4.66 ± 0.64, 5-fold higher than that of unreduced P2-GCG and on the same order of magnitude as that of native GCG. This activity loss upon reduction confirms that the conjugate is a more potent agonist than native GCG.

### *In Vivo* Acute and Chronic Toxicity of Empty Micelles (P2)

Prior to study of the glucagon conjugate, acute toxicity of the empty micelles (candidate polymer P2) was evaluated in C57Bl/6J male mice at 0, 24, and 120 h following injection. Mice were injected intraperitoneally (IP) with a single dose of empty micelles (2.322 mg/kg, equivalent to 500 μg/kg of glucagon) and were euthanized at different intervals to determine the complete blood count (CBC) (*n* = 4 or 5) and organ weights (liver, kidney, spleen, heart, and lungs) (*n* = 6). White blood cell (WBC), red blood cell (RBC), monocytes, lymphocytes, neutrophils, eosinophils, hemoglobin, and hematocrit were measured (Figure S24a and Table S3). No significant difference in CBC counts was observed when compared to mice treated with empty micelles after 0, 24, and 120 h. All values were in accordance with the reported literature.^50−52^ Additionally, there were no significant differences between the treatment groups for organ weights (Figure S24b).

Following acute studies, a chronic toxicity study was conducted in C57Bl/6J mice. The mice were injected IP with empty micelles every day for 14 days, and the control group was injected with saline (Figure S25a). After 14 days of injections, mice were euthanized to determine body weight, CBC count, organ weight, histopathology, hepatic function parameters, kidney function parameters, immune markers, and immunohistochemistry of lung and liver tissues. Total body weights as well as organ weights of saline control and empty micelle-treated mice (liver, lungs, spleen, heart, and kidney) showed no significant differences (Figure S25b). The CBC counts were all in accordance with published values (Figure S26a,b (i)–(xii), Table S4).^50−52^ Hepatic function parameters including alanine aminotransferase (ALT) and aspartate aminotransferase (AST) levels analyzed from serum (Figure S26d and Table S5) were the same for the micelles as the negative controls. For lactate dehydrogenase (LDH), the values for the empty micelles were slightly lower and statistically significant compared to the control. Kidney function was determined by blood glucose level and calcium count in urine and found not to be different for mice exposed to the micelle (Figure S26c). Organ histopathology of liver, spleen, heart, lung, and kidney were examined *via* Hematoxylin & Eosin (H&E) staining (Figure S26e). No microscopic morphological changes were observed in the histological sections. Markers IFN γ, TNF α, and IL 2 were also analyzed to determine immune reaction and inflammation in response to micelle injection, and no differences were detected between the groups (Figure S27a and Table S6). Immunohistochemistry was performed on liver and lung slices by using a macrophage marker F4/80 antibody. No difference in F4/80 staining was observed for either tissue between the groups (Figure S27b). Together, the data for acute and chronic toxicity analyses demonstrate a favorable safety profile of the polymeric material.

### *In Vivo* μPET/μCT Imaging of PEG-b-P(NIPAM-*stat*-2-APBA) Micelle and GCG-SH

**Figure 3:**
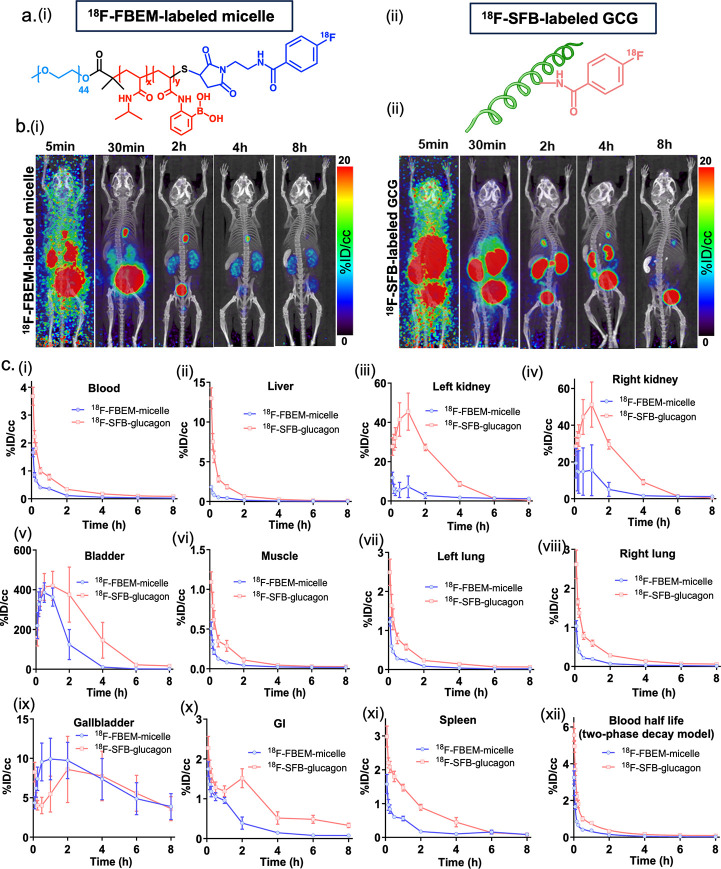
μPET-μCT imaging showing the time course biodistribution and excretion of ^18^F-FBEM-labeled-micelle and ^18^F-SFB-labeled GCG (*n* = 4). (a) Chemical structure of (i) ^18^F-FBEM-labeled micelle and (ii) ^18^F-SFB-labeled GCG. (b) PET-CT images of (i) ^18^F-FBEM-labeled micelle and (ii) ^18^F-SFB-labeled GCG. (c) The time course biodistribution in blood, liver, left kidney, right kidney, bladder, muscle, left lung, right lung, gall bladder, gastrointestine (GI), spleen, and blood half-life time. ID, injected dose. CC, cubic centimeters.

The μPET/μCT imaging was performed by using an ^18^F-FBEM-labeled micellar polymer and ^18^F-SFB-labeled glucagon as a negative control. Conjugation conditions of the micelle and glucagon were initially optimized using ^19^F-FBEM/SFB (see Supporting Information for detailed experimental conditions on synthesis and conjugation of ^18/19^F-FBEM/SFB, Figures S33–S37, Figure 3a).

The μPET/μCT imaging analysis of ^18^F-SFB-labeled glucagon showed higher uptake in all organs compared to ^18^F-FBEM-labeled micelle, particularly in the kidney and spleen during the first 4 h after probe injection (Figures 3b,c, S38, S39). The ^18^F-FBEM-labeled micelle was eliminated by renal clearance more rapidly than glucagon, as shown by PET signal intensity in the bladder. In addition, the ^18^F-FBEM-labeled micelle was also rapidly cleared from the gallbladder. After 8 h postinjection, organs showing PET activity (>1%ID/cc) were kidney (^18^F-FBEM-labeled micelle 1.17%ID/cc), gallbladder (^18^F-FBEM-labeled micelle 3.88%ID/cc, ^18^F-SFB-labeled glucagon 3.61%ID/cc), and bladder (^18^F-FBEM-labeled micelle 1.44%ID/cc, ^18^F-SFB-labeled glucagon 16.75%ID/cc). These data contrast with what was observed when the micelles were labeled with ^89^Zr *via* a DFO chelator (see Supporting Information for details). Therefore, we hypothesize that uptake and accumulation seen with the ^89^Zr-radiolabel may have been directly caused by the radiometal and chelator.

### *In Vivo* Safety Evaluation of PEG-b-P(NIPAM-*stat*-2-APBA)-GCG Micelle

To investigate if P2-GCG elevates glycemia *in vivo* at normal glycemic conditions, the conjugate was administered IP in healthy 6 h fasted C57Bl/6J mice (Figure S40). The control group was administered with PBS only. Upon injection, the blood glucose levels of mice were monitored over 8 h at different time intervals. An initial small spike was observed in the first 15 min, with the glycemia level rising from 160 to 190 mg/dL. This is not uncommon, as the stress during injection can lead to a transient increase in blood glucose and is observed in both the glucagon-micelle and the PBS control. After 15 min, the glucose level steadily dropped to 125 mg/dL within 60 min of injection and remained constant until the end of the experiment. Overall, glycemia remained in the normal range throughout the study. Following the 8 h study period, animals were euthanized, kidneys and liver were harvested, and subsequent histopathological analyses were conducted *via* H&E staining (Figure S41). No microscopic morphological changes in histological sections were observed in micelle-injected mice vs controls. These findings indicate that micelles are not activated under normoglycemia, nor do they induce histopathological outcomes in the kidney or liver. Next, we evaluated the *in vivo* efficacy of the GCG-polymer conjugate.

### *In Vivo* Activity of PEG-b-P(NIPAM-*stat*-2-APBA)-GCG Micelles

**Figure 4:**
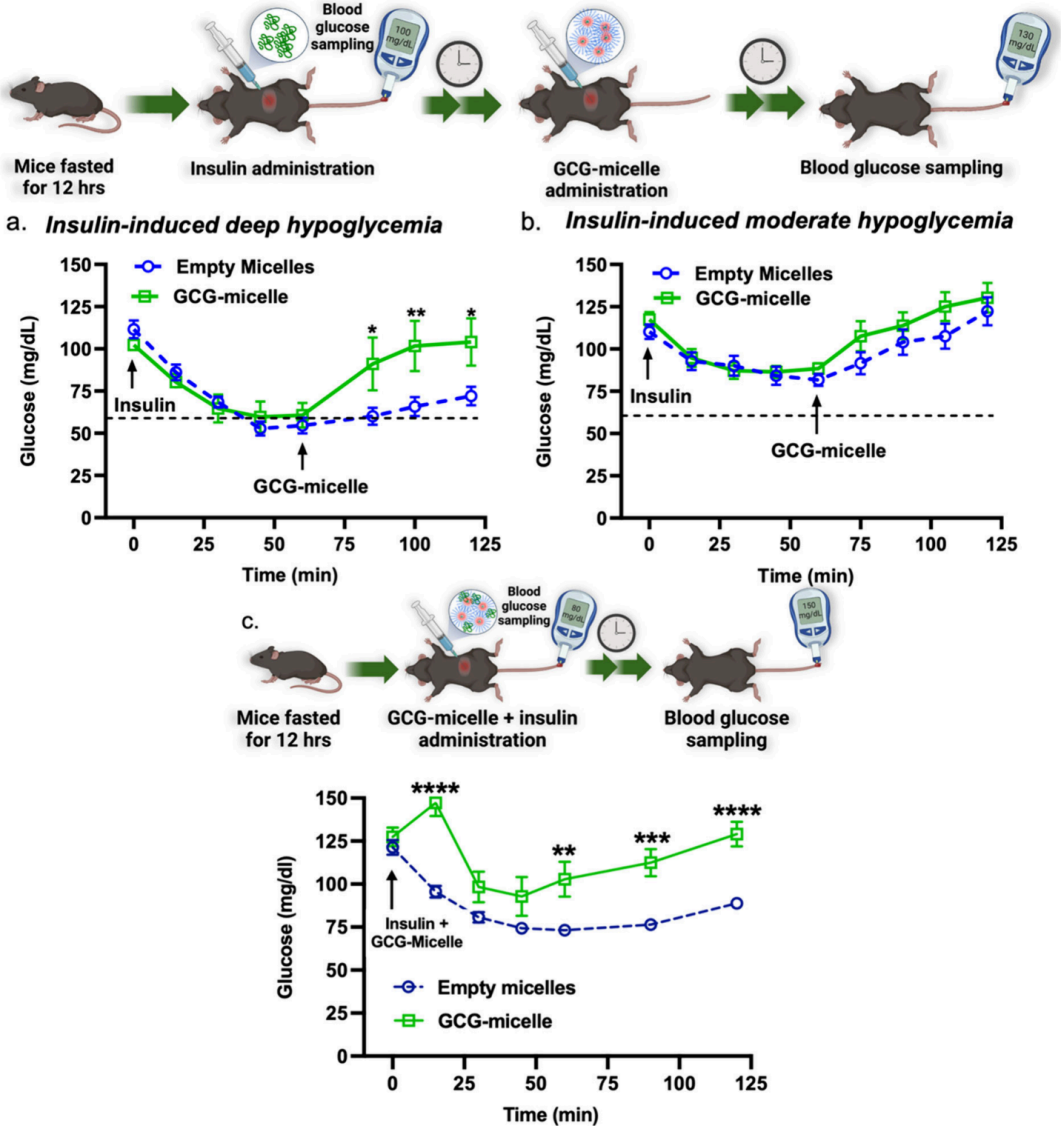
Micelle-reversal of insulin-induced deep (a) or moderate (b) hypoglycemia in fasted C57Bl/6J mice. The dotted line represents the approximate gluco-counterregulatory threshold. (c) Micelle-induced prevention of insulin-stimulated hypoglycemia in fasted C57Bl/6J mice. Data are represented as mean ± SEM, *n* = 5–6. *p* < 0.05 (*), *p* < 0.01 (**), *p* < 0.001 (***), *p* < 0.0001 (****). Statistical comparison of glucose levels between the two groups at the same time point during the ITT.

Two independent *in vivo* experiments were conducted to evaluate the effectiveness of P2-GCG in reversing or preventing insulin-induced deep hypoglycemia. The efficacy of GCG-micelle was evaluated in healthy male C57Bl/6J mice. First, the mice were fasted for 12 h prior to the administration of the insulin of dose 0.90 U/kg intraperitoneally to induce hypoglycemia. Glucose levels decreased from 100 to 65 mg/dL after 30 min of insulin administration and was approximately 60 mg/dL after 1 h. At this point, GCG-micelle at a dose of 500 μg/kg of glucagon was administered intraperitoneally. Blood glucose immediately increased, reaching 90 mg/dL in 25 min and 100 mg/dL within 40 min, restoring baseline glycemia (Figure 4a). For comparison, native and thiol-glucagon (GCG) alone (positive controls) and empty micelle (negative control) were also evaluated utilizing the same experimental protocol. The GCG-micelle showed a statistically different response compared to the negative control group injected with the empty micelle, which exhibited hypoglycemia <65 mg/dL until the end of the 120 min (Figure 4a), while the GCG-micelle had a similar response to the positive controls groups of native and thiol-glucagon (Figure S42). To evaluate the responsiveness of the GCG-micelle above the estimated glucose-counterregulatory threshold, moderate hypoglycemia (80 mg/dL) was induced by administering 0.85 U/kg insulin at time 0. The moderate reduction in glycemia, by design, should not trigger the disassembly of micelles to avoid the induction of hyperglycemia. After 60 min, 500 μg/kg of glucagon (GCG-micelle) was administered intraperitoneally resulting in a less pronounced response, restoring initial normoglycemia of 120 mg/dL in 105 min (within 45 min relative to micelle injection time). More importantly, there was no statistical difference in glycemia at any time point during the insulin tolerance test (ITT) between the GCG-micelle and the empty micelle-injected control. These data suggest that micelle disassembly was not triggered unless insulin induced a sufficient level of hypoglycemia at or below the estimated glucose counterregulatory threshold (Figure 4b). Overall, the GCG-micelles demonstrated *in vivo* glucose responsiveness that quickly and effectively reversed deep hypoglycemia, restoring blood glucose concentrations to baseline levels.

Next, the *in vivo* efficacy of the GCG-micelle to prevent deep hypoglycemia was assessed by injecting insulin and the micelles simultaneously (combined preparation) at time 0 of the experiment. Healthy male C57Bl/6J mice were fasted for 12 h before being administered 0.90 U/kg of insulin and either 500 μg/kg of glucagon (GCG-micelle) or an equivalent dose of empty micelle. The mice injected with the glucagon-conjugate maintained normoglycemia for the duration of the experiment, whereas mice injected with empty micelle showed a marked reduction in blood glucose that dropped below the counterregulatory threshold (Figure 4c). Specifically, blood glucose of empty micelle treated animals reached a nadir of 70 mg/dL at 60 min with endogenous counterregulation promoting only a modest increase in glycemia to 88 mg/dL at 120 min. This is in contrast to the GCG-micelle-treated mice in which baseline glycemia (130 mg/dL) was restored by 120 min. Overall, *in vivo* efficacy studies confirmed that the glucagon micelle can safely prevent and reverse deep hypoglycemia.

## Discussion

Glucose-responsive drug delivery systems have been extensively investigated for insulin delivery.^27^ However, glucose-responsive glucagon delivery is understudied. Inspired by the work of Wang and co-workers, we decided to use PEG-b-P(NIPAM-*stat*-2-APBA) as the polymer of choice for its ability to form micelles in the presence of glucose.^46^ We set out to tune this system to be responsive in physiological conditions, self-assembling, and disassembling in a narrow glucose range at relevant temperatures. Through RAFT polymerization, a library of block copolymers was synthesized with a trithiocarbonyl end-group that could be chemically modified to allow for the site-specific conjugation of glucagon. The LCST and glucose responsiveness were altered to be effective at physiological conditions; this was possible by varying weight ratios between the PEG hydrophilic block, which was kept constant at 2 kDa, and the P(NIPAM-*stat*-2-APBA) block. Initially, P4 was found to present responsivity at 37 °C and relevant glucose concentrations, forming well-defined, uniform micelles in normoglycemia, but when GCG-SH was conjugated, the polymer LCST decreased due to the hydrophobicity of the glucagon, forming micelles at temperatures lower than necessary. As a result, P2-GCG presented the optimal responsiveness, even though P2 alone did not.

In early experiments, we attempted to encapsulate native glucagon by physical entrapment (data not shown), but poor encapsulation and release were achieved. A covalent design was, therefore, pursued instead. Typically, polymer conjugation to a protein or peptide results in a loss of activity. To minimize this loss, researchers often employ site-selective conjugation to biomolecules.^53^ To best ensure site-selectivity during polymer conjugation to glucagon, we chose to engineer a single cysteine unit into the native peptide. First, we selected the substitution site for amino acid modification based on the crystal structure of a glucagon analogue binding to the glucagon receptor.^54^ Additionally, Chabenne et al. found that when substituting the existing glutamine residue of glucagon with alanine (Q24A), the full potency of glucagon receptor binding and activity was retained.^14^ We therefore selected the glutamine residue in position 24 to modify to a cysteine (Q24C) so that the peptide could be conjugated to a PDS-functionalized polymer. This allowed a simple, site-selective conjugation to the cysteine without affecting any of the other reactive residues of glucagon. Biomolecules conjugated to polymers often display reduced activity due to either undesirable changes in the protein structure itself or polymer interference between the biomolecule and its receptor. Much to our surprise, the *in vitro* experiments demonstrated a higher activity for the conjugate itself compared to both native, thiolated, and released GCG-SH after the reduction of the disulfide bond. We hypothesize that the polymer might help configure GCG in the correct confirmation for interaction with the receptor and the related G protein.^55^ Alternatively, the polymer might stabilize GCG in solution, similar to our previously published trehalose nanogels.^15^ Although unusual, other examples where site-specific protein–polymer conjugation increased protein bioactivity can be found in the literature.^56−59^ Ultimately, the increased conjugate activity remains under investigation yet is fortuitous.

The successful *in vitro* results prompted us to evaluate the formulation efficacy *in vivo*. First, the *in vivo* acute and chronic toxicity was evaluated to understand the safety of the formulation in a healthy mouse model. The *in vivo* acute toxicity was determined by analyzing the CBC count and organ weights. Empty micelles showed no significant difference in the CBC count or organ weights, indicating no acute toxic effects upon administration. Further, a detailed chronic toxicity analysis was performed for 14 days with daily dosages of empty micelles, examining organ weight, CBC count, hepatic and kidney function, histology, and immune/inflammation markers. The acute and chronic toxicity evaluation studies are promising in that they show a favorable safety profile over the dosages and time course studied. Although more extensive data of repeated administration over longer periods, as well as long-term toxicity effects will need to be obtained, and comprehensive immunogenicity studies, including antibody formation, are also planned in the future.

The *in vivo* μPET/μCT imaging experiments were performed to further understand the pharmacokinetics of the polymer. The μPET/μCT imaging using ^89^Zr-labeling revealed a small but unexpected percentage of polymer accumulated in certain organs. After hypothesizing that the hydrophobicity and large molecular weight of the DFO molecule may have led to the observed results, we decided to repeat μPET/μCT imaging experiments using an ^18^F-labeled version of the polymer to avoid the need for a bulky chelator ligand. The blood half-life of the polymer from the experiments with the ^89^Zr-labeled construct was also determined to be short enough to be compatible with the shorter half-life of F-18 (109.7 min). In these experiments, accumulation was not observed in key organs such as the liver, kidney, lungs, and spleen. We hypothesize that adding a large, hydrophobic radiometal-chelator probe to the polymer system altered the biodistribution or triggered the immune system. Regardless, the results were in accordance with the toxicity tests, demonstrating safety.

Next, experiments were undertaken to verify that the injection of GCG-conjugate (GCG-P2) at normal glucose levels did not induce hyperglycemia. The blood glucose levels were monitored for 8 h after injection of GCG micelle and remained above 125 mg/dL, well within the normoglycemia range. Surprisingly, glycemia levels of the mice treated with GCG micelle were lower than the PBS control by 30 mg/dL. This could be due to counterregulation effects and the release of endogenous insulin. Future experiments using a diabetic model would be needed to elucidate this hypothesis. Yet, the above experiments did show that the empty micelle was not causing any *in vivo* toxicity, and the GCG micelle was not inducing hyperglycemic conditions, indicating that the formulation was safe for further exploration. *In vivo* efficacy studies were performed in a healthy murine model, avoiding various variables associated with diabetes mouse models. According to experimental results, the GCG micelle was able to reverse deep hypoglycemic conditions in less than 25 min after injection, indicating that the glucagon formulation could be employed as an emergency treatment for hypoglycemia. Interestingly, when mice with moderate hypoglycemia were injected with GCG micelles, there was no hyperglycemic condition following this injection, further demonstrating the safety of the GCG micelle. Finally, when the CGC formulation was injected together with insulin, blood glucose levels were maintained in the normal range, whereas the control group experienced a drop in glycemia below the gluco-counterrgulatory threshold. This indicates that the GCG-micelle, acting in tandem with insulin, proved more effective at maintaining normoglycemia than the empty-micelle-insulin combination. In conclusion, these experimental results indicate that the GCG micelle could be used to avert the onset of insulin-induced deep hypoglycemia as a responsive system. Hypoglycemia occurs in diabetic patients 1–2 times per week on average for glucose levels below 70 mg/dL and approximately once a year for less than 50 mg/dL.^60^ Therefore, we envision that the glucagon in this formulation would be released once, or not all, before the material is excreted. As such, these studies highlight the potential for a glucagon formulation to manage glycemia by pre- or coadministration of glucagon-micelles with insulin.

## Conclusion

In conclusion, a novel glucose-responsive polymer-GCG conjugate system was developed that self-assembled into micelles under normoglycemic conditions *via* reversible binding of phenylboronic acid units to glucose and disassembled during hypoglycemia, releasing the active glucagon conjugate. The GCG-micelle exhibited a significant increase in activity compared to native glucagon *in vitro*. In mouse models of insulin-induced hypoglycemia, intraperitoneal administration of glucagon-micelles successfully regulated blood glucose concentration, both reverting and preventing deep hypoglycemia depending upon when the glucagon-micelles were injected relative to insulin administration. Importantly, *in vivo*, glucagon release from micelles only occurred at or below the counter-regulatory threshold, with no release observed under moderate hypoglycemia or normoglycemic conditions. Additionally, acute and chronic toxicity analyses *in vivo* along with μPET/μCT imaging demonstrated a favorable safety profile of this formulation, showing no organ accumulation. The proof-of-concept data indicates that the GCG-micelle is a promising candidate worthy of further investigation for the treatment of insulin-induced hypoglycemia.

## Materials and Methods

### Study Design

The objective of this study was to design and develop a glucose responsive delivery system for glucagon to combat severe hypoglycemia, either as an emergency medication or as a preventive measure. Two main goals were identified: I) to synthesize a polymer, fine-tuning its physicochemical characteristics, that serves as a carrier for glucagon and that is able to self-assemble into micelle and disassemble at the desired glucose levels, and II) to verify such formulation biological activity by testing for its potency *in vitro* and for its capacity to prevent or reverse severe hypoglycemia without side effects *in vivo*. First, we identified PEG-b-P(NIPAM-*stat*-2-APBA) as a polymer presenting all desired characteristics, such as the ability to self-assemble into micelles based on temperature and glucose response. We modified the polymer to be responsive in physiological conditions by altering the ratio of hydrophilic PEG block to responsive hydrophobic NIPAM-*stat*-2-APBA block as well as the percentage of phenyl boronic units present in the hydrophobic block. The polymer was modified to insert a disulfide to react with a thiolated glucagon, forming an active conjugate. Through careful fine-tuning of the length and ratios of each polymer block, the conjugate was engineered to respond to changes in glycemia at physiological range. After the above optimization, the conjugate activity was tested *in vitro* using cells overexpressing the glucagon receptor, finding an increased potency of the conjugate compared to native glucagon. Finally, the conjugate safety and activity were assessed *in vivo* using healthy male C57Bl/6J mice. To test the safety, animals were injected with either the conjugate or PBS as a control, and changes in their glycemia were monitored for 8 h. At the end of the study, kidney and liver tissues were collected to perform the histopathological analysis. Two experiments were performed to study the capacity of the micelle to reverse or prevent deep hypoglycemia. In the first, animals were fasted for 12 h and then injected with insulin to induce severe hypoglycemia. After, either the glucagon micelle or empty micelle as a control were administered monitoring changes in glycemia for 2 h. The second experiment was conducted similarly, but the glucagon micelle or empty micelle were administered at the same time as insulin. All animal procedures performed in this study were reviewed and approved by the UCLA Animal Oversite Committee and supervised by A.L.H. or R.M.VD.

### Statistical Analysis

*In vitro* and *in vivo* experimental values are reported as the mean ± SD and mean ± SEM, respectively. Graph Pad Prism 8 (GraphPad Software, San Diego, USA) was used for the statistical analyses. Two-way analysis of variance (ANOVA) followed by Bonferroni’s multiple comparison test was employed to compare the means and determine the significance of the differences. Results were considered significantly different if *p* < 0.05 (*); results are also reported with *p* < 0.01 (**), *p* < 0.001 (***) and *p* < 0.0001 (****).

## Data Availability

All data associated with this study are present in the paper or the Supporting Information.

## Supporting Information Available

Full synthetic and experimental details, characterization of thiolated GCG, polymers and nanomaterials, cloud point and DLS measurements, ThT assay, toxicity results, PET imaging characterization, and results not in the text (PDF)
