# Supplementary material for: A Glucose-Responsive Glucagon-Micelle for the Prevention of Hypoglycemia
Source: ACS Cent Sci. 2024 Oct 2;10(11):2036–47. doi: 10.1021/acscentsci.4c00937 (PMC11613347; doi:10.1021/acscentsci.4c00937)
Supplement: Supplementary file 1 — oc4c00937_si_001.pdf [file oc4c00937_si_001.pdf]

A Glucose-Responsive Glucagon-Micelle for the Prevention of Hypoglycemia

Daniele Vinciguerra,[1,2] Rajalakshmi P S, [1,2] Jane Yang,[1,2] Panagiotis G. Georgiou[1,2†]Katherine Snell[1†], Théo Pesenti,[1] Jeffrey Collins,[3] Mikayla Tamboline,[3] Shili Xu,[3,4] R. Michael van Dam,[3,4] Kathryn M. M. Messina,[1,2] Andrea L. Hevener,[5,6]*and Heather D. Maynard[1,2*]

1 Department of Chemistry and Biochemistry, University of California, Los Angeles, 607 Charles E. Young Drive East, Los Angeles, California 90095-1569, USA.

2 California NanoSystems Institute, University of California, Los Angeles, 570 Westwood Plaza, Los Angeles, California 90095-1569, USA.

3 Department of Molecular and Medical Pharmacology and Crump Institute for Molecular Imaging, David Geffen School of Medicine, University of California, Los Angeles, California 90095-1735, USA.

4 Jonsson Comprehensive Cancer Center, David Geffen School of Medicine, University of California, Los Angeles, California 90095-1735, USA.

5 Department of Medicine, Division of Endocrinology, David Geffen School of Medicine at UCLA, 650 Charles E. Young Dr., Los Angeles, CA 90095, USA.

6 VA Greater Los Angeles Healthcare System GRECC, Los Angeles, California USA 90073

† These authors contributed equally to this work.

*Corresponding Authors: maynard@chem.ucla.edu (H.D.M.) ; ahevener@mednet.ucla.edu (A.L.H.)

Materials and methods

Materials

Solvents were purchased as ACS grade and used without any further purification and anhydrous solvents used were freshly distilled. 2-(((Ethylthio)carbonothioyl)thio)-2-methylpropanoic acid chain transfer agent was synthesized following a protocol from literature.[1] Sterile dulbecco's phosphate-buffered saline (DPBS), 2,2'-dithiodipyridine (98%, DTP), *N*,*N*′-dicyclohexylcarbodiimide (99%, DCC), 4-(dimethylamino)pyridine (≥99%, DMAP), polyethylene glycol methyl ether (PEG, M_n ~ 2,000 g.mol^{-1}), 2-aminophenylboronic acid hydrochloride (≥95%), *N*-(3-dimethylaminopropyl)-*N*′-ethylcarbodiimide hydrochloride (≥98%, EDC), 4-fluorobenzoic acid (98%), *N*-hydroxysuccinimide (98%), *N*-(2-aminoethyl)maleimide trifluoroacetate salt (≥98%), *N*,*N*-diisopropylethylamine (≥99%), 1,4-dithiothreitol (DTT, 97%), tris-(2-Carboxyethyl)phosphine hydrochloride (TCEP) (≥ 97%), and ethanolamine (98%) were purchased from Sigma Aldrich and Fisher Scientific and used without further purification. Chemicals of 2,2′-azobis(2-methylpropionitrile) (98%, AIBN), and *N*-isopropylacrylamide (97%, NIPAM) were purchased from Sigma Aldrich and recrystallized from acetone and hexane, respectively. Acrylic acid (99%, AAc) was also purchased from Sigma Aldrich and purified by distillation. Activity assay kits of aspartate aminotransferase (AST), alanine aminotransferase (ALT), lactate dehydrogenase (LDH) and calcium-detecting assay kit were also purchased from Sigma Aldrich. Thiolated glucagon (GCG-SH, sequence: HSQGTFTSDYSKYLDSRRAQDFV***C***WLMNT) and native GCG (sequence:

HSQGTFTSDYSKYLDSRRAQDFV***Q***WLMNT) were purchased from Biomatik at >90% purity. For the glucagon activity assay, commercial kit cAMP Hunter™ eXpress GCGR CHO-K1 GPCR assay was purchased from Eurofins DiscoverX Products, LLC. ^{89}Zr-oxalate was obtained from 3D Imaging LLC. Contrast agent Fenestra HDVC CT was purchased from MediLumine. Vaporized isoflurane was obtained from McKesson.

Characterization techniques

NMR Spectroscopy. ^{1}H-NMR spectra were recorded at 400 MHz or 500 MHz on an Avance spectrometer respectively, with chloroform-*d* ($CDCl_3$), DMSO-d_6 ($(CD_3)_2SO$) and deuterium oxide (D_2O) as the solvent. Chemical shifts of protons are reported as δ in parts per million (ppm) and are relative to tetramethyl silane (TMS) at $\delta = 0$ ppm when using $CDCl_3$ or solvent residual peak ($(CD_3)_2SO$), $\delta = 2.50$ ppm/ D_2O, $\delta = 2.79$ ppm).

Dynamic Light Scattering. Hydrodynamic diameters (D_h) and size distributions of micellar formulations were determined by dynamic light scattering (DLS) at a polymer concentration of 10 mg/mL in DPBS supplemented with the adequate glucose concentration using a Malvern Zetasizer Nano ZS with a 4 mW He-Ne 633 nm laser module. Measurements were carried out at an angle of 173° (back scattering), and results were analyzed using Malvern DTS 7.03 software. All determinations were repeated 3 times with at least 10 measurements recorded for each run.

Reverse-Phase High Performance Liquid Chromatography (HPLC). HPLCs of polymer-glucagon conjugates were carried out on an Agilent 1260 Infinity II HPLC system equipped with an autosampler and a UV detector using a Zorbax 300SB-C18 (analytical: 3.5 µm, 3.0 × 150 mm) with monitoring at $\lambda = 220$ nm and with a flow rate of 0.8 mL/min, using a gradient 25-95% of solvent B (A: H_2O + 0.1% TFA; B: ACN + 0.1% TFA) over 17 min.

Electrospray Ionization Mass Spectroscopy (ESI-MS). ESI-MS was performed using an Agilent 6530 ESI-Q-TOF in tandem with a 1260 Infinity LC.

Turbidimetry. Turbidimetric analysis was performed on a SpectraMax iD3 spectrophotometer plate reader. Aqueous polymer solutions were prepared at 10 mg.mL^{-1} in DPBS with changes in transmittance monitored at $\lambda = 600$ nm by heating each sample from 20 °C to 65 °C at a rate of 1 °C min^{-1}. Lower critical solution temperature (LCST) for each thermal phase transition curve was set for abs = 0.5 .

Transmission Electron Microscopy (TEM). Dry-state-stained TEM imaging was performed on a FEI T12 instrument microscope operating at an acceleration voltage of 120 kV. All dry-state samples were deposited onto formvar-coated carbon grids. Grids were glow discharged for 15 seconds. Samples (5 mg/mL) were incubated either at 25 °C or 40 °C for 30 min. After roughly 5 min, excess sample was blotted from the grid and the grid was stained with an aqueous 2 wt% uranyl acetate (UA) solution for 1 min prior to blotting, drying and microscopic analysis.

SDS-PAGE. SDS-PAGE gels, samples were loaded using 2X Laemmli sample buffer and run on Mini-Protean TGX, Any kD gels (Bio-Rad) at 195V for 35 min using Tris/Glycine/SDS buffer (Bio-Rad). Gels were stained with Coomassie. SDS-PAGE protein standards were obtained from Bio-Rad (Precision Plus Protein Prestained Standards).

Circular Dichroism. Circular dichroism (CD) analysis of thiolated and native glucagon samples was performed on a Jasco J-1715 CD spectropolarimeter, featuring a 150 W air-cooled Xe lamp and fitted with a temperature controlling system. The sample was prepared at 0.25 mg/mL concentration, in a mixture of DPBS (pH = 7.4)/HCl (pH = 3) (1/1) and contained in a 1mm pathlength quartz cuvette (Hellma, USA) with a spectral bandwidth of 1.0 nm.

Thioflavin T (ThT) assay. BSA fibrils prepared at 1 mg mL^{-1} were used as the positive control by inducing fibrils by heating to 80 °C for 1 h.* GCG-micelles were incubated with 100 equiv. TCEP for 15 min at 4 °C before analysis. The fibrillation was evaluated for fresh GCG-micelle, LCST cycle 1 and LCST cycle 2. 250 µL of ThT solution at 50 µM (in DPBS) was added into a black plate followed by the addition of 50 µL of either GCG-micelle, or BSA. The

solutions were covered in the dark and incubated at 25 °C for 20 min. Fluorescence intensity was then measured using a plate reader (*excitation wavelength* = 450 nm, *emission wavelength* = 482 nm). *Note: BSA was used instead of GCG as a control because the ThT fluorescence is dependent on both acidic and basic pH, exhibiting a significant decrease in ThT absorbance. Therefore, since GCG is only soluble in either acidic or basic pH and not at the neutral pH of this experiment, it was not suitable to be used as a control.

Synthetic Procedures

Synthesis of PEG-macromolecular chain transfer agent (mCTA).

In a round bottom flask, the chain transfer agent 2-[[(ethylthio)carbonothioyl]thio]-2-methylpropanoic acid (168 mg, 0.750 mmol, 1.5 eq), DCC (206 mg, 1.00 mmol, 2 eq), and DMAP (122 mg, 1.00 mmol, 2 eq) were dissolved in 4 mL of dry DCM under Ar(g). Polyethylene glycol methyl ether (M_n ~2000 Da) (1000 mg, 0.500 mmol, 1 eq) was dissolved in 1 mL of dry DCM and added dropwise to the mixture, which was then allowed to stir for 72 h at 25 °C. The reaction mixture was then filtered to remove solid urea byproduct. The solvent was evaporated under reduced pressure and the product was purified by flash chromatography (SiO_2, gradient DCM to DCM/MeOH 8/2 as eluent) to obtain of a yellow solid as the final product (0.782 g, yield= 70.3 %). ^{1}H NMR (400 MHz, $CDCl_3$) δ (ppm) = 4.27 – 4.22 (m, 2H), 3.70 – 3.57 (m, 178H), 3.37 (s, 3H), 3.27 (q, J = 7.4 Hz, 2H), 1.67 (d, J = 9.8 Hz, 6H), 1.31 (t, J = 7.4 Hz, 3H) (Figure S1).

Representative synthesis of PEG-b-P(NIPAM-stat-AAc).

A concentrated solution of AIBN (1.02 mg, 0.006 mmol) in dry DMSO was prepared and the desired amount was added to a Schlenk flask containing PEG macro-CTA (54.9 mg, 0.025 mmol), AAc (33.7 mg, 0.468 mmol), and NIPAM (300 mg, 2.65 mmol). Theoretical feed ratio for NIPAM:AAc was 85:15 in every case. Total monomer to initiator ratios were varied to target different molecular weights. Dry DMSO was added to reach 3 mL and 3 freeze pump/thaw cycles were performed. The flask was filled with Ar(g) and stirred at 75 °C for 2h. Polymerization was quenched by exposing to air and an aliquot of the crude mixture was taken for ^{1}H NMR analysis. NIPAM conversion was calculated by comparing the areas under the peak of monomeric and polymeric amide peak at δ = 7.9 ppm and 6.9-7.6 ppm, respectively. AAc mol% was calculated by comparing the area under the peak of PAAc polymeric acidic peak at 11.8-12.1 ppm and PNIPAM polymeric amide peak at 6.9-7.6 ppm. DP_{PNIPAM} was calculated by comparing PEG glycolic peak at 3.4-3.5 ppm and NIPAM polymeric amide peak at 6.9-7.6 ppm. DP_{PAAc} was calculated by comparing PEG glycolic peak at 3.4-3.5 ppm and AAc polymeric acidic peak at 11.8-12.1 ppm. Procedure was repeated for PEG/NIPAM/AAc ratios of 1/57/10 (P1), 1/106/19 (P2), 1/212/38 (P3), 1/283/50 (P4), 1/472/83 (P5) (see Table S1). *Note: Multiple polymer batches of P2 were synthesized across this study with near identical molecular weights 15,00 ± 0,5 kDa.*

$M_{n,NMR}$ was calculated according to the formula $M_{n,NMR} = M_{n,\ PEG\ mCTA} + (DP_{PNIPAM} \times M_{W\ NIPAM}) + (DP_{PAAc} \times M_{W\ AAc})$. The crude mixture was directly used in the following reaction. ^{1}H NMR (400 MHz, DMSO-d_6) δ (ppm) = 11.96 (s, 22H), 7.16 (s, 110H), 3.85 (m, 121H), 3.47 (s, 178H), 1.96 (m, 154H), 1.76 – 1.16 (m, 246H), 1.12 – 0.75 (m, 717H) (Figure S2).

Representative synthesis of PEG-b-P(NIPAM-stat-2-APBA).

Crude PEG-*b*-P(NIPAM-*stat*-AAc) (AAc eq, 0.468 mmol) solution in DMSO, was added to 10 mL of MilliQ water at 0 °C in a round bottom flask and stirred for 1 h until polymer dissolution. EDC (360 mg, 1.88 mmol) and DMAP (36.5 mg, 0.234 mmol) were then added, and the solution was stirred at 25 °C for 20 min. 2-APBA.HCl (159 mg, 0.938 mmol) was added, and the mixture stirred for 16 h at 25 °C. Solution was dialyzed against DI water for 3 days (MWCO = 3.5 kDa) and lyophilized to obtain a white solid with quantitative conversion. 2-APBA mol% was calculated by comparing the area under the peak of 2-APBA aromatic peak at δ = 6.8-7.5 ppm and NIPAM tertiary isopropyl peak at δ = 3.6-3.9 ppm. DP_{PNIPAM} was calculated by comparing PEG glycolic peak at δ = 3.5 ppm and

NIPAM tertiary isopropyl peak at 3.6-3.9 ppm. $DP_{P(2\text{-}APBA)}$ was calculated by comparing PEG glycolic peak at δ =3.5 ppm and 2-APBA aromatic peaks at δ = 6.8-7.5 ppm.

$M_{n,\,NMR}$ was calculated according to the formula $M_{n,\,NMR} = M_{n,\,PEG\,mCTA} + (DP_{PNIPAM} \times M_{W,\,NIPAM}) + (DP_{P2\text{-}APBA} * M_{W,\,2\text{-}APBA})$. ^{1}H NMR (400 MHz, D_2O) δ (ppm) = 7.20 (t, 83H), 3.74 (s, 80H), 3.57 (s, 178H), 2.18 – 1.74 (m, 118H), 1.43 (s, 187H), 1.00 (s, 553H). ^{13}C NMR (126 MHz, D_2O) δ 175.13, 172.70, 135.52, 131.60, 128.26, 127.38, 116.12, 70.96, 69.56, 69.41, 58.03, 42.72, 41.74, 34.60, 21.55, 21.38.

End-group modification of PEG-b-P(NIPAM-stat-2-APBA) with pyridyl disulfide (PDS).

PEG-*b*-P(NIPAM-*stat*-2-APBA) (M_n = 15.0 kDa, 100 mg, 0.0067 mmol) and DTP (29.0 mg, 0.133 mmol) were dissolved in 1 mL of dry methanol. The solution was sparged for 20 min with Ar(g). Ethanolamine (1.63 mg, 0.027 mmol) was added directly by syringe. The solution was stirred under Ar(g) for 3 h at 25 °C. The polymer was first dialyzed (MWCO = 3.5 kDa) against an acetone/water mixture (50/50 v/v %) for 16 h to remove the excess of DTP, and then dialyzed against water for another 2 days to remove acetone. Polymer solution was lyophilized to obtain a white solid. Functionalization was calculated by comparing the signal from PDS group at δ = 8.2-8.3 ppm with signal from PEG at δ = 3.5-3.6 ppm. All polymers showed quantitative functionalization.

Conjugation of glucagon (GCG-SH) with PEG-b-P(NIPAM-stat-2-APBA)-PDS.

PEG-*b*-P(NIPAM-*stat*-APBA)-PDS (M_n = 15 kDa, 5.1 mg, 0.343 µmol) was dissolved in 0.5 mL of DPBS (pH =7.4) and added to a solution of GCG-SH (1 mg, 0.286 µmol) in 0.5 mL HCl (10 mM, pH = 2). The solution was stirred for 4 h at 4 °C. Crude, conjugated mixture was purified by centrifugal filtration (MWCO = 10 kDa, 3x HCl 10 mM, 3x DPBS pH = 7.4, 10 min, 13200 rpm, 4 °C). Reaction was monitored by analytical HPLC, showing maximum conversion of 83.0% within 4 h.

End-group modification of PEG-b-P(NIPAM-stat-2-APBA) (P2/P3) with deferoxamine-maleimide (DFO-mal).

PEG-*b*-P(NIPAM-*stat*-2-APBA)-PDS (M_n = 15.0 kDa, 20 mg, 0.0013 mmol) was dissolved in 1 mL of a 10 mM tris(2-carboxyethyl)phosphine hydrochloride (TCEP) aqueous solution. The mixture was further purified by centrifugal filtration to remove free pyridyl disulfide (MWCO = 3 kDa, 3×TCEP, 10 min, 13200 rpm, 4 °C). Thiol-terminated polymer was recovered and diluted in 1 mL of MilliQ water. Triethylamine (TEA, 0.0013 mmol, 1 mg) and deferoxamine-maleimide (DFO-mal) (0.00421 mmol, 3 mg) were added and the mixture was stirred at room temperature for 16 h. Crude polymer was further purified by centrifugal filtration (MWCO =10 kDa, 10× DPBS, 10 min, 13200 rpm, 4 °C) and lyophilized to obtain a white solid. ^{1}H NMR analysis confirmed successful functionalization (Figure S14).

^{89}Zr-radiolabeling of P2 and P3.

^{89}Zr-radiolabeling of the P2 and P3 were adapted from Tavaré *et al.*[2] with slight modifications. Chemicals and materials were purchased from Sigma Aldrich (St. Louis, MO) except when indicated. [^{89}Zr]Zr-oxalate was obtained from 3D Imaging LLC. Upon arrival, [^{89}Zr]Zr-oxalate was diluted with 40% (v/v) 2 M Na_2CO_3 and allowed to incubate for 3 min. The activity was then diluted with 2.5× volume of 1 M HEPES (pH = 7.0). Final pH was checked with pHydrion plastic indicator strips (Micro Essential Laboratory) to confirm a pH of 7. The DFO-conjugated micelle or linear polymer was added to the buffered [^{89}Zr]Zr-oxalate to give a molar activity of 180–220 MBq/mg and mixed for 1 h at 37 °C using a thermomixer with shaking (600–800 rpm). Radiolabeling efficiency was measured by ITLC (Biodex Medical Systems) using 20 mM citrate buffer pH = 5.6 as the mobile phase using a Wizard 3″ 1480 Automatic Gamma Counter (Perkin-Elmer). Radiolabeled non-micelle and micelle were purified using a Centriprep filter with a 10 kDa MWCO. Radiochemical purity was assessed by ITLC as above. The counts at the origin (O) and at the solvent front (SF) were used to calculate % labeling efficiency or % radiochemical purity by the equation: O/(O + SF) × 100%. For dose calibration measurements, a Capintec CRC-55tR (Capintec, Ramsey NJ) calibrated for Zr-89 was utilized (Calibration # 465). A final molar activity of 0.16–0.2 MBq/µg [^{89}Zr]Zr-DFO-micelle/non-micelle was used in the experiments.

^{18}F-FBEM labeling of micelle.

The synthesis of ^{18}F-FBEM was adapted from Collins *et al.*[3] A purified sample containing 159 MBq of dried ^{18}F-FBEM dissolved in 50 µL of DMSO was used for labeling the micelle. 1.5 mg of micelle (1 equivalent, 112 nmol) was first dissolved in 500 µL of MilliQ water in a dram vial. 0.16 mg of TCEP (5 equivalents, 560. nmol) was then added to the solution and allowed to stir for 10 min. Following the stirring, 500 µL of 1M borate buffer (pH = 8) was added and stirred for 2 min. The ^{18}F-FBEM in 50 µL of DMSO was then pipetted into the micelle solution and allowed to react for 25 min at room temperature with stirring. TCEP and unreacted ^{18}F-FBEM were removed using a Centriprep filter with a 3 kDa MWCO. A final molar activity of 141 MBq/µg was produced for the imaging experiments.

^{18}F-SFB labeling of GCG-SH.

The synthesis of ^{18}F-SFB was adapted from Lazari *et al.*[4] A purified sample containing 330 MBq of dried ^{18}F-SFB in 50 µL of DMSO was used for labeling the glucagon. 0.5 mg (1 equivalent, 140 nmol) of glucagon-SH was dissolved in 500 µL of 10 mM HCl in a LoBind Eppendorf tube. Then 500 µL of pH = 8 borate buffer (1M) was added to the solution. The reaction mixture was allowed to react 5-30 min. The ^{18}F-SFB in 50 µL of DMSO was then pipetted into the glucagon solution and allowed to react for 25 min at room temperature with stirring. The unreacted ^{18}F-SFB was removed using a Centriprep filter with a 3 kDa MWCO. A final molar activity of 141 MBq/µg was produced for the imaging experiments.

Methods

In vitro glucagon activity assay.

Commercial kit cAMP Hunter™ eXpress GCGR CHO-K1 GPCR assay was purchased from Eurofins and used following manufacturer protocol. In brief, cells were plated according to the manufacturer's protocol for 96-well plate assay (1 vial of cells/96-well plate, 30000 cells/well). Cells were incubated for 18 h at 37 °C, and 5% CO_2 atmosphere. Cell plating buffer was then removed and replaced with 30 µL of cell assay buffer. 15 µL of agonist solution was added to appropriate wells and plates were incubated at 37 °C for 30 min. After, 15 µL of antibody Solution and 60 µL of cAMP working detection solution were added to the cells, and plates were incubated for 1 h at 25 °C in the dark. Finally, 60 µL of cAMP solution A was added and plates were incubated for 24 h at 25 °C in the dark, before reading the chemiluminescent signal. A four-parameter logistic fit was applied to the results to obtain EC_{50} values using GraphPad Prism 7.0. The mean and standard error of the mean of five to six independent repeats were used for calculations.

In vivo acute and chronic toxicity of PEG-b-P(NIPAM-stat-2-APBA) (P2).

In vivo acute and chronic toxicity of P2 was evaluated in C57Bl/6J mice (11 weeks, male). The acute toxicity was evaluated for 0 h, 24 h and 120 h. The mice were given a single dose of 2.322 mg/kg of empty micelles (P2) injected intraperitoneally and were sacrificed at different intervals to determine the whole blood count (CBC) (n = 4 or 5) and organ weight (n = 6).

To evaluate chronic toxicity, mice were administered with 2.32 mg/kg of empty micelle (P2) daily by intraperitoneal injection continuously for 14 days. Mice administered with saline were the control group for the chronic toxicity. After 14 days of injection, the mice were sacrificed to determine the body weight, CBC count, organ weight, histopathology, hepatic function parameters, kidney function parameters, immune markers, and immunohistochemistry. The blood was collected and stored in EDTA-coated tubes for CBC analysis (n=5). The remaining blood was centrifuged, and the serum was collected and stored at -80 °C for further analysis of other

parameters. Urine was collected and stored in -80 °C. The CBC was done by IDEXX BioAnalytics. All organs were incubated in formalin for 18 h at 4 °C and then transferred/stored in 70% ethanol until taken for sectioning and staining with hematoxylin and eosin (H&E) (n = 5) by the UCLA Translational Pathology Core Laboratory. The hepatic function parameters kidney function parameters were evaluated from the blood serum using an aspartate aminotransferase activity assay kit (AST), alanine aminotransferase activity assay kit (ALT) and a lactate dehydrogenase activity assay kit (LDH) (n = 8). The kidney function parameters were evaluated using a calcium-detecting assay kit from urine (n = 4) and blood glucose (n = 10) levels using the Hemocue glucometer. The immune markers IFN γ, TNF α and IL 2 (n = 10) were analyzed using the Meso Scale Discovery (MSD) assay kits and MSD software. The immunohistochemistry for the lung and liver tissue was done using organ-specific immune marker F8/40 antibody at the UCLA Translational Pathology Core Laboratory.

Micro-Positron Emission Tomography (μPET)/ Micro-Computed Tomography (μCT).

μPET/μCT for ^{89}Zr-micelle polymer, ^{89}Zr-non-micelle polymer, ^{18}F-FBEM micelle polymer and ^{18}F-SFB glucagon was performed on a GNEXT PET/CT scanner (Sofie Biosciences, Dulles, VA). Upon injection, an initial μPET scan (energy window 350-650 keV) was performed followed by a 1-min μCT scan (voltage 80kVP, current 150 μA, 720 projections, 200 μm resolution) for all samples.

μPET/μCT ^{89}Zr-labeled micelle /^{89}Zr-labeled linear polymer.

Eight C57BL/6 mice (7 weeks, male, Jackson Laboratory) were anesthetized with 1.5% vaporized isoflurane and were injected *via* i.v. injections (tail vein) with 100uL CT contrast agent Fenestra HDVC agent (MediLumine), followed by another i.v. injection of 25-30 μCi radiolabeled *^{89}Zr*-labeled micelle (n = 4) or radiolabeled *^{89}Zr*-labeled linear polymer (n = 4). Each mouse was scanned immediately after injection for a 1 h dynamic μPET scan (energy window 350-650 keV) followed by a 1-min μCT scan (voltage 80kVP, current 150 μA, 720 projections, 200μm resolution) on a GNEXT PET/CT scanner (Sofie Biosciences, Dulles, VA). Mice were imaged by μPET/μCT again at 2h, 6h, 24h, 48h, 96h, and 192h (static μPET, 10-60 minutes), post tracer injection. The μPET images were reconstructed using the 3D-OSEM/MAP algorithm (24 subsets and 3 iterates) with random, attenuation, and decay correction. The μCT images were reconstructed using a Modified Feldkamp Algorithm. Amide software was used to analyze co-registered μPET /μCT images.

μPET/μCT ^{18}F-FBEM-labeled micelle/ /^{18}F-SFB-labeled glucagon.

Eight C57BL/6 mice (7 weeks, male, Jackson Laboratory) were anesthetized with 1.5% vaporized isoflurane and were injected via i.v. injection (tail vein) with 100 μL CT contrast agent Fenestra HDVC agent (MediLumine), followed by another i.v. injection of 70-75 μCi radiolabeled ^{18}F-FBEM micelle polymer (n = 4) or radiolabeled ^{18}F-SFB glucagon non-micelle polymer (n = 4). Each mouse was scanned immediately after injection for a 1-h dynamic μPET scan (energy window 350-650 keV) followed by a 1-min μCT scan (voltage 80kVP, current 150 μA, 720 projections, 200μm resolution) on a GNEXT PET/CT scanner (Sofie Biosciences, Dulles, VA). Mice were imaged by μPET/μCT again at 2h, 4h, 6h, and 8h (static μPET, 10-90 min), post tracer injection. The μPET images were reconstructed using the 3D-OSEM/MAP algorithm (24 subsets and 3 iterates) with random, attenuation, and decay correction. The μCT images were reconstructed using a Modified Feldkamp Algorithm. Amide software was used to analyze co-registered μPET /μCT images.

In vivo safety evaluation of PEG-b-P(NIPAM-stat-2-APBA)-GCG.

C57Bl/6J mice (11 weeks, male, n = 5-6, Charles River Laboratories) were fasted for 4 h before being injected intraperitoneally with either 200 μL of a solution of P2-GCG in DPBS (pH = 7.4) at a dose of 500 μg/Kg or 200 μL of DPBS (pH = 7.4) alone. Blood glucose level was assessed at predetermined time points (0, 15, 30, 45, 60, 480 min) using an Hemocue glucometer. At the end of the study, mice were sacrificed, and liver and kidneys were harvested for histopathological analysis. Organs were fixed in formalin for 36 h at 25 °C, and then transferred in ethanol 70%

and kept at 4 °C until taken for sectioning and staining with hematoxylin and eosin (H&E) by the UCLA Translational Pathology Core Laboratory.

In vivo hypoglycemia reversal.

C57Bl/6J mice (11 weeks, male, n = 5-6, Charles River Laboratories) were fasted for 12 h and then injected intraperitoneally with either 0.90 U/kg of insulin or 0.85 U/kg of insulin, to induce deep or moderate hypoglycemia, respectively. After 60 min, mice were injected with either 200 µL of a solution of P2-GCG in DPBS (pH = 7.4) at a dose of 500 µg/kg or 200 µL of P2 in DPBS (pH = 7.4) at polymer equivalent dose or native and thiolated glucagon at a dose of 500 µg/Kg. Blood glucose levels were assessed every 15 min for 2h using a Hemocue glucometer.

In vivo hypoglycemia prevention.

C57Bl/6J mice (11 weeks, male, n = 5-6, Charles River Laboratories) were fasted for 12 h and then injected intraperitoneally with 200 µL of a solution containing both 0.90 U/kg of insulin and either 200 µL of a solution of P2-GCG in DPBS at a dose of 500 µg/kg or 200 µL of P2 in DPBS at polymer equivalent dose. Blood glucose level was assessed every 15 min for 2h using a Hemocue glucometer.

Supplementary Figures

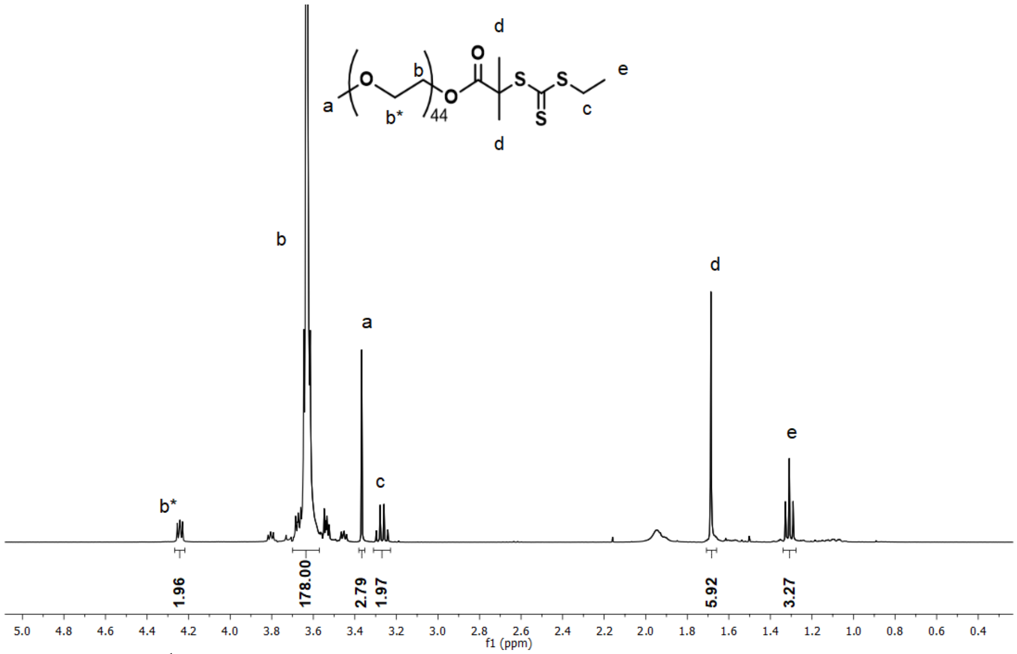

Figure S1. Representative ^{1}H-NMR spectrum of PEG macro-chain transfer agent (CTA) recorded in $CDCl_3$ (400 MHz).

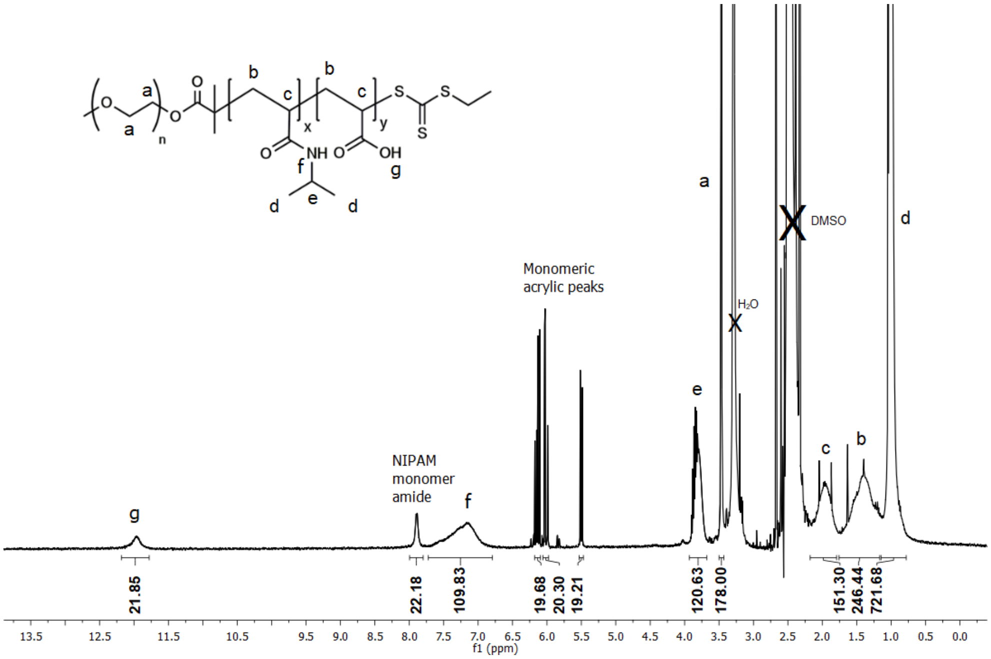

Figure S2. Representative ^{1}H NMR spectrum of crude PEG-*b*-P(NIPAM-*stat*-AAc) block copolymer precursor P2 recorded in DMSO-d_6 (400 MHz).

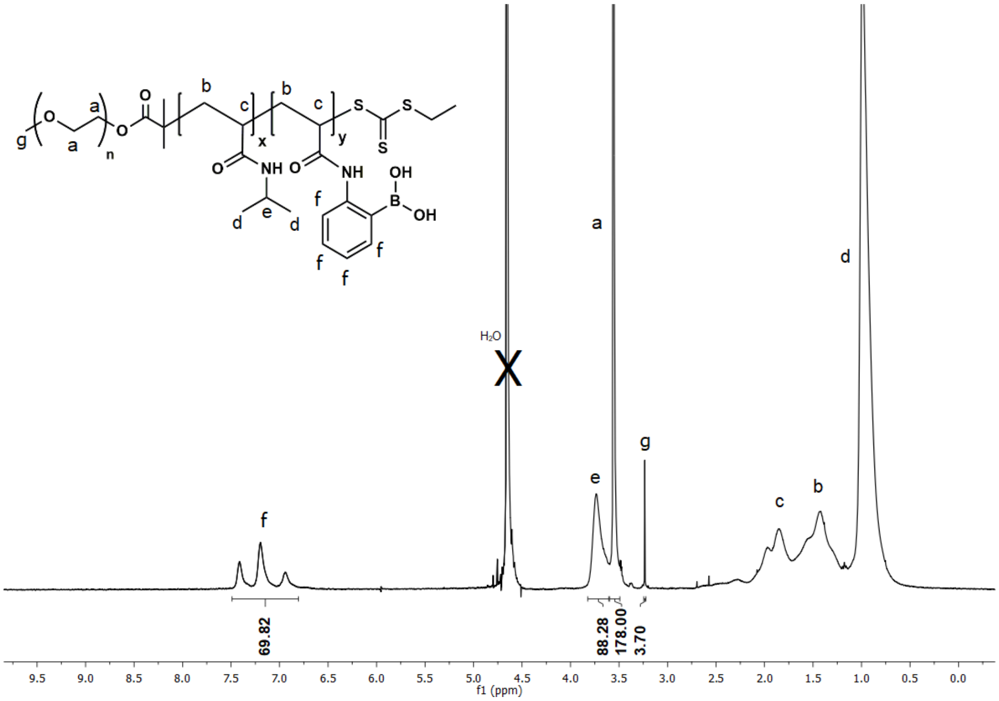

Figure S3. Representative ^{1}H NMR spectrum of PEG-*b*-P(NIPAM-*stat*-2-APBA) block copolymer P2 recorded in D_2O (400 MHz).

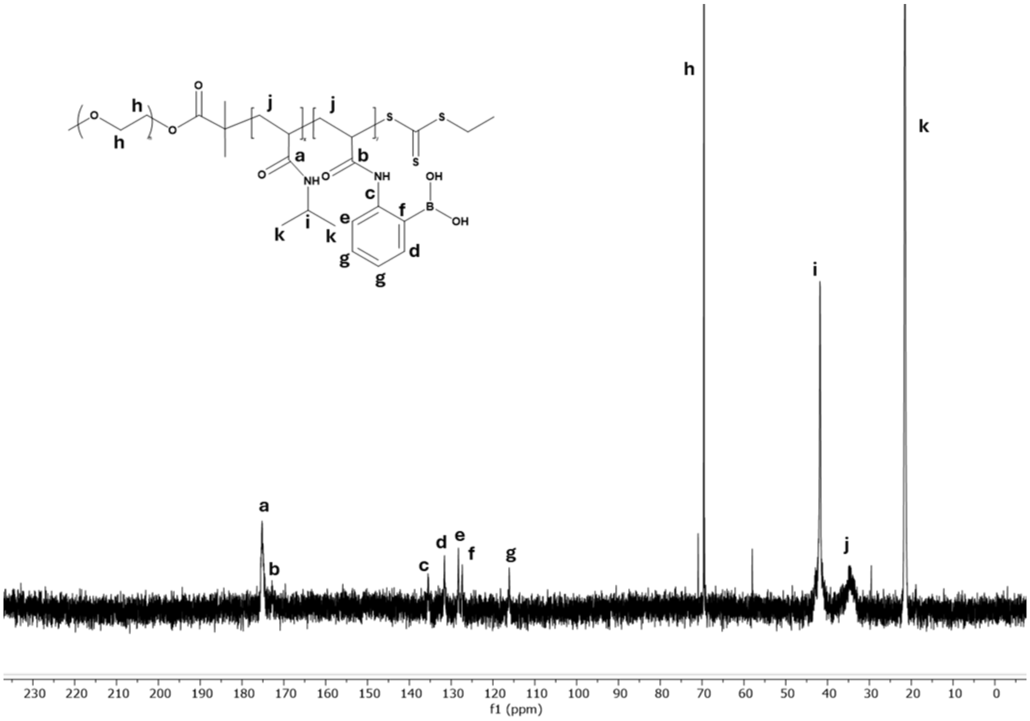

Figure S4. Representative ^{13}C NMR spectrum of PEG-*b*-P(NIPAM-*stat*-2-APBA) block copolymer P2

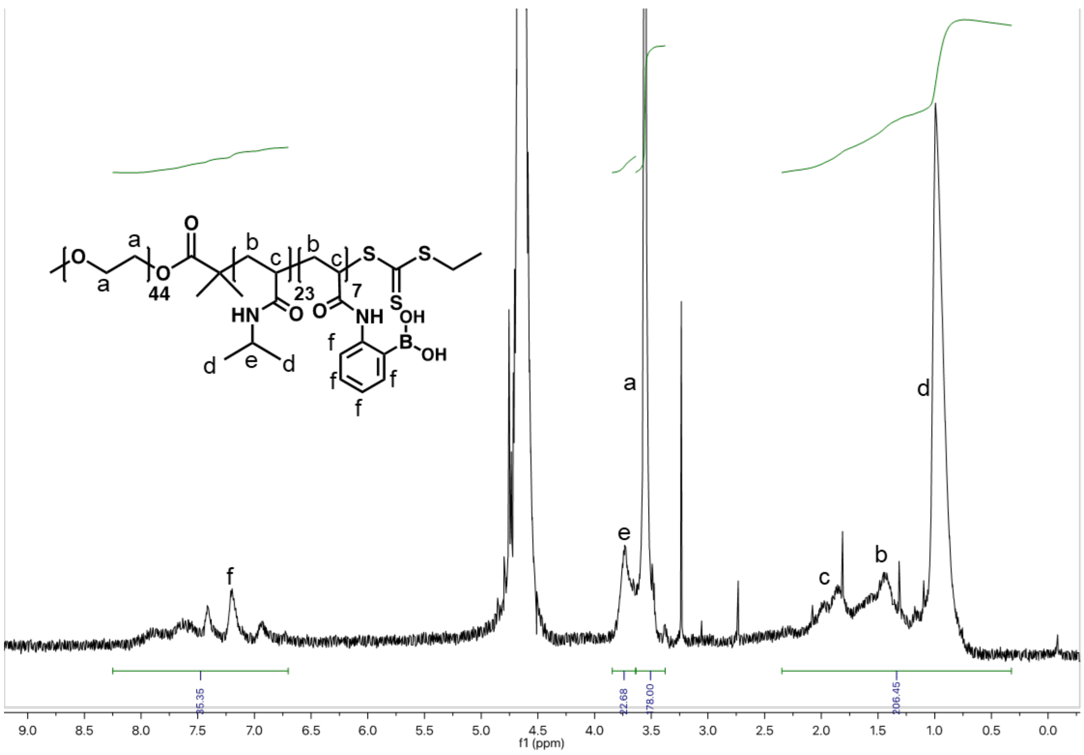

Figure S5. Representative ^{1}H NMR spectrum of PEG-*b*-P(NIPAM-*stat*-2-APBA) block copolymer P1 recorded in D_2O (400 MHz).

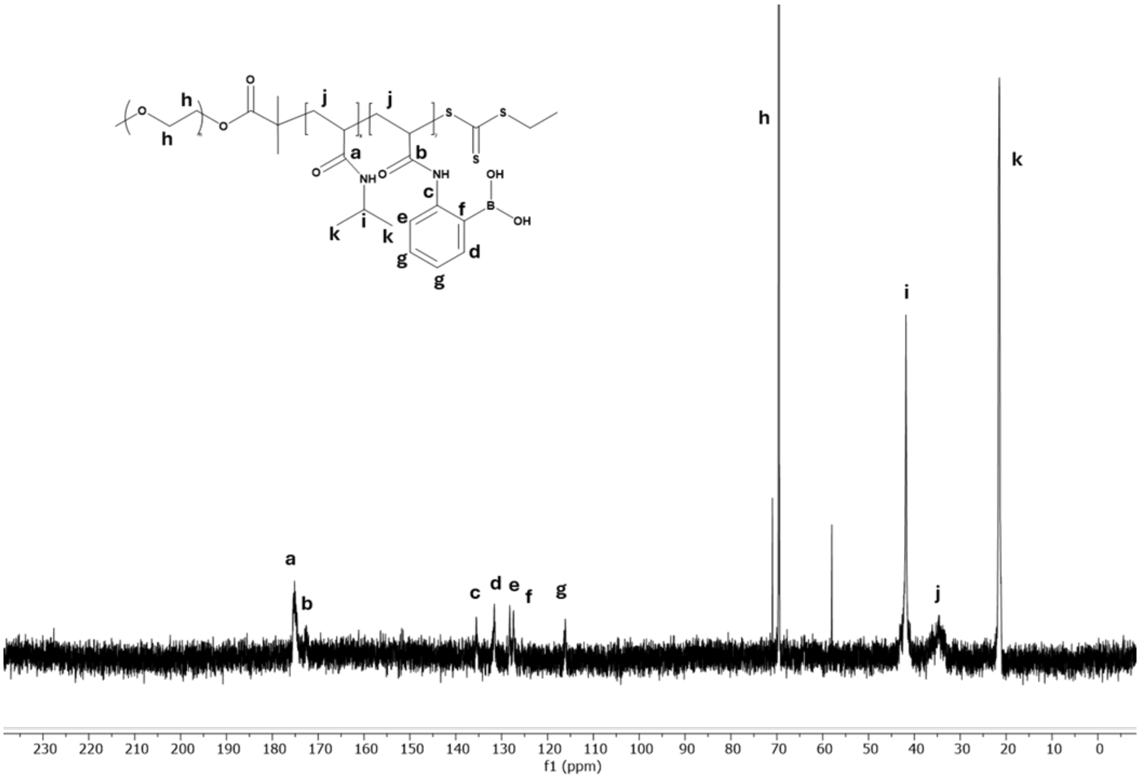

Figure S6. Representative ^{13}C NMR spectrum of PEG-*b*-P(NIPAM-*stat*-2-APBA) block copolymer P1

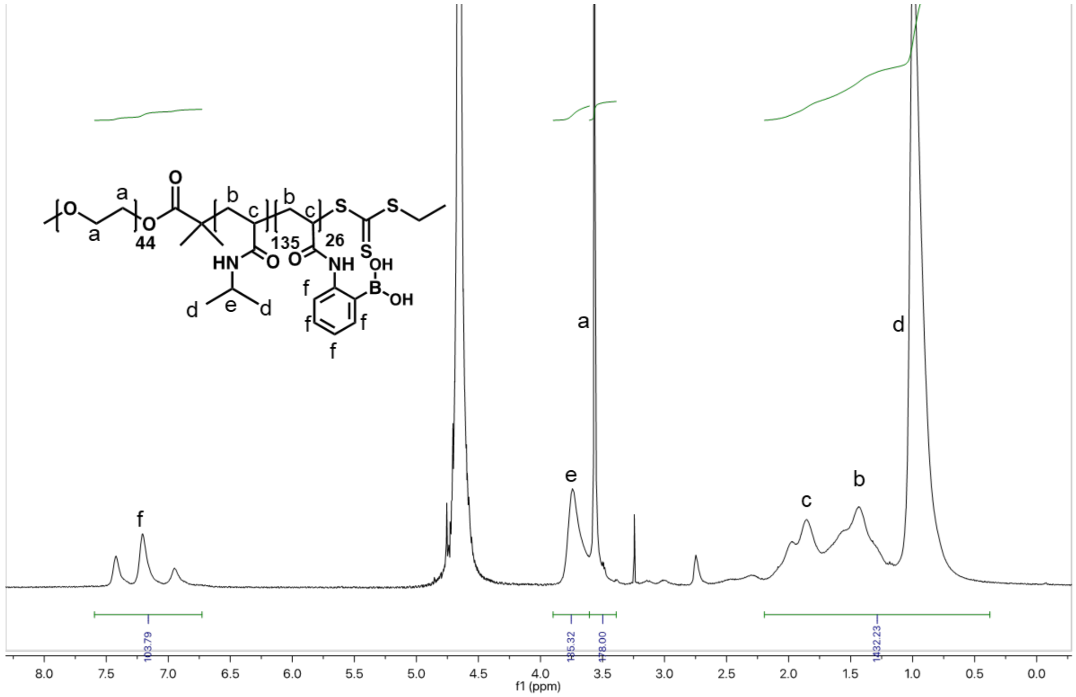

Figure S7. Representative ^{1}H NMR spectrum of PEG-*b*-P(NIPAM-*stat*-2-APBA) block copolymer P3 recorded in D_2O (400 MHz).

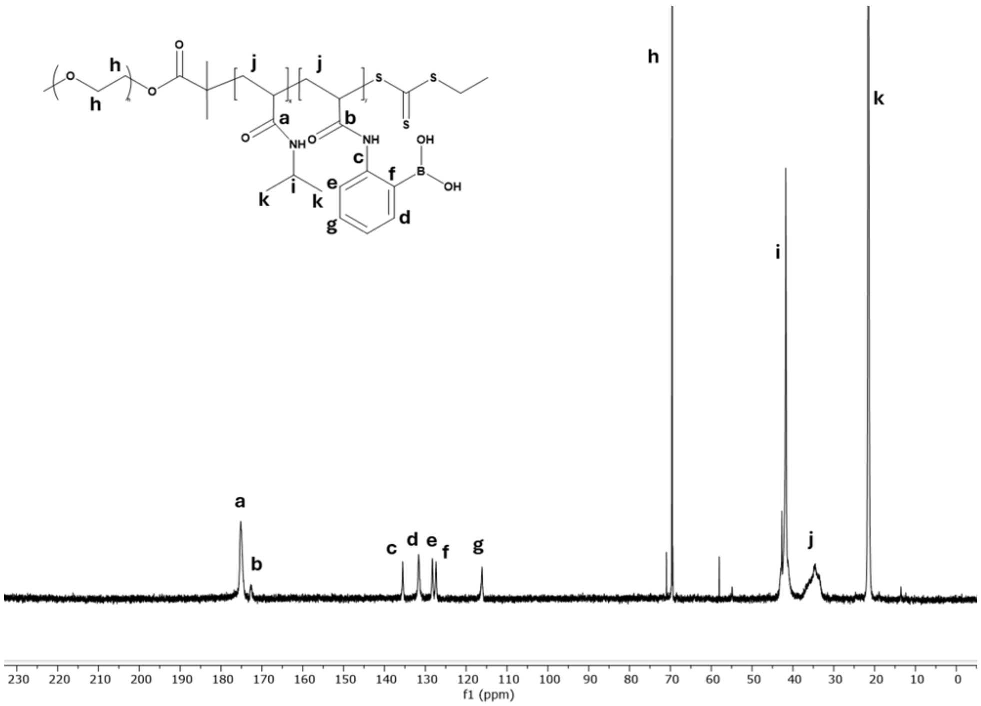

Figure S8. Representative ^{13}C NMR spectrum of PEG-*b*-P(NIPAM-*stat*-2-APBA) block copolymer P3

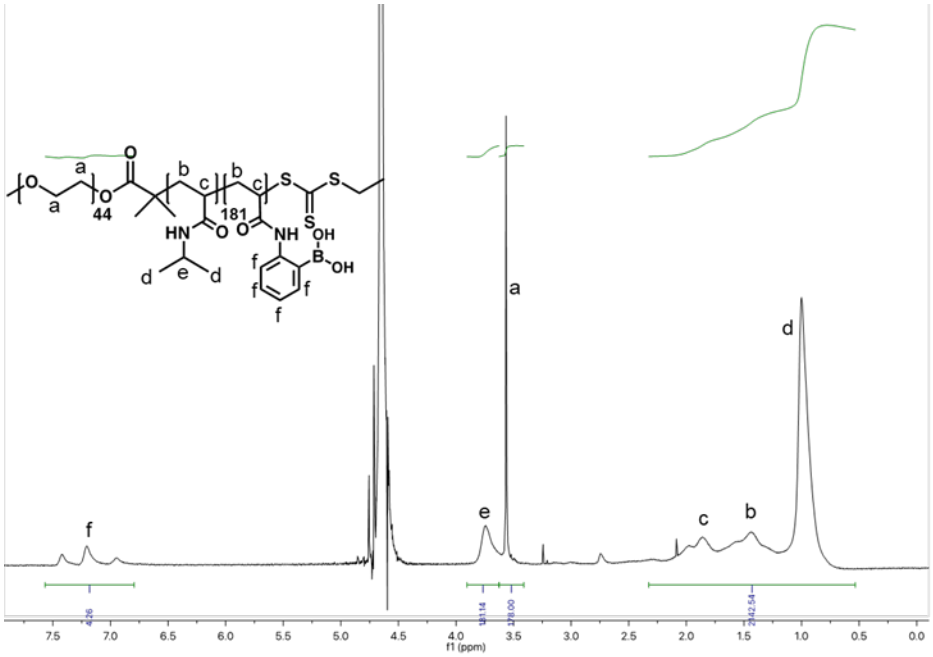

Figure S9. Representative ^{1}H NMR spectrum of PEG-*b*-P(NIPAM-*stat*-2-APBA) block copolymer P4 recorded in D_2O (400 MHz).

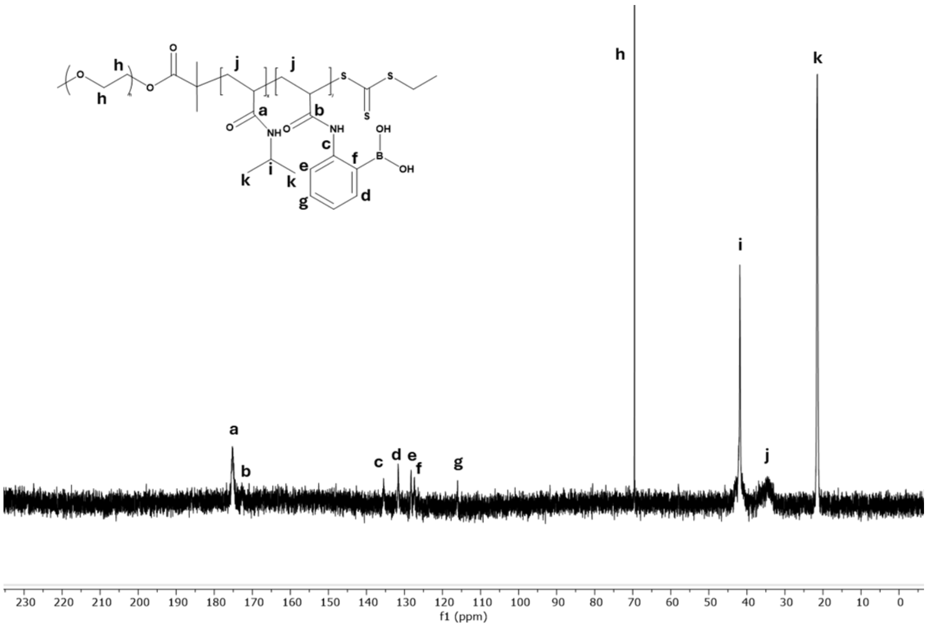

Figure S10. Representative ^{13}C NMR spectrum of PEG-*b*-P(NIPAM-*stat*-2-APBA) block copolymer P4

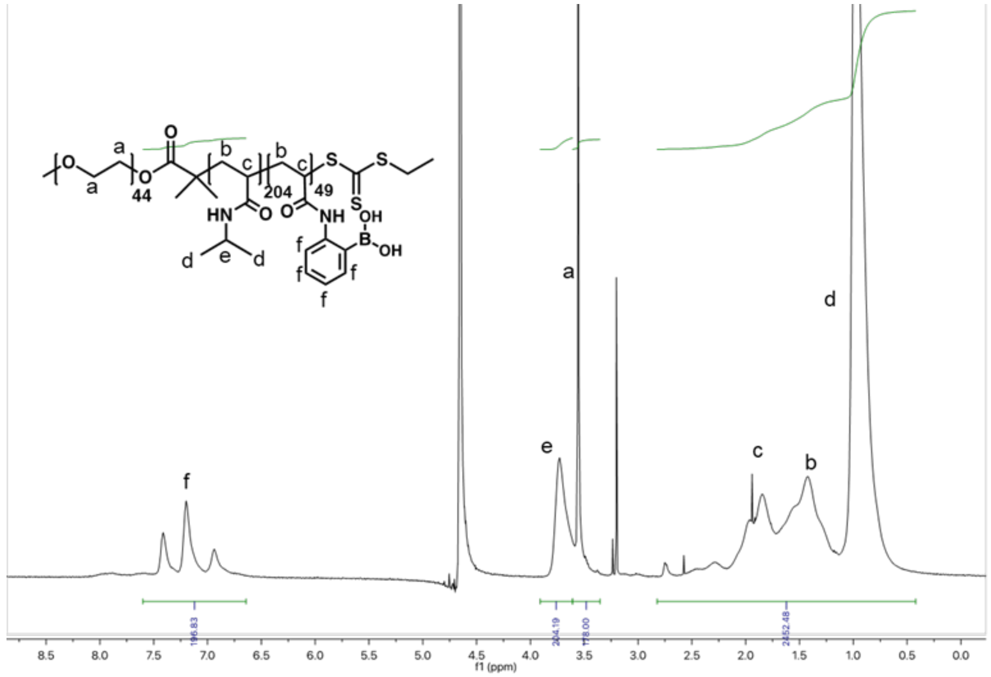

Figure S11. Representative ^{1}H NMR spectrum of PEG-*b*-P(NIPAM-*stat*-2-APBA) block copolymer P5 recorded in D_2O (400 MHz).

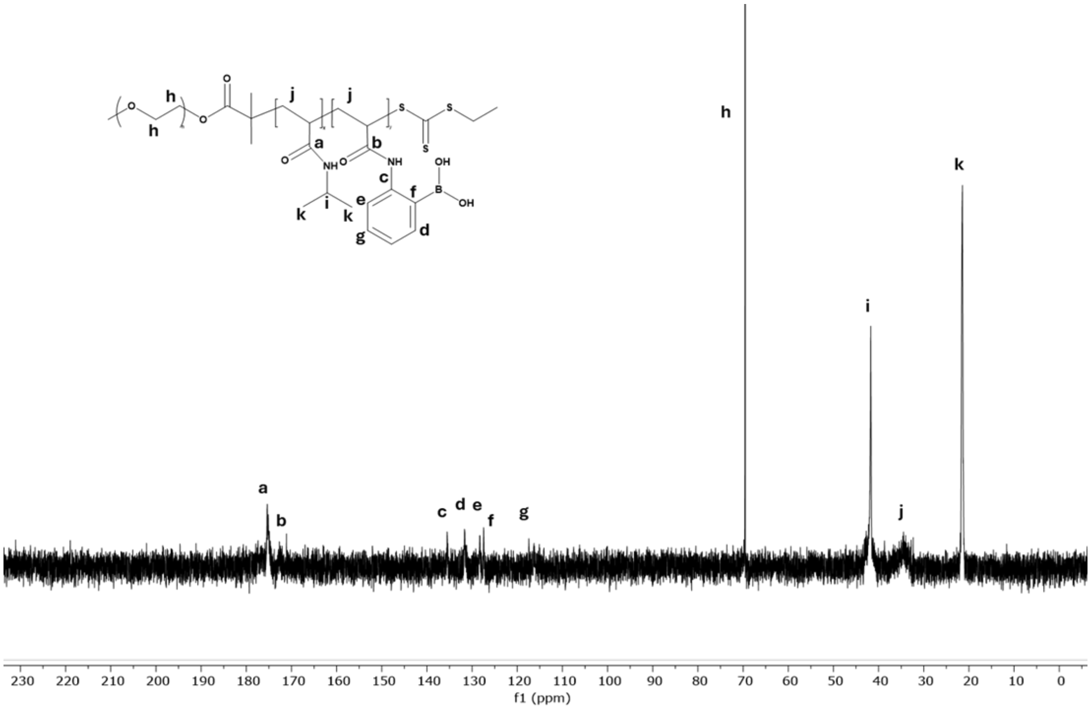

Figure S12. Representative ^{13}C NMR spectrum of PEG-*b*-P(NIPAM-*stat*-2-APBA) block copolymer P5

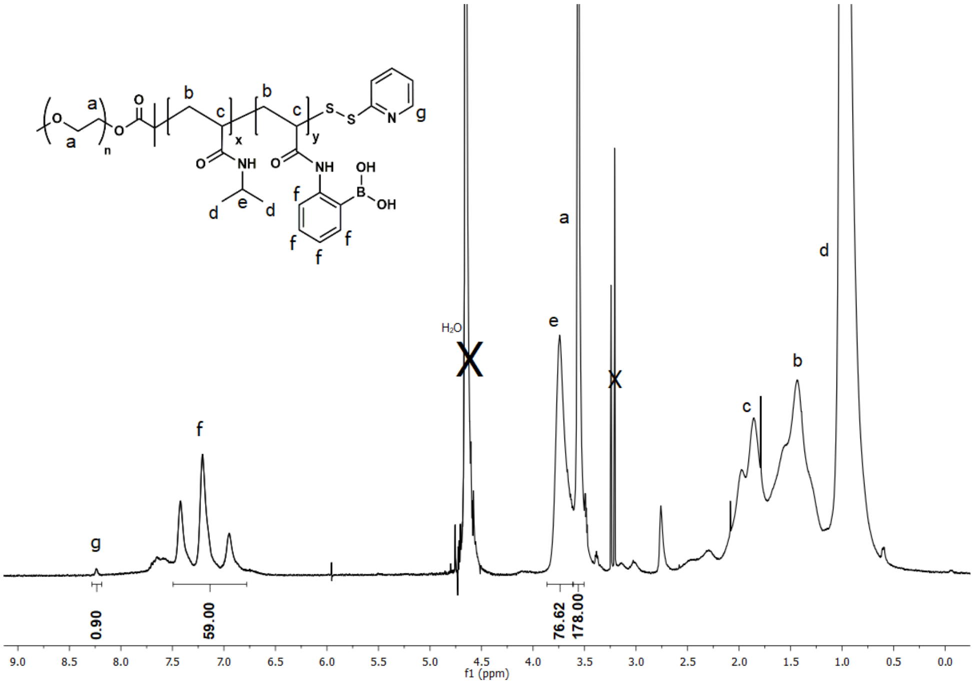

Figure S13. Representative ^{1}H NMR spectrum of end-group functionalized PEG-*b*-P(NIPAM-*stat*-2-APBA)-PDS recorded in D_2O (400 MHz).

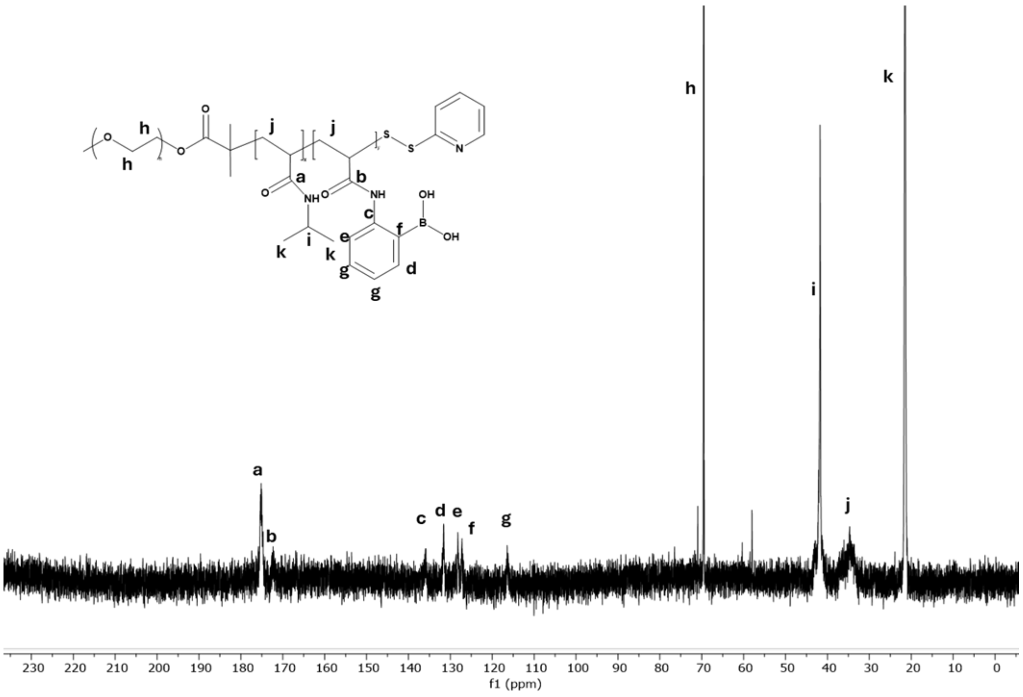

Figure S14. Representative ^{13}C NMR spectrum of end-group functionalized PEG-*b*-P(NIPAM-*stat*-2-APBA)-PD

Table S1. Block copolymer characteristics of PEG-*b*-P(NIPAM-*stat*-2-APBA) library.

Sample	$M_{n,\,NMR}$ (kDa)[a]	2-APBA mol%[a]	Cloud point (°C)[b]	D_h (nm)[c]	PDI[c]
**P1**	10.0	20.7	63.3	n.d.	n.d.
**P2**	15.0	17.7	62.1	40.67	0.133
**P3**	22.6	16.6	47.7	47.21	0.242
**P4**	36.1	18.4	46.3	46.75	0.136
**P5**	48.0	18.3	n.d.	44.51	0.171

[a] Determined by ^{1}H NMR analysis in D_2O. [b] Determined by UV-Vis spectroscopy by measuring Abs at λ = 600 nm using 10 mg/mL of polymer. [c] Average D_h and PDI values (from 3 repeat measurements) obtained by dynamic light scattering (DLS) at 40 °C (P1-P4) or 37 ºC (P5).

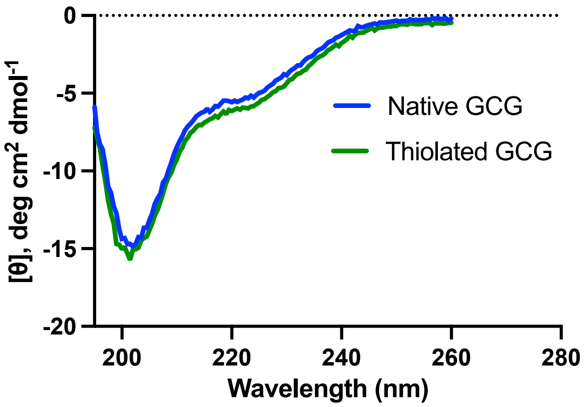

Figure S15. Circular dichroism (CD) spectra of native and thiolated glucagon (GCG) recorded at 0. 125 mg/mL using a mixture of DPBS (pH = 7.4)/HCl (pH = 3) (1/1).

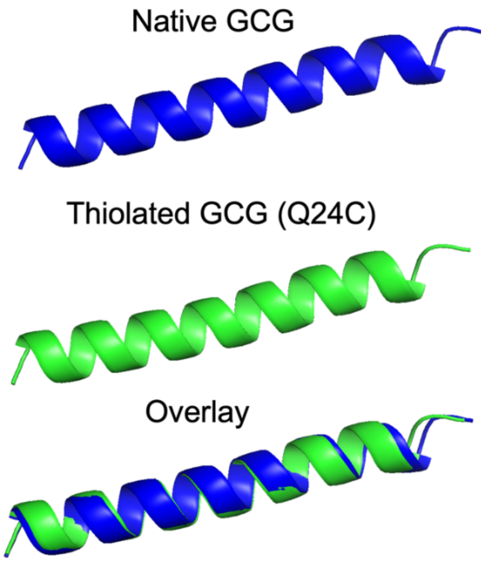

Figure S16. Predicted 3D structure of native and thiolated glucagon by AlphaFold.[5,6]

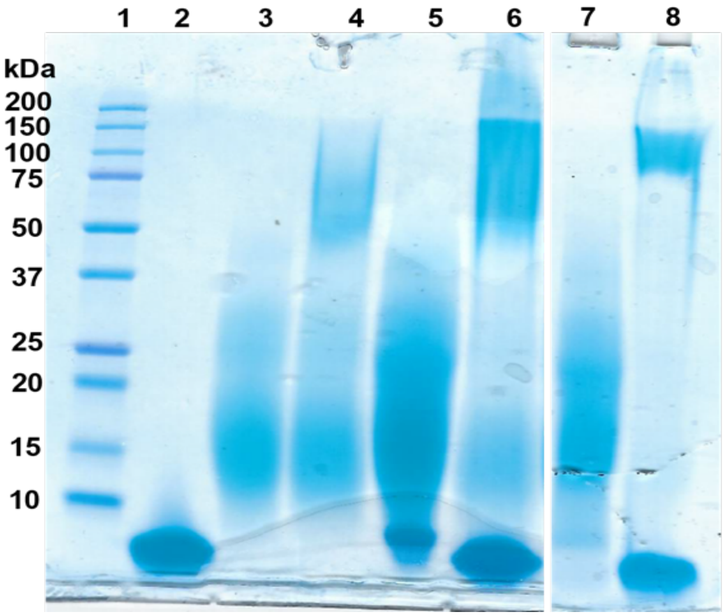

Figure S17. SDS-PAGE. Lane 1: protein ladder; lane 2: GCG-SH, lane 3: PEG-*b*-P(NIPAM-*stat*-2-APBA) (P2); lane 4: PEG-*b*-P(NIPAM-*stat*-2-APBA) reduced with dithiothreitol (DTT); lane 5: crude conjugation reaction; lane 6: crude conjugation reaction reduced with DTT; lane 7: pure conjugate PEG-*b*-P(NIPAM-*stat*-2-APBA)-GCG (P2-GCG); lane 8: pure conjugate PEG-*b*-P(NIPAM-*stat*-2-APBA)-GCG (P2-GCG) reduced with DTT.

Lane 2 shows GCG-SH below the 10 kDa ladder band, in agreement with GCG-SH molecular weight. Lane 3 and 4 show PEG-*b*-P(NIPAM-*stat*-2-APBA)-GCG (P2-GCG) before and after treatment with dithiothreitol DTT, respectively. The polymer appears as a smear in the 10-30 kDa range and reduction with DTT lead to some higher molecular weight species. No low MW bands appear, as expected. Lane 5 contains the crude conjugation reaction. A small amount of GCG-SH compared to polymer can be seen, in agreement with HPLC data at 4h. The polymer smear intensity increased, which is indicative of the presence of GCG in the conjugate. Upon reduction with DTT in lane 6, the low molecular weight band assigned to GCG-SH reappears with higher intensity. Finally, lane 7 contains the pure conjugate as noted by the absence of free unbonded GCG-SH. Again, reduction with DTT in lane 8 releases GCG-SH, as can be noted by the appearance of the band assigned to GCG-SH.

Table S2. PEG-*b*- P(NIPAM-*stat*-APBA)-GCG conjugate library characteristics

**Conjugated polymer**	**Total M_w (kDa)**[a]	**Cloud point (°C)**[b]	**D_h (nm)**[c]	**PDI**[c]
**P1-GCG**	13.5	n.d.	38.45	0.489
**P2-GCG**	18.5	50.6	50.39	0.280
**P3-GCG**	26.1	57.2	67.04	0.246
**P4-GCG**	39.6	43.8	119.6	0.122

[a]Calculated by the addition of precursor polymer $M_{n\ (NMR)}$ + $M_{n\ (GCG\text{-}SH)}$. [b] Determined by UV-Vis spectroscopy, absorbance recorded at λ = 600 nm. [c] Average D_h and PDI values (from 3 repeat measurements) obtained by dynamic light scattering (DLS) at 37 °C with the addition of [Glc] = 150 mg/dL.

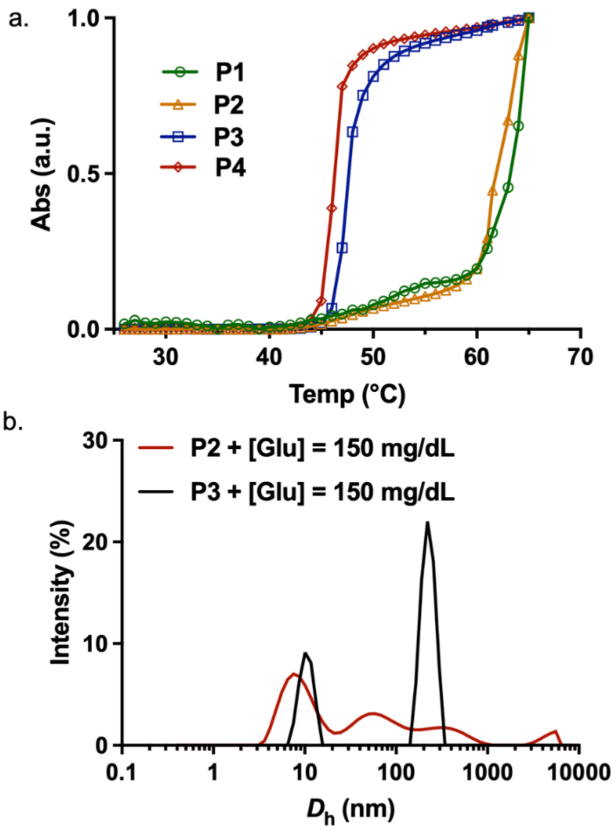

Figure S18. (a) Normalized cloud points of P1-P4 (10 mg/mL in DPBS) measured by UV-vis spectroscopy in the 26-65 °C range by determining absorbance at 600 nm. (b) Intensity-weighted size distributions obtained by DLS of P2 and P3 at 37 °C in the presence of [Glc] = 150 mg/dL indicating no formation of well-defined micelles.

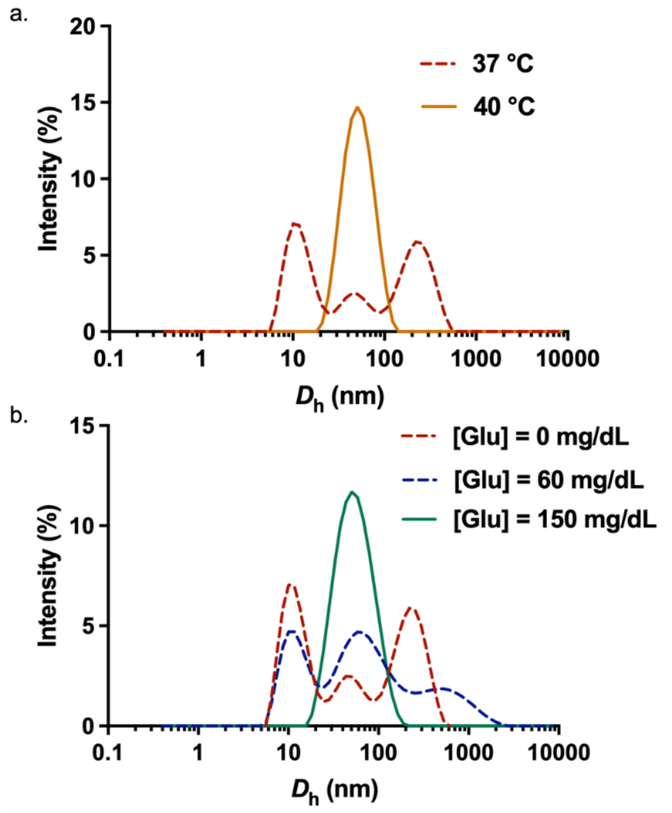

Figure S19. (a) Intensity-weighted curves of P4 at 37 and 40 °C obtained by DLS, showing micelle formation at 40 °C. (b) Intensity-weighted curves of P4 at 37 °C, showing micelle formation at normoglycemia ([Glc] = 150 mg/dL).

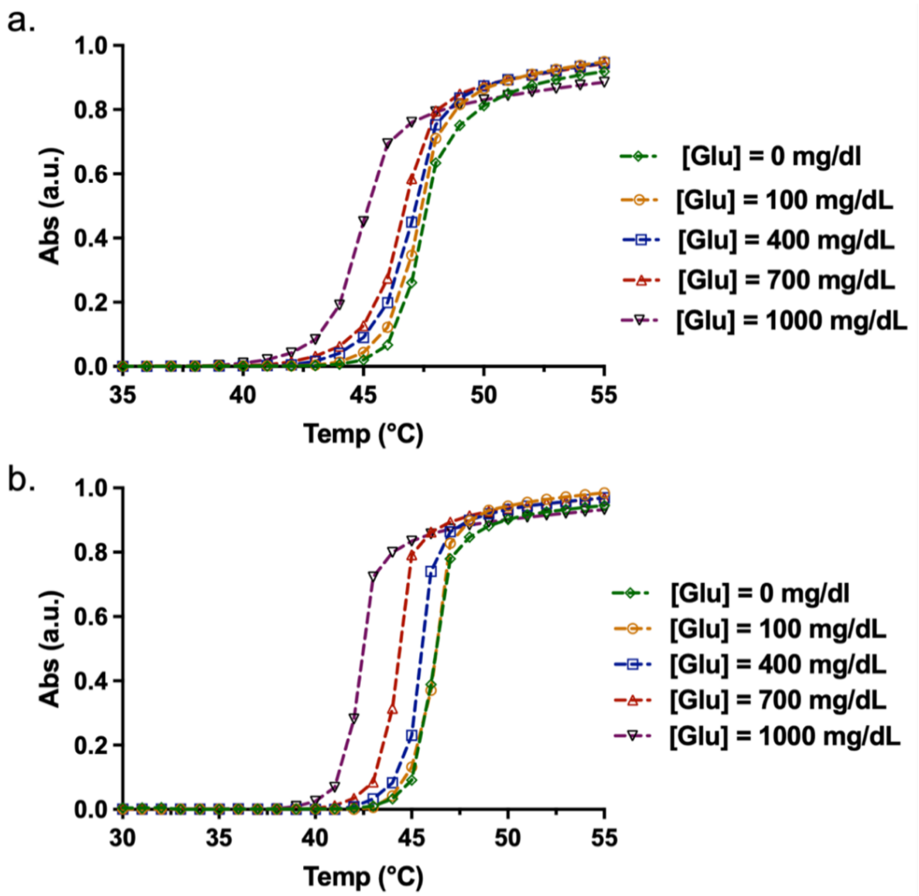

Figure S20. Normalized cloud points of a) P3 and b) P4 (10 mg/mL in DPBS) with [Glc] = 0, 100, 400, 700 and 1000 mg/dL measured by UV-Vis spectroscopy in the range of 30-55 °C by determining absorbance at λ = 600 nm.

A minor discrepancy between the data obtained from UV-vis spectroscopy and DLS was noted. However, UV-vis measures the cloud point, thus the results are dependent on the polymer concentration. On the other hand, DLS data is independent of the polymer concentration and determines the temperature at which micelles self-assemble, rather than the cloudiness/optical density of the solution, which results in higher sensitivity.

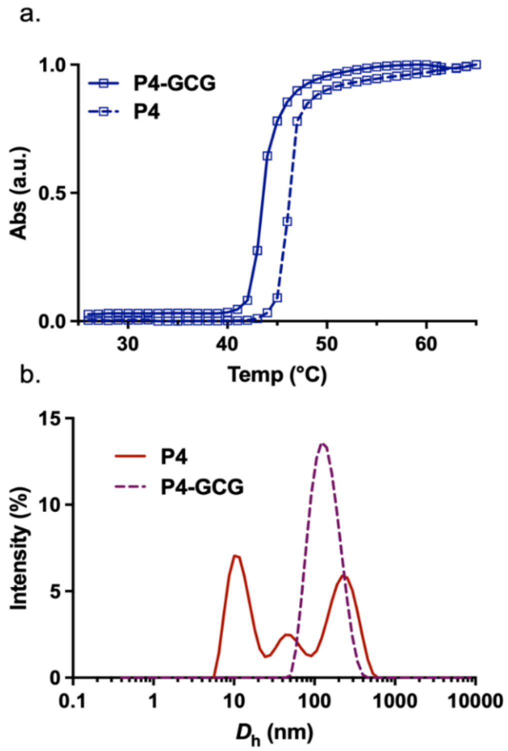

Figure S21. a) Normalized cloud point of P4 and P4-GCG (10 mg/mL in DPBS) measured by UV-Vis spectroscopy in the range of 26-62 °C by determining absorbance at λ = 600 nm. b) Intensity-weighted size distributions obtained by DLS for P4 and P4-GCG at 37 °C, without glucose addition.

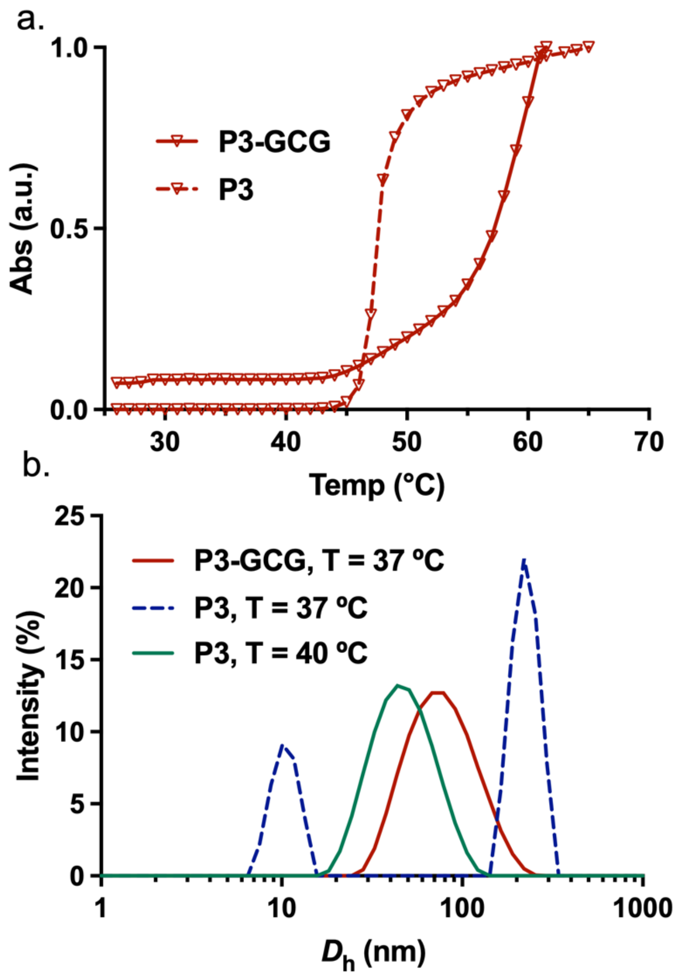

Figure S22. a) Normalized cloud point of P3 and P3-GCG (10 mg/mL in DPBS) measured by UV-vis spectroscopy in the 26-65 °C range by determining absorbance at λ = 600 nm. b) Intensity-weighted DLS curves of P3-GCG at 37 °C and P3 at 37 and 40 °C.

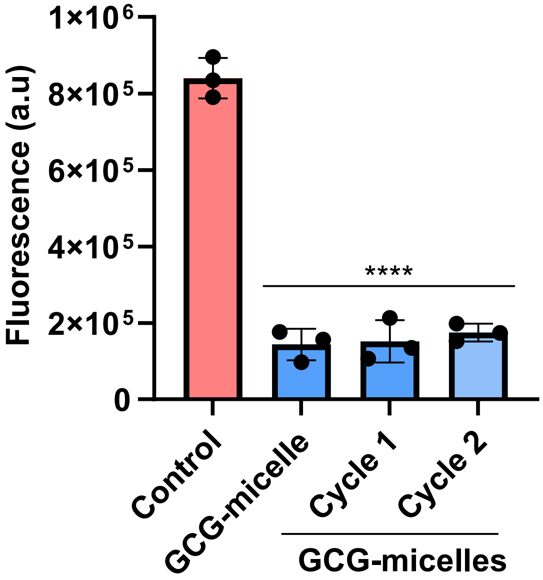

Figure S23. Glucagon–SH fibrillation was assessed by measuring the ThT fluorescence of GCG-micelles after conjugation, one and two heating above the LCST cycles. The control for the is BSA heated at 80 °C for 1 h. Statistical significance was determined *via* a one-way ANOVA with multiple comparisons (****$p \leq 0.0001$.) ($n = 6$).

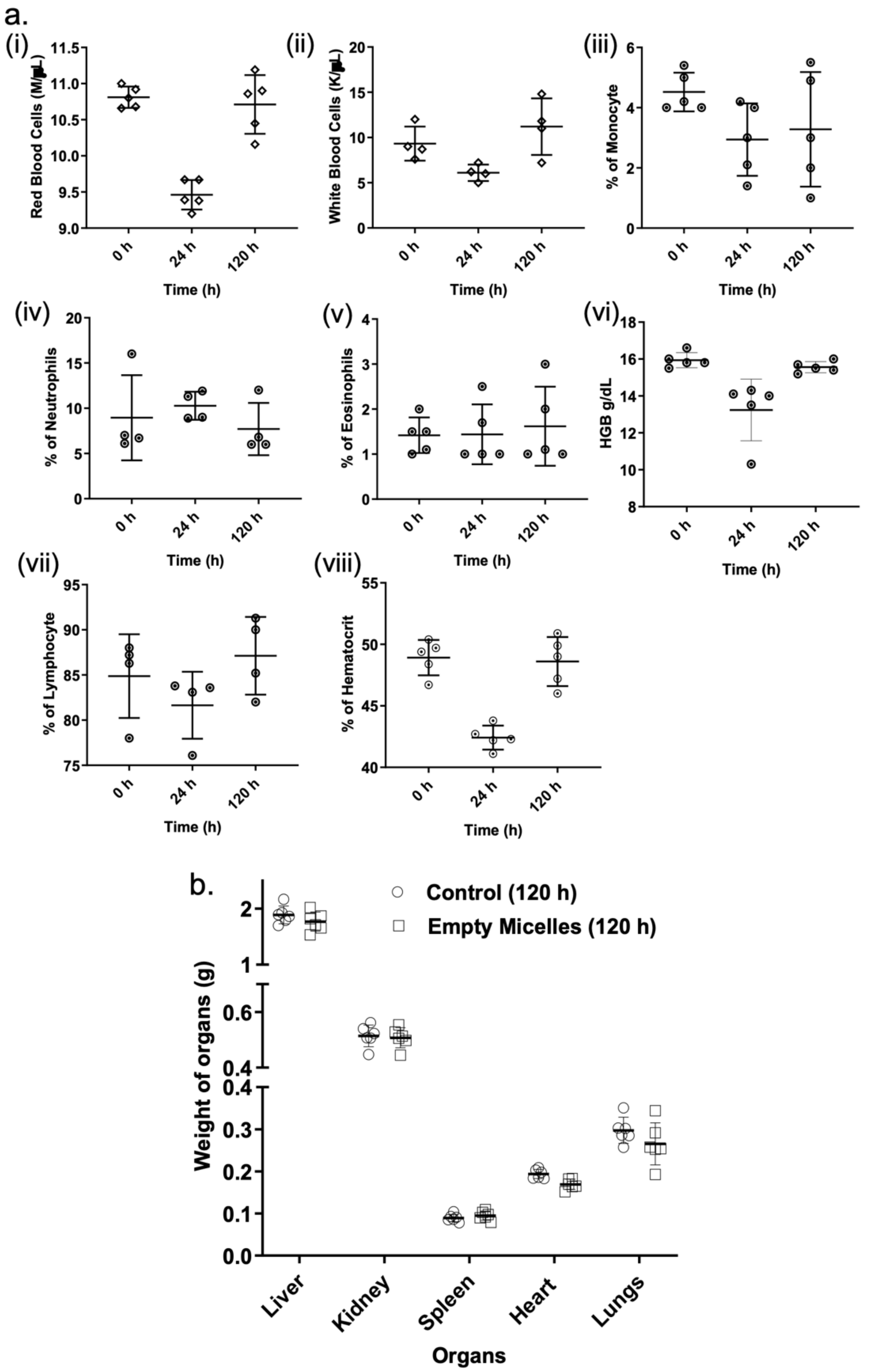

Figure S24. Acute toxicity analysis: a. Whole blood analysis (CBC results) (i) RBC (ii) WBC (iii) percentage of monocyte (iv) percentage of Lymphocyte (v) percentage of neutrophils (vi) percentage of eosinophils (vii) hemoglobin (viii) percentage of hematocrit. b. Total weight of organs (liver, kidney, spleen, heart, lungs) in grams.

Table S3: Numerical values of the CBC results illustrated in Figure S24.

Sl. No	CBC Parameters	Counts		
		0 h	24 h	120 h
1	Red Blood Cells (M/µL)	10.812±0.15	9.578±0.18	10.712±0.41
2	White Blood Cells (K/µL)	9.6±1.74	3.8±1.54	9.74±4.24
3	Lymphocytes (%)	85.28±4.12	82.7±3.98	88.1±4.32
4	Neutrophils (%)	8.3±4.33	10.22±1.35	8.42±3.48
5	Eosinophils (%)	1.42±0.40	1.44±0.67	1.62±0.88
6	Monocyte (%)	4.52±0.64	2.94±1.20	3.28±1.90
7	Hemoglobin (g/dL)	15.72±0.22	14.18±0.16	15.56±0.30
8	Hematocrit (%)	48.92±1.43	42.42±0.97	48.6±1.99

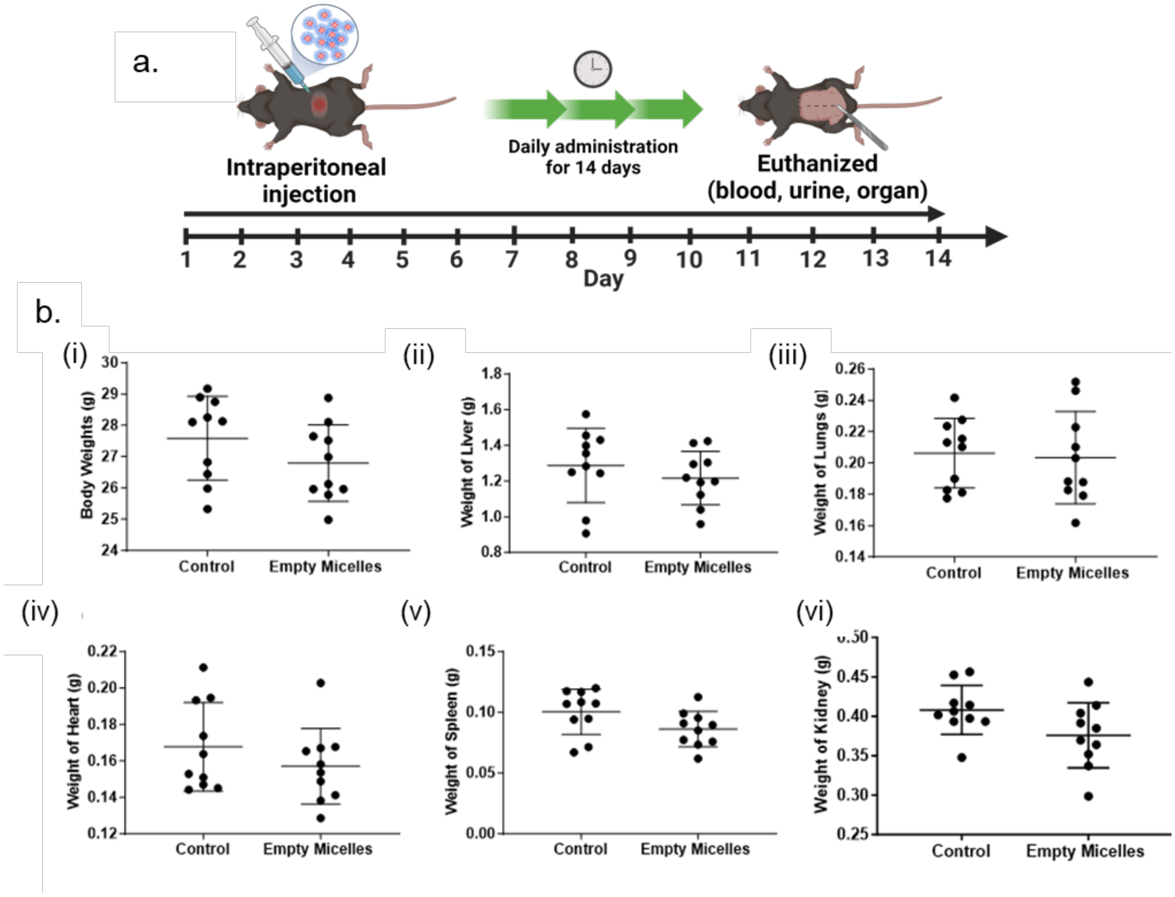

Figure S25. a) The schematic representation of the study design. Chronic toxicity analysis: b. (i) The body weight of the mice treated with saline (control) and empty micelle. The weight of the different organs in grams (ii) liver (iii) lungs (iv) heart (v) spleen (vi) kidney.

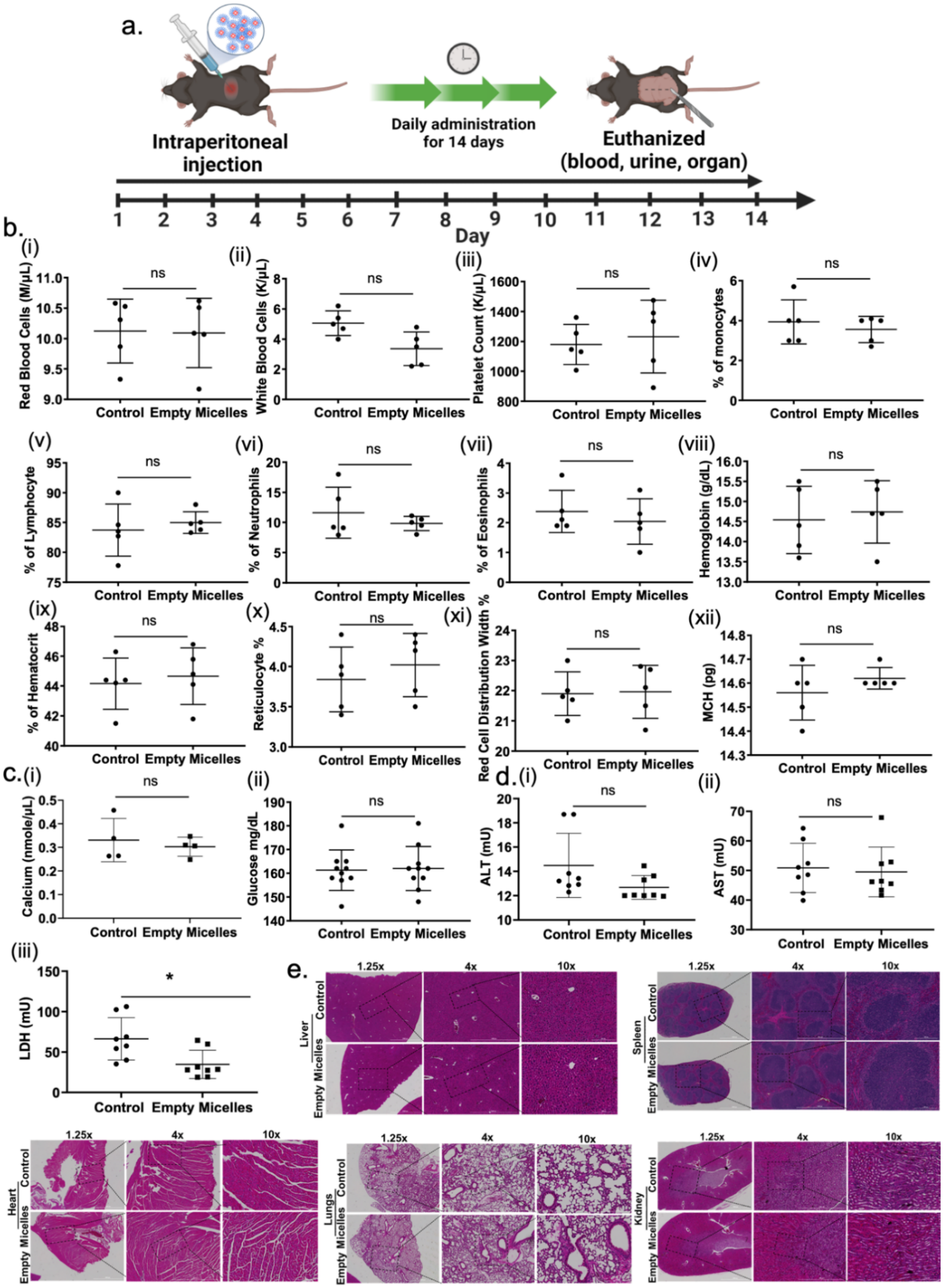

Figure S26. Chronic toxicity with daily injection of micelles for 14 days. a) The schematic representation of the study design. Chronic toxicity analysis: b. (i) The body weight of the mice treated with saline (control) and empty micelle. (b) Whole blood analysis (CBC) (n = 5) (i) RBC (ii) WBC (iii) Platelet count (iv) % monocytes (v) % Lymphocytes (vi) % Neutrophils (vii) % Eosinophils (viii) Hemoglobin (ix) % Hematocrit (x) % Reticulocyte (xi) % Red Cell Distribution Width (xii) Mean Corpuscular Hemoglobin (MCH). (c) Kidney function parameters (i) Calcium (n = 4) (ii) Glucose (n = 10). (d) Hepatic function parameters (i) ALT (ii) AST (iii) LDH (n = 8). (e) Hematoxylin and eosin staining of the liver, spleen, heart, lungs, and kidney (magnification 1.25x, 4x, 10x). $p < 0.05$ (*)

Table S4. Numerical values of the CBC results illustrated in Figure S26.

SI. No	CBC Parameters	Counts	
		Control	Micelles
1	Red Blood Cells (M/µL)	10.12±0.52	10.09±0.57
2	White Blood Cells (K/µL)	4.66±0.57	3.36±1.11
3	Lymphocytes (%)	83.72±4.37	84.98±1.83
4	Neutrophils (%)	11.62±4.24	9.82±1.18
5	Eosinophils (%)	2.38±0.71	1.62±0.59
6	Monocyte (%)	4.34±1.04	3.56±0.66
7	Hemoglobin (g/dL)	14.54±0.84	14.74±0.78
8	Hematocrit (%)	44.82±2.35	44.66±1.90
9	Mean Platelet Volume (fL)	7.64±0.30	7.5±0.19
10	Reticulocyte (%)	3.76±0.53	4.02±0.40
11	Red Cell Distribution width (%)	21.64±0.38	22.16±0.58
12	Platelet Count (K/µL)	1180±134	1232±243
13	Mean Corpuscular Hemoglobin (pg)	14.56±0.11	14.62±0.04
14	Mean Corpuscular Hemoglobin Concentration (g/dL)	32.44±0.67	32.98±0.44

Table S5. Hepatic function parameter values as illustrated in Figure S26.

SI. No	Hepatic Function Parameters	Counts (mU)	
		Control	Empty micelles
1	Aspartate aminotransferase (AST)	50.85 ± 8.33	49.50 ± 8.39
2	Alanine aminotransferase (ALT)	14.48 ± 2.64	12.68 ± 0.97
3	Lactate dehydrogenase (LDH)	66.28 ± 26.16	34.71 ± 17.50

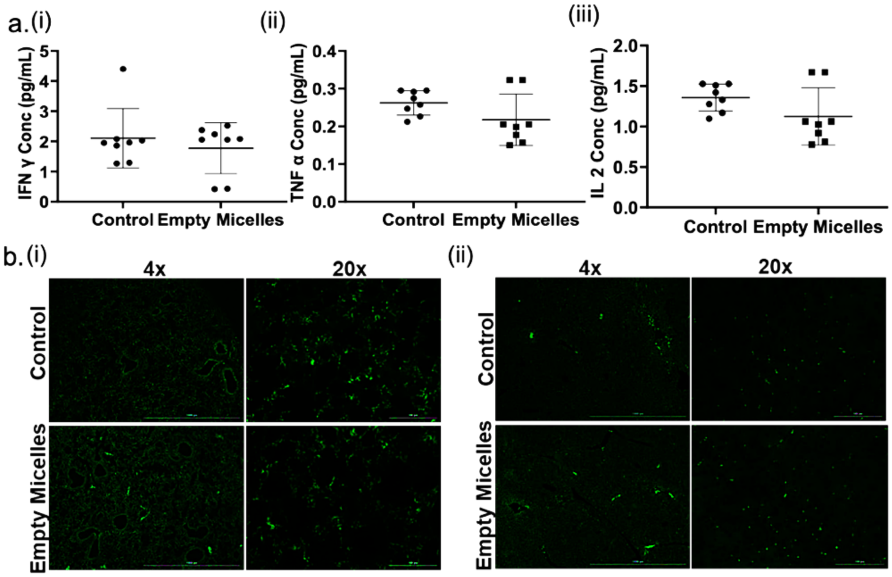

Figure S27. (a) Quantification of the immune markers (i) IFN γ (ii) TNF α and IL2 (n = 8). (b) Immunochemistry to show the tissue-specific markers by using F8/40 antibody (i) lungs (ii) liver.

Table S6. Inflammatory/immune marker values as illustrated in Figure 27.

SI. No	Inflammatory/immune markers	Counts (pg/mL)	
		Control	Empty micelles
1	IFN γ	2.11 ± 0.98	1.77 ± 0.85
2	TNF α	0.26 ± 0.03	0.21 ± 0.07
3	IL 2	1.35 ± 0.17	1.12 ± 0.35

***In vivo* µPET/µCT Imaging of PEG-*b*-P(NIPAM-*stat*-2-APBA) Micelle and GCG-SH.**

µPET/µCT ^{89}Zr-labeled micelle/^{89}Zr -labeled linear polymer.

After confirming the general safety profile of the micelle, µPET/µCT imaging was performed to gain deeper insights into the *in vivo* biodistribution and pharmacokinetic profiles of the polymeric system. The polymer is expected to transition between its micellar and linear forms in response to glucose concentration during *in vivo* studies. Examining the differences between both forms allows for an understanding of the complete picture of biodistribution and pharmacokinetic properties of the system. Therefore, two different polymer batches were synthesized: one always in the micelle state and another always in the linear state at 37 °C regardless of glucose concentration. Both PEG-*b*-P(NIPAM-*stat*-2-APBA) polymer end groups were aminolyzed to a free thiol and conjugated with deferoxamine-maleimide (DFO-maleimide), a robust chelator of the radiometal zirconium-89 (^{89}Zr) (**Figure S28**). DFO-conjugated polymers were chelated with [^{89}Zr]Zr-oxalate at room temperature, and upon purification, DLS analysis was used to confirm the formation of the ^{89}Zr-labeled micelle and ^{89}Zr-labeled linear polymer (**Figure S29a,b**).

The µPET/µCT imaging analysis revealed that the ^{89}Zr-labeled micelle maintained a higher concentration in the blood. In contrast, the renal clearance rate of the ^{89}Zr-labeled linear polymer was accelerated during the first-hour post-injection (**Figure S29-S31**). Compared with the ^{89}Zr-labeled linear polymer, the ^{89}Zr-labeled micelle showed higher uptake at 2 h post-injection by the liver, spleen, intestine (GI) and lungs (**Figures S29c-n, Table S7**). At the endpoint of the experiment (192 h), both forms accumulated in the liver, spleen, and kidney, with the ^{89}Zr-labeled micelle at a higher concentration than the ^{89}Zr-labeled linear polymer (**Figure S32**). The PET results were different than what was observed in the toxicity studies where it was confirmed the absence of inflammation or immune cell activation in response to polymer injection. We hypothesized that the large molecular weight and relative hydrophobicity of the [^{89}Zr]Zr-DFO label when conjugated at the polymer-end group could potentially contribute to the observed biodistribution. To test this hypothesis, additional µPET/µCT imaging experiments were performed using ^{18}F-labeling *via* a prosthetic group (^{18}F-FBEM or ^{18}F-SFB), which allowed labeling of the polymer directly without the use of a radiometal or chelating ligand. We expected the small size of the radio-fluorinated prosthetic group, and its reduced hydrophobicity compared to [^{89}Zr]Zr-DFO to impact the polymer structure minimally, better representing the true biodistribution of the micelle. The data is presented below.

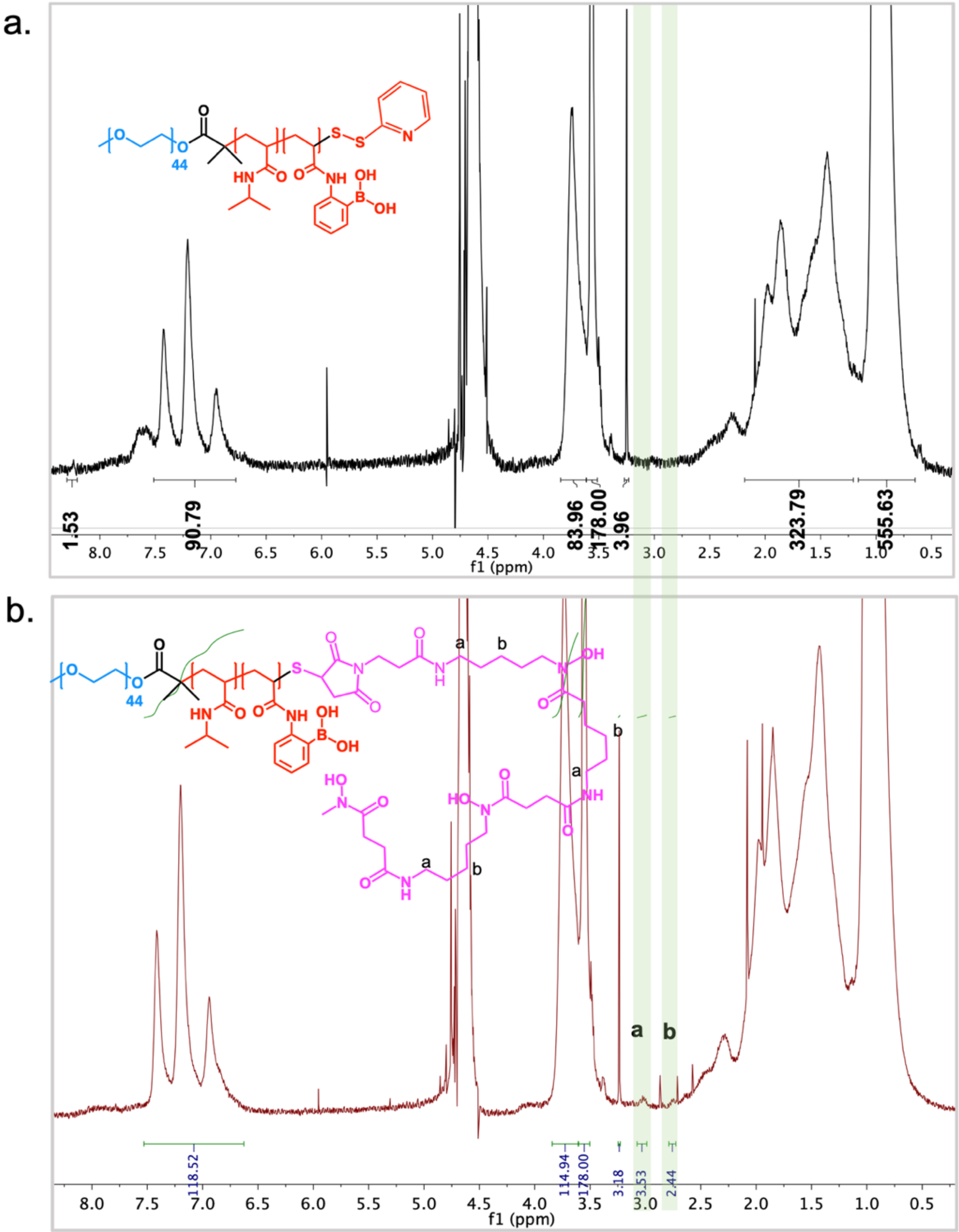

Figure S28. ^{1}H NMR spectrum (a) PEG-*b*-P(NIPAM-*stat*-2-APBA)-PDS (P2) and (b) PEG-*b*-P(NIPAM-*stat*-2-APBA) upon post-modification with deferoxamine (DFO) recorded in D_2O (400 MHz).

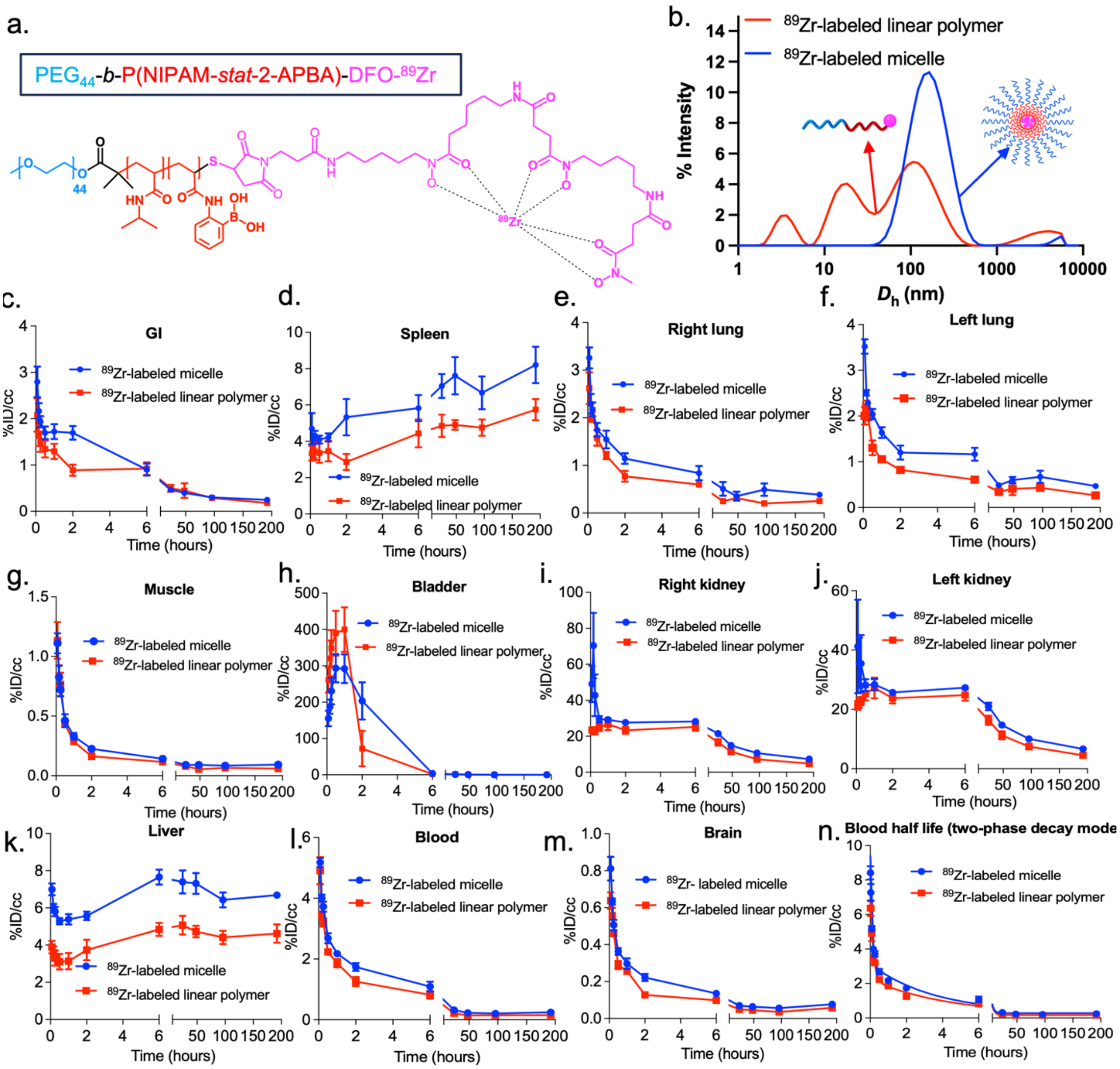

Figure S29. μPET/μCT analysis of ^{89}Zr-labeled linear polymer and ^{89}Zr-labeled micelle. (a) Chemical structure of ^{89}Zr-chelated PEG-*b*-P(NIPAM-*stat*-2-APBA)-DFO. (b) Intensity-weighted size distributions obtained by DLS for ^{89}Zr-labeled linear polymer and ^{89}Zr-labeled micelle. (c) Time course biodistribution and excretion of fresh ^{89}Zr-labeled linear polymer (red) and ^{89}Zr-labeled micelle (blue) from (c) gastrointestinal (GI), (d) spleen, (e) right lung, (f) left lung, (g) muscle, (h) bladder, (i) right kidney, (j) left kidney, (k) liver, (l) blood, (m) brain and (n) blood half time of eight C57BL/6 male (n = 8). Note the scale of %ID/cc differs between each organ. ID, injected dose. CC, cubic centimeter.

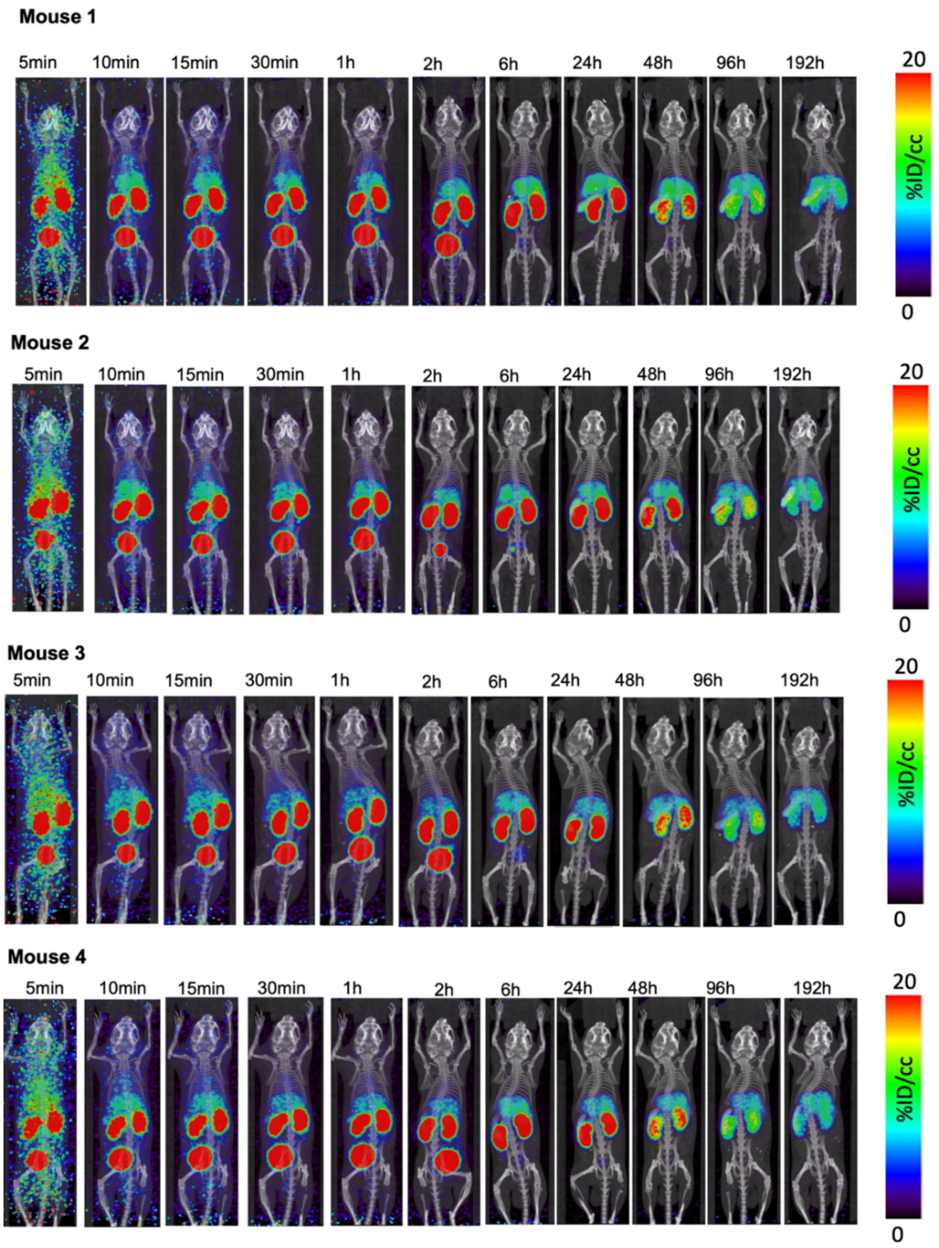

Figure S30. Co-registered μPET/μCT scans post ^{89}Zr-labeled micelle injection to four C57BL/6 male mice. ID, injected dose. CC, cubic centimeter.

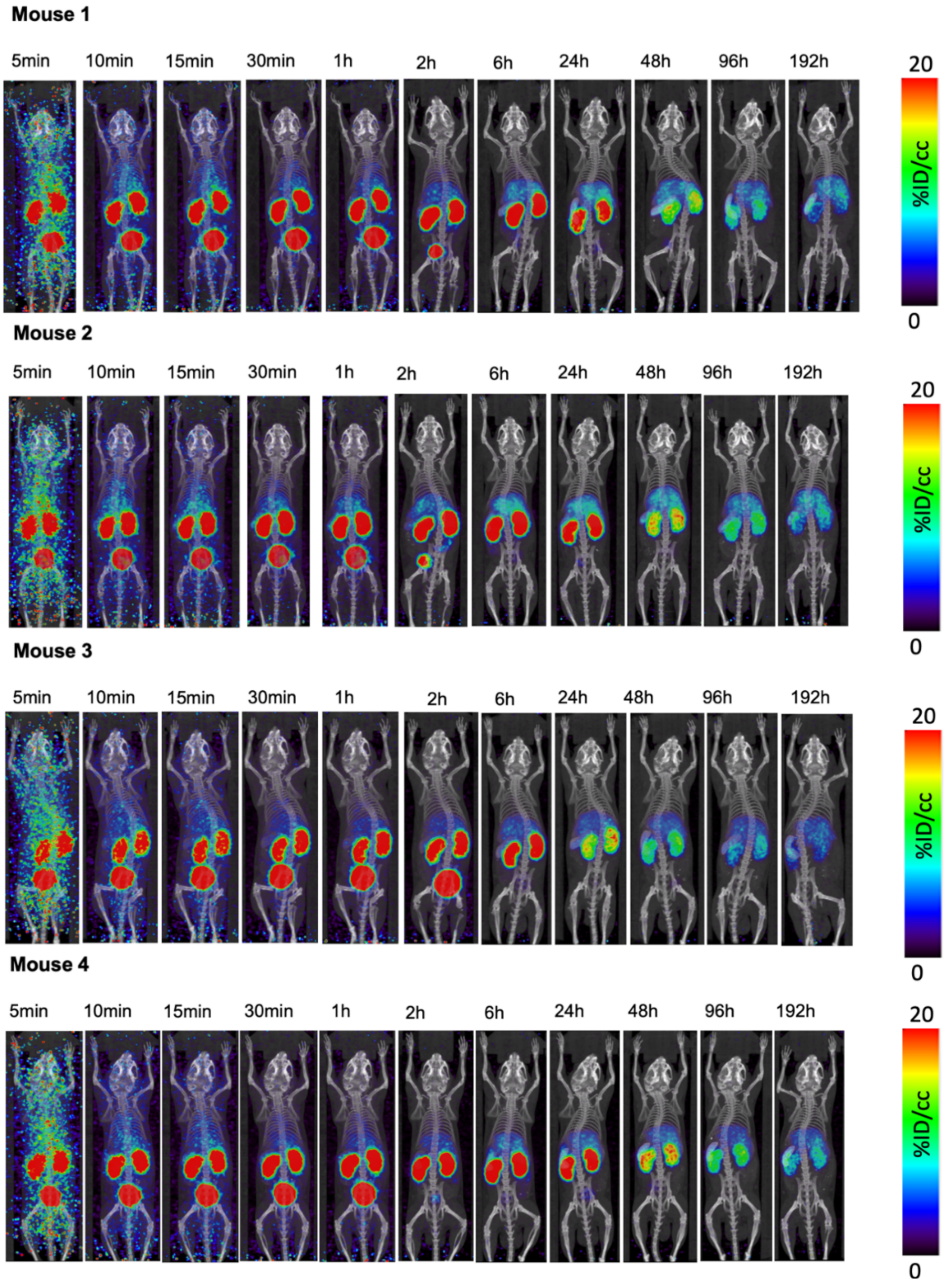

Figure S31. Co-registered μPET/μCT scans post ^{89}Zr-labeled linear polymer injection to four C57BL/6 male mice. ID, injected dose. CC, cubic centimeter.

Table S7. Quantification of µPET/µCT scans for ^{89}Zr-labeled linear polymer and ^{89}Zr-labeled micelle 2h post-injection from the organs showing the highest uptake, with appreciable signal. ID, injected dose. CC, cubic centimeter.

**Organ**	**%ID/cc**	
	**^{89}Zr-labeled linear polymer**	**^{89}Zr-labeled micelle**
Liver	3.74 ± 1.11	5.57 ± 1.25
Spleen	2.86 ± 0.90	5.33 ± 2.01
GI	0.89 ± 0.24	1.69 ± 0.30
Right lung	0.77 ± 0.23	1.14 ± 0.22
Left lung	0.82 ± 0.057	1.20 ± 0.31

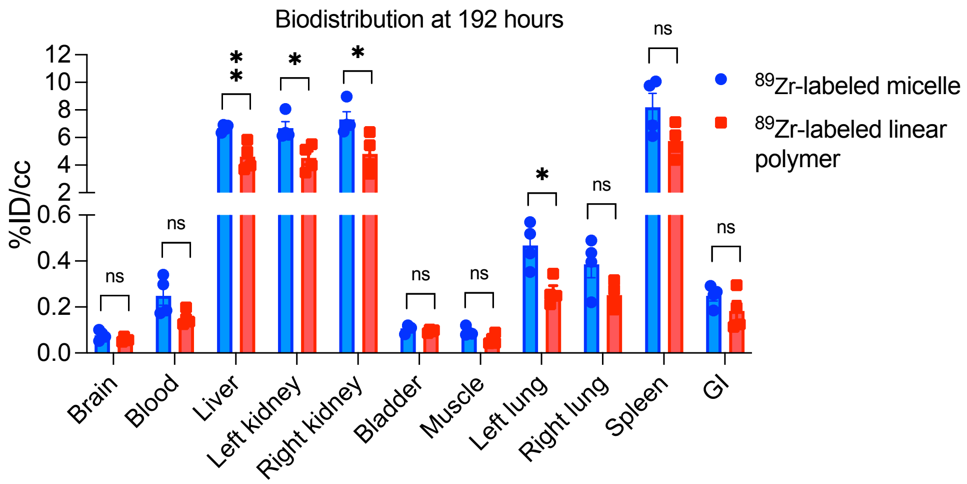

Figure S32. Quantification of µPET/µCT scans for ^{89}Zr-labeled linear polymer and ^{89}Zr-labeled micelle 192h post-injection from the organs with appreciable signal. ID, injected dose. CC, cubic centimeter.

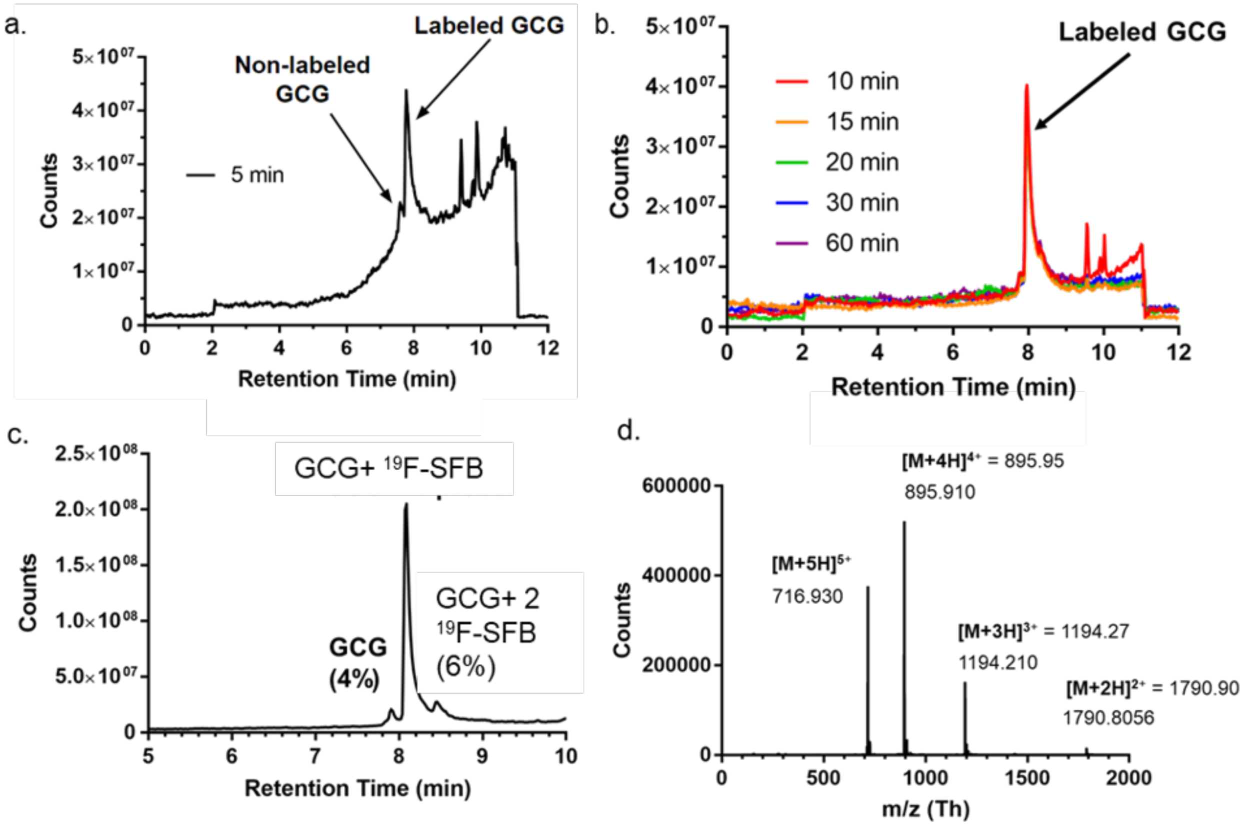

Figure S33. Conjugation of non-radioactive ^{19}F-SFB prosthetic group to GCG. (a) After 5 minutes, GCG is almost entirely labeled with ^{19}F-SFB. (b) Conjugation after 10 minutes is complete with no further conversion up to 60 minutes. (c) After purification by centrifugal filtration (MWCO = 3 kDa), ^{19}F-SFB-labeled GCG (1 SFB) is the primary species in solution. A small percentage of GCG is unlabeled or labeled with 2 SFB consistent with the 2 lysine residues present in GCG sequence. (d) LC-MS confirms the presence of the ^{19}F-SFB-labeled GCG conjugate.

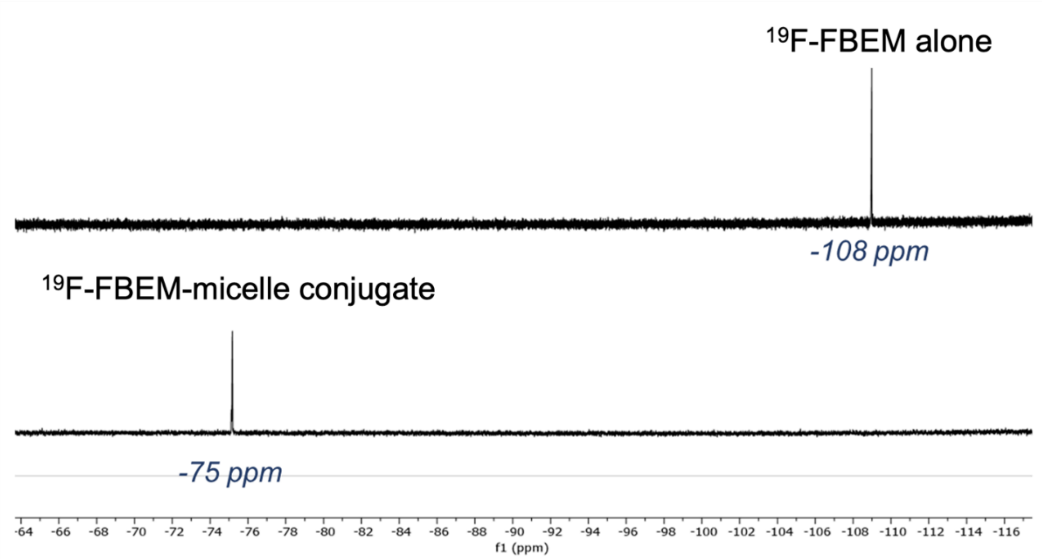

Figure S34. ^{19}F-NMR with proton decoupling recorded in D_2O. Conjugation of ^{19}F-FBEM prosthetic group to the micelle. The FBEM alone appears at δ = -108 ppm and shifts to δ = -75 ppm after conjugation to the micelle.

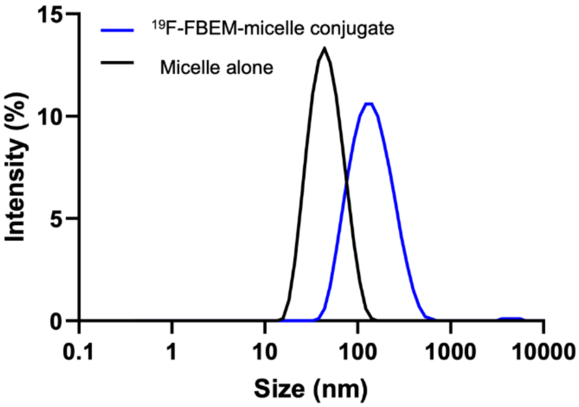

Figure S35. Intensity-weighted size distributions by DLS of micelle at 37 °C prior to labeling (black) and after labeling with non-radioactive ^{19}F-FBEM (blue).

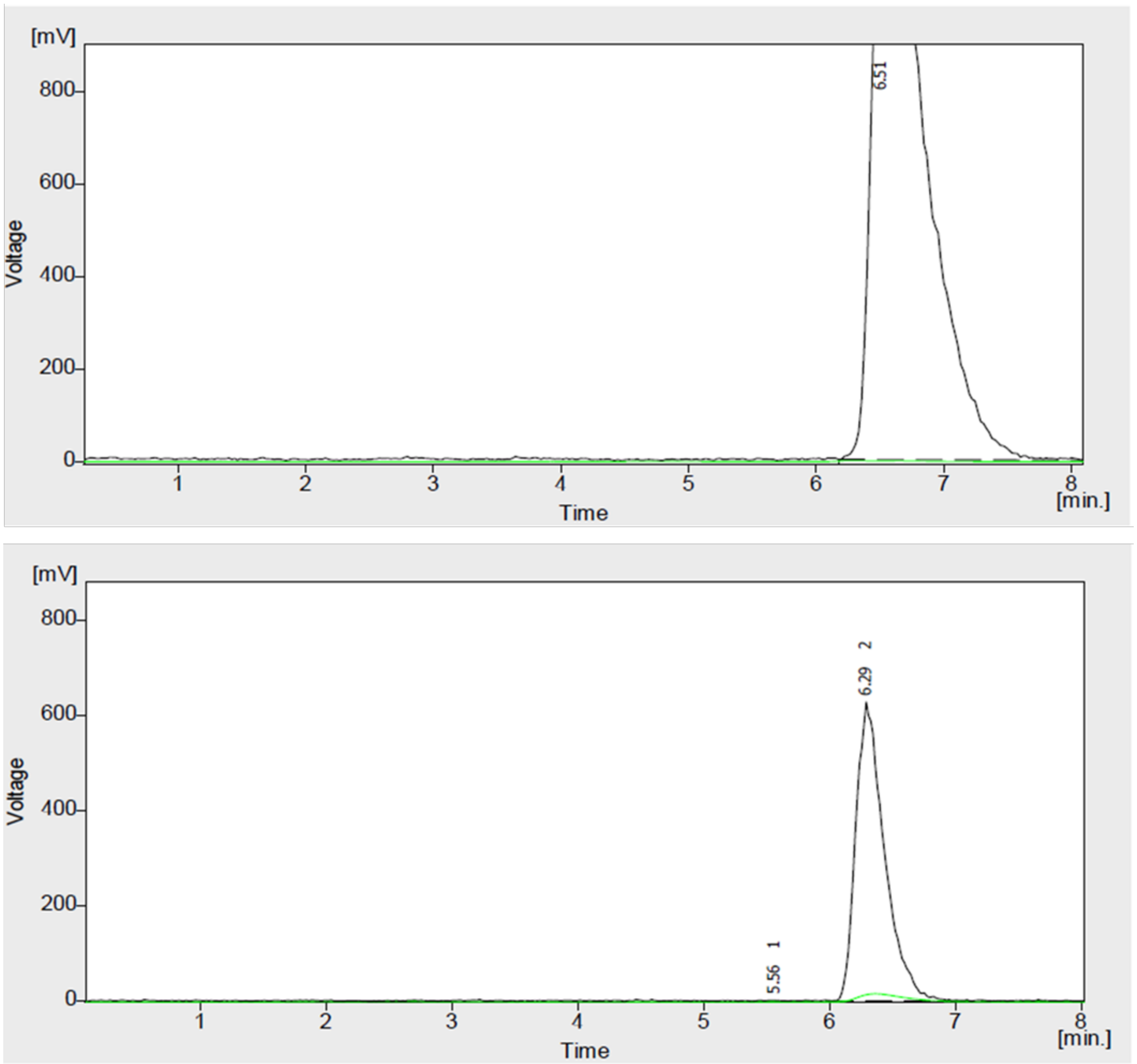

Figure S36. HPLC chromatogram of (a) purified ^{18}F-SFB and (b) purified ^{18}F-SFB co-injected with SFB reference standard. Column: Column: Phenomenex C18 5 µ Luna 250 x 4.6 mm; Mobile phase: 50% v/v MeCN in H_2O (0.1% TFA); flow rate: 1 mL/min.

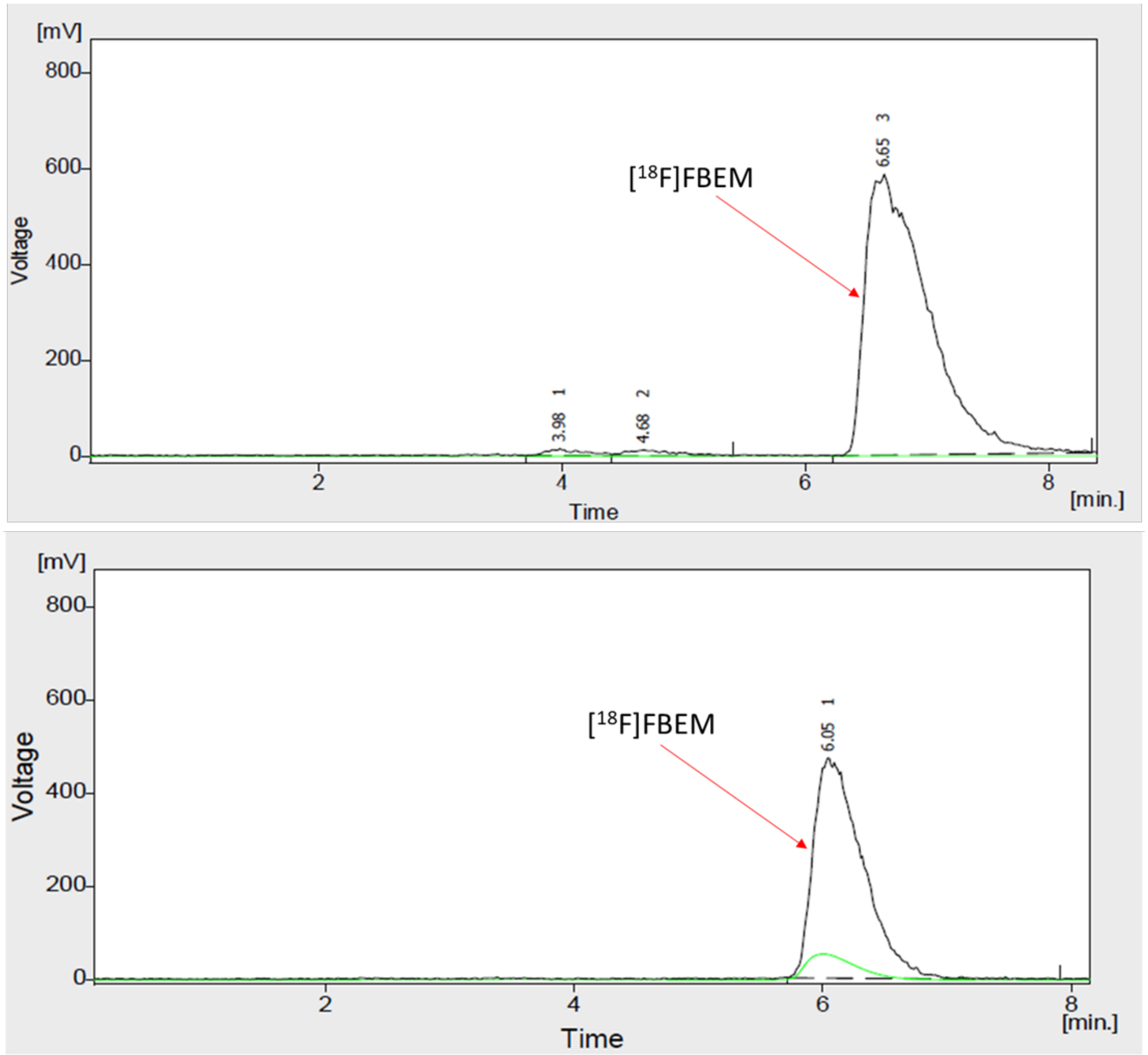

Figure S37. HPLC chromatogram of (a) purified ^{18}F-FBEM and (b) purified ^{18}F-FBEM co-injected with FBEM reference standard. Column: Column: Phenomenex C18 5 µ Luna 250 x 4.6 mm; Mobile phase: 20% v/v MeCN in H_2O; flow rate: 1.5 mL/min.

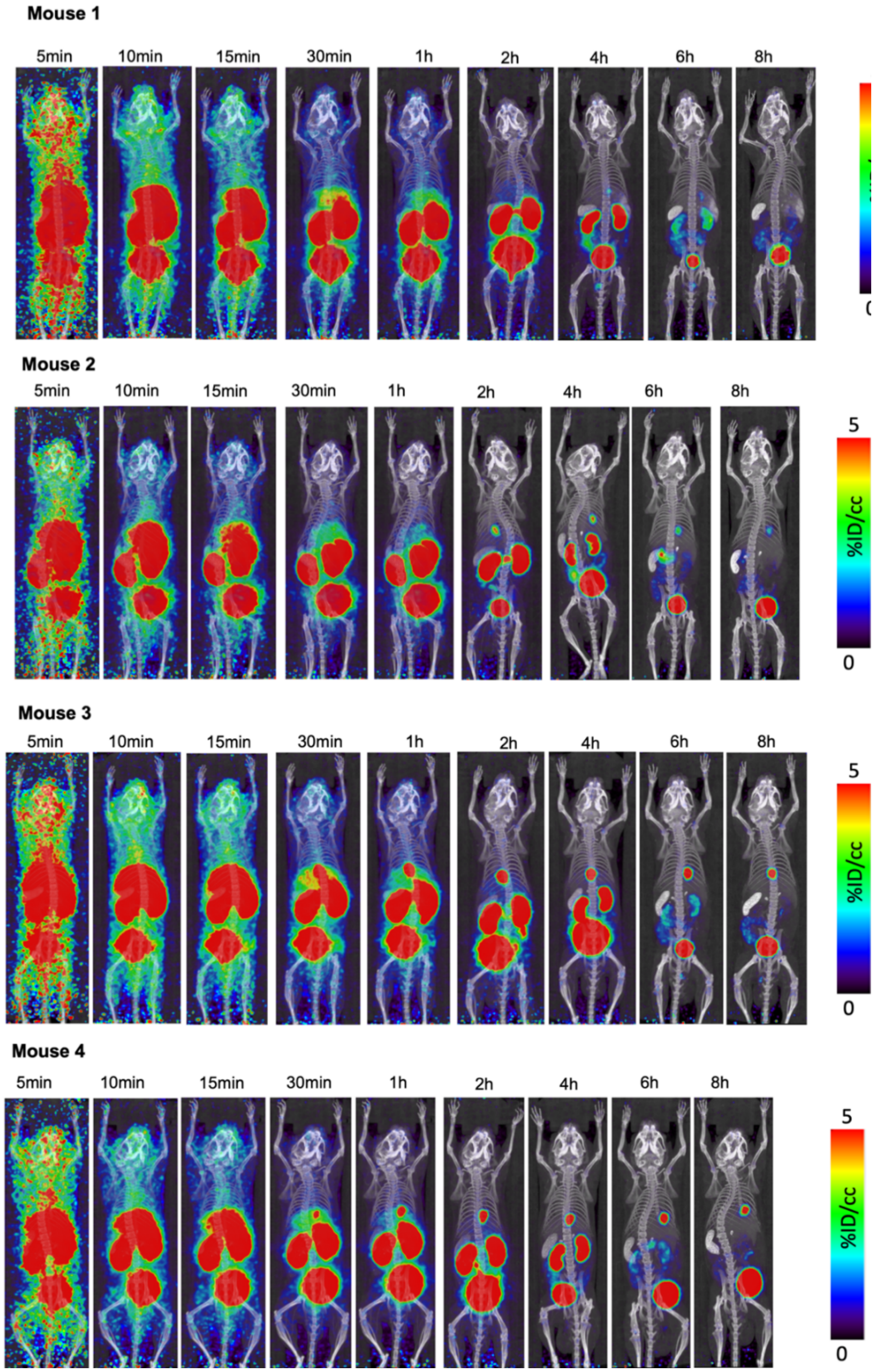

Figure S38. Co-registered μPET and μCT scans post ^{18}F-SFB-labeled glucagon injection to four C57BL/6 male mice. ID, injected dose. CC, cubic centimeter.

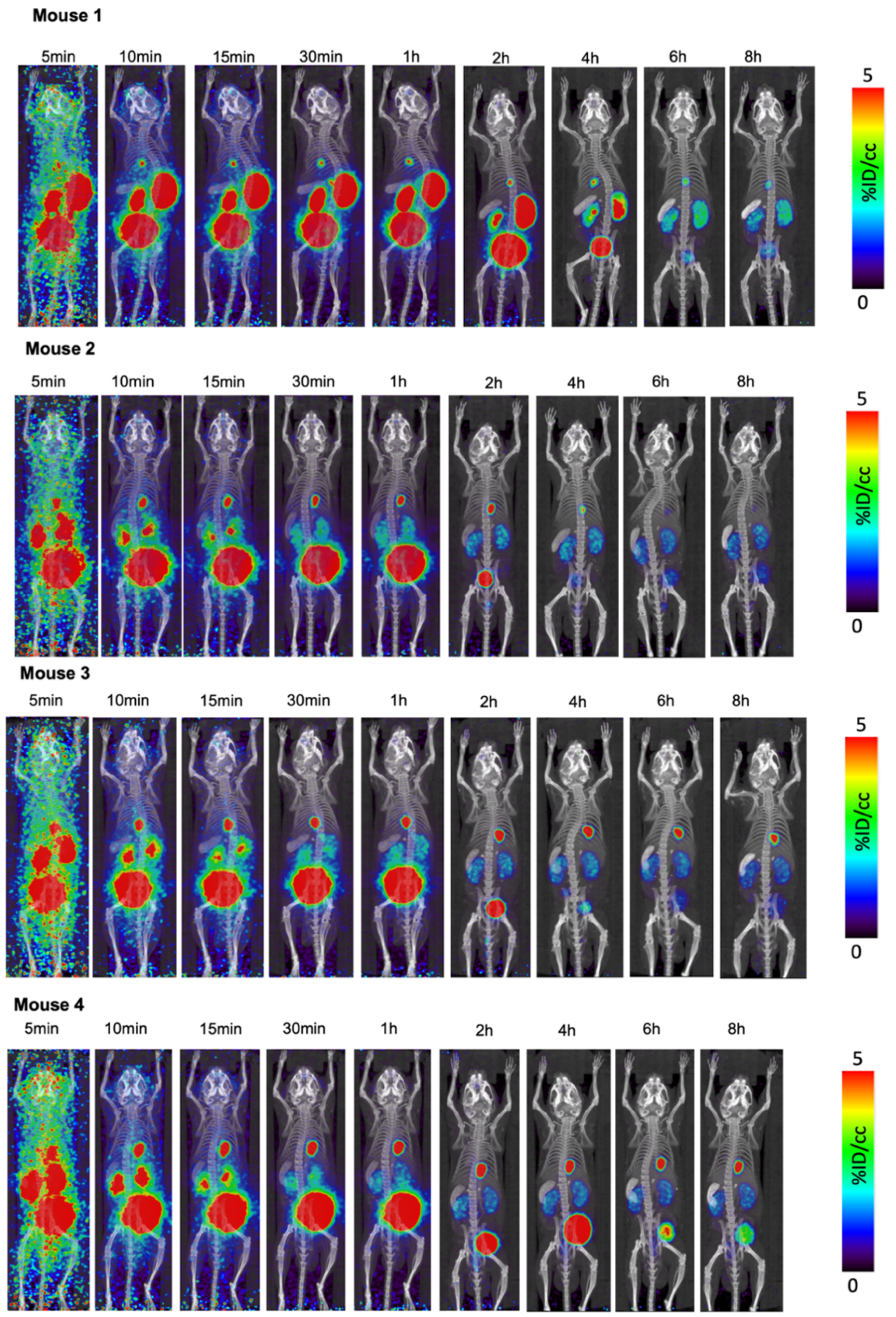

Figure S39. Co-registered μPET and μCT scans post ^{18}F-FBEM-labeled micelle injection to four C57BL/6 male mice. ID, injected dose. CC, cubic centimeter.

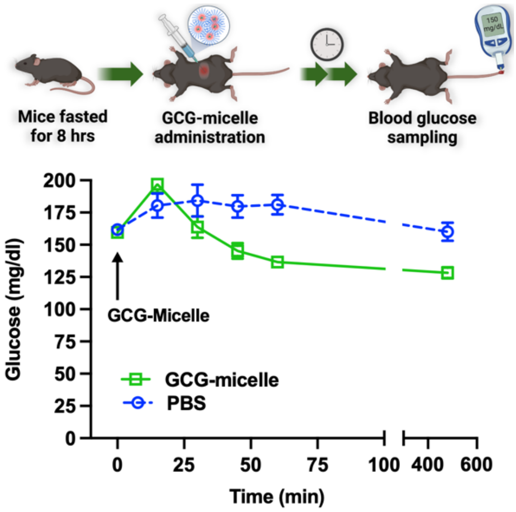

Figure S40. Impact of micelles on glycemia in male C57Bl/6J mice over 8 h. Data is represented as mean ± SEM, n = 5-6.

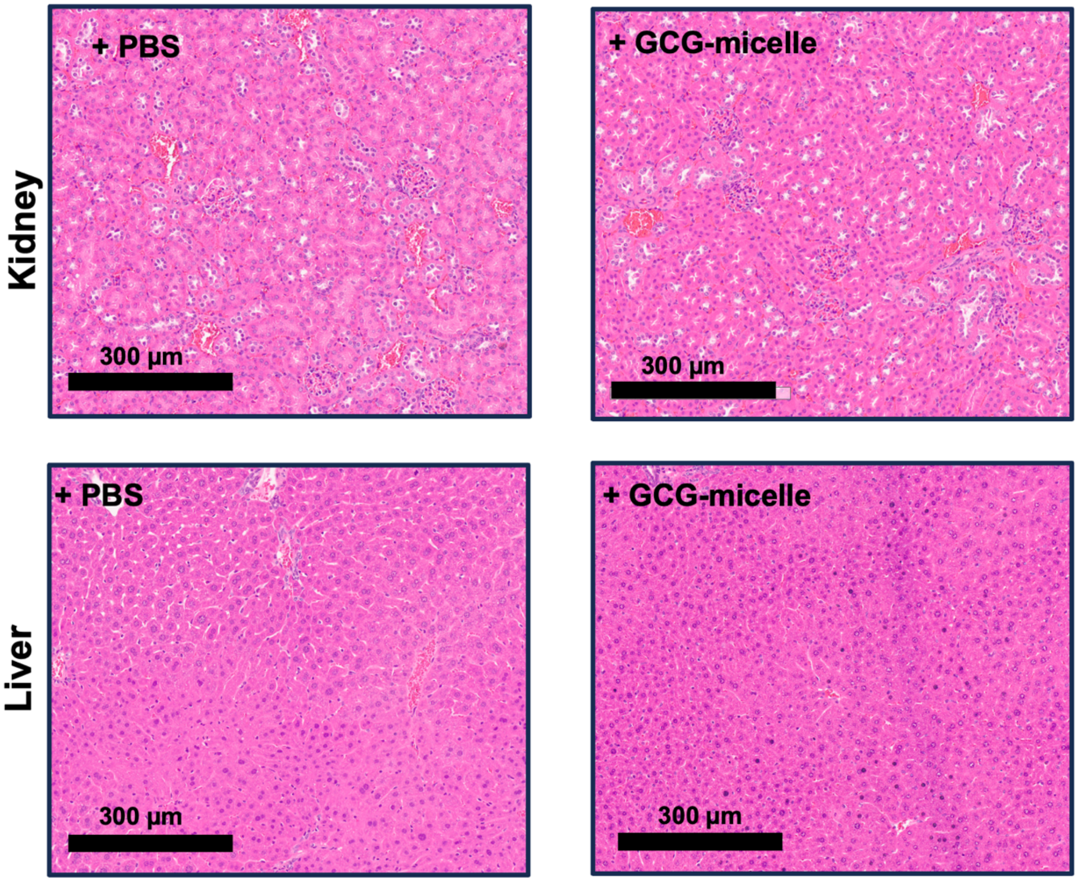

Figure S41. H&E staining of kidney and liver tissue of C57Bl/6J mice treated with GCG-micelle (P2-GCG) or PBS.

***In vivo* Activity of native and thiolated glucagon**

A separate study was conducted to evaluate the efficacy of native glucagon and thiolated glucagon in reversing insulin-induced deep hypoglycemia. This efficacy study was conducted in healthy C57Bl/6J male mice (age 14 weeks). Mice were fasted for 12 hours before the administration of insulin at a dose of 0.90 U/kg intraperitoneally to induce hypoglycemia as a consequence of liver glycogen depletion. Glucose levels decreased from approximately 100 to 60 mg/dL after 60 minutes of insulin administration. Subsequently, GCG-native and GCG-SH at a dose of 500 µg/kg of glucagon were administered intraperitoneally. Blood glucose levels immediately increased, restoring baseline glycemia within 40 minutes for GCG-native and within 50 minutes for GCG-SH administration (**Figure S42**).

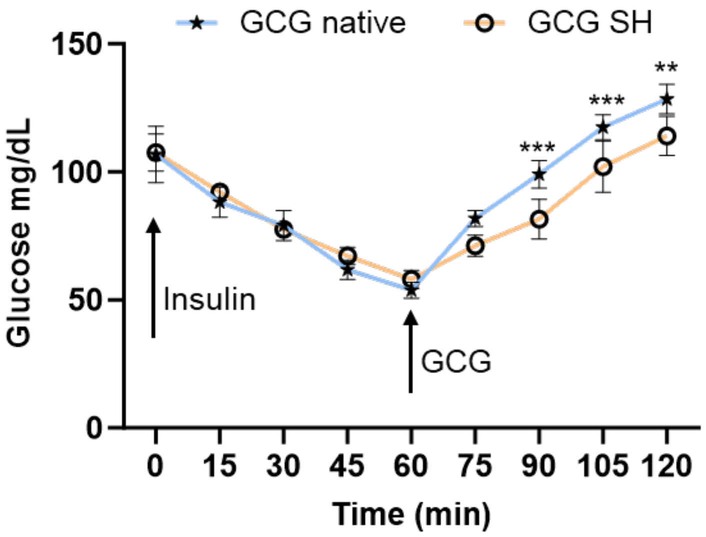

Figure S42: Impact of glucagon (native and thiolated) on reversal of insulin-induced deep hypoglycemia in fasted C57Bl/6J mice. Data is represented as mean ± SEM, n = 6. $p < 0.05$ (*), $p < 0.01$ (**), $p < 0.001$ (***), $p < 0.0001$ (****) within group comparison (native vs thiolated glucagon) of glucose levels at each time point.

References

[1] B. V. K. J. Schmidt, M. Hetzer, H. Ritter, C. Barner-Kowollik, *Macromolecules* **2011**, *44*, 7220.

[2] R. Tavaré, H. Escuin-Ordinas, S. Mok, M. N. McCracken, K. A. Zettlitz, F. B. Salazar, O. N. Witte, A. Ribas, A. M. Wu, *Cancer Res* **2016**, *76*, 73.

[3] J. Collins, C. M. Waldmann, C. Drake, R. Slavik, N. S. Ha, M. Sergeev, M. Lazari, B. Shen, F. T. Chin, M. Moore, S. Sadeghi, M. E. Phelps, J. M. Murphy, R. M. van Dam, *Proceedings of the National Academy of Sciences* **2017**, *114*, 11309.

[4] M. Lazari, J. Collins, B. Shen, M. Farhoud, D. Yeh, B. Maraglia, F. T. Chin, D. A. Nathanson, M. Moore, R. M. van Dam, *J Nucl Med Technol* **2014**, *42*, 203.

[5] M. Varadi, S. Anyango, M. Deshpande, S. Nair, C. Natassia, G. Yordanova, D. Yuan, O. Stroe, G. Wood, A. Laydon, A. Žídek, T. Green, K. Tunyasuvunakool, S. Petersen, J. Jumper, E. Clancy, R. Green, A. Vora, M. Lutfi, M. Figurnov, A. Cowie, N. Hobbs, P. Kohli, G. Kleywegt, E. Birney, D. Hassabis, S. Velankar, *Nucleic Acids Res* **2022**, *50*, D439.

[6] J. Jumper, R. Evans, A. Pritzel, T. Green, M. Figurnov, O. Ronneberger, K. Tunyasuvunakool, R. Bates, A. Žídek, A. Potapenko, A. Bridgland, C. Meyer, S. A. A. Kohl, A. J. Ballard, A. Cowie, B. Romera-Paredes, S. Nikolov, R. Jain, J. Adler, T. Back, S. Petersen, D. Reiman, E. Clancy, M. Zielinski, M. Steinegger, M. Pacholska, T. Berghammer, S. Bodenstein, D. Silver, O. Vinyals, A. W. Senior, K. Kavukcuoglu, P. Kohli, D. Hassabis, *Nature* **2021**, *596*, 583.
